# Synthesis and characterization of NFA-based polymers for solar cells with improved thermal stability

**DOI:** 10.1039/d5tc02570b

**Published:** 2025-09-25

**Authors:** Lucie Rivet, Antoine Curé, Camille Jutard, Samuel Fauvel, Renaud Demadrille, Antonio J. Riquelme, Cyril Aumaître

**Affiliations:** a IRIG-SyMMES, Université Grenoble Alpes/CEA/CNRS/Grenoble INP 38000 Grenoble France cyril.aumaitre@cea.fr

## Abstract

Among the latest advances in organic solar cells, all-polymer solar cells based on non-fullerene acceptors (NFAs) are emerging as a promising way to improve device stability. However, the synthesis of new electron-accepting polymers suitable for the active layer remains relatively unexplored. Current efforts primarily focus on maximizing photovoltaic conversion efficiency using PY-IT, a polymer derived from Y6. By contrast, there is a lack of fundamental research into controlling polymerization processes and the effect of the intrinsic optoelectronic properties of NFA-based polymers on their reactivity and stability when subjected to thermal stress and light soaking. To address this, we present the synthesis of a series of NFA-based polymers that incorporate thiophene, indacenodithiophene or a thienothiophene analogue. We systematically optimized Stille polymerisation by evaluating a range of phosphine ligands and correlating their performance with Tollman electronic and steric parameters, an approach that has rarely been explored in the literature. The resulting polymers exhibit improved macromolecular control, good solubility in *o*-xylene and optical properties suited to integration into the active layer of solar cells. Comprehensive spectroscopic and morphological characterisation (UV-vis, AFM and GIWAXS) of pristine polymer films confirms their amorphous nature in the solid state. The thermal and photochemical stability of the three new polymers was evaluated in devices under ISOS-D-2 (thermal ageing) and ISOS-L-1 (light soaking) protocols. After 1000 hours of thermal stress, all devices retained over 90% of their initial efficiency and they also demonstrated outstanding photostability over 300 hours under 1 Sun illumination. Some materials showed no degradation under these conditions, highlighting the potential of all-polymer solar cells to overcome long-standing stability challenges in organic photovoltaics.

## Abstract 2

Design of polymers *via* Stille polymerization enables high-performance organic solar cells with improved stability, processability, and compatibility with non-halogenated solvents.

## Graphical abstract

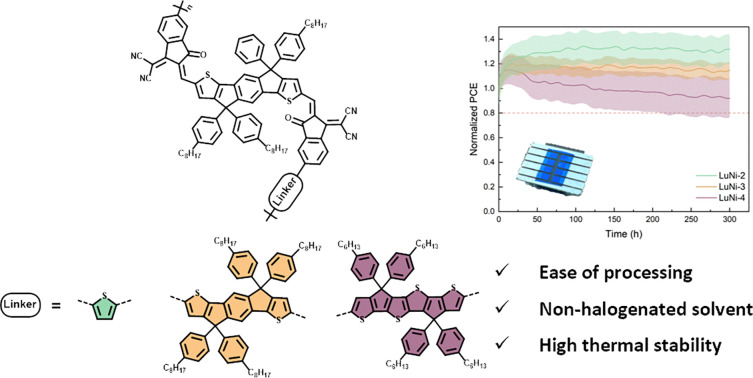

## Introduction

In recent years, there has been growing interest in the development of all-polymer solar cells (APSCs) within the field of organic photovoltaics. Unlike traditional blends that combine an electron-donating polymer with a small-molecule electron acceptor, APSCs utilize two polymers in the active layer, one serving as the donor and the other as the acceptor. This strategy is particularly appealing due to the wide structural diversity and tunability of polymeric materials.^1–3^ Compared to small-molecule acceptors, including non-fullerene acceptors (NFAs), polymer-based materials offer enhanced mechanical and thermal stability, as well as higher solution viscosity, which can be advantageous during device fabrication.^4,5^ These properties enable the fabrication of thicker active layers and more mechanically robust, flexible devices, which are particularly advantageous for scalable manufacturing and wearable applications.^6–8^ The performance of devices was limited for a long time by n-type polymers due to their low electron mobility and weak absorption coefficients, as well as the challenges involved in controlling their miscibility with the other components of the active layer.^9^ However, N2200 marked a turning point in the development of all-polymer solar cells, enabling a record efficiency of 11.76% in 2019, thanks to the meticulous optimization of the deposition process.^10^ Following the emergence of non-fullerene acceptors (NFAs) in 2015, Yongfang Li's group quickly moved to polymerize a monomer structurally related to the well-known NFA IDIC. This monomer featured an indacenothiophene (IDT) central core, comprising an indacenothiophene (IDT) central core and a mixture of 2-(5-bromo-3-oxo-2,3-dihydro-1*H*-inden-1-ylidene)malononitrile (δ-IC-Br) and 2-(6-bromo-3-oxo-2,3-dihydro-1*H*-inden-1-ylidene)malononitrile (γ-IC-Br) as flanking groups. This breakthrough led to the synthesis of PZ1, one of the first polymers to feature a non-fullerene acceptor structure.^11^ Compared to N2200, PZ1 exhibited superior light absorption and enhanced charge carrier mobilities, resulting in a promising power conversion efficiency (PCE) of 9.19%. Building on this success, the synthesis of n-type polymers was subsequently extended to non-fullerene acceptors (NFAs) based on indacenothienothiophene (IDTT) cores such as ITIC^12^ and dithienothiophen[3,2-*b*]-pyrrolobenzothiadiazole (PY-IT, Y6^13–15^) central cores. A record PCE of 20.8% was recently reported with the use of PY-IT, in conjunction with small-molecule non-fullerene acceptors.^16,17^ The use of NFA based polymer is also expected to improve the stability of the devices. Indeed, in small-molecule NFA-based blends, the crystallization of the NFAs is often responsible for the thermal drift of the active layer morphology, and significant improvements have been demonstrated with polymeric materials.^18^ To fine-tune solubility and control crystallization that are key parameters governing film morphology and device efficiency, NFA-based polymers are often designed with heterocyclic co-monomers such as thiophene, furan, thiazole, or vinylene units, and they occasionally include non-conjugated spacers.^1,19–22^ The choice of co-monomers has been shown to significantly influence the electronic energy levels, intermolecular interactions, and optical bandgap of the resulting material.^2,23,24^ However, the influence of these co-monomers on the stability of the metastable bulk heterojunction (BHJ) morphology remains not fully understood. For instance, Min's research group investigated a series of PY-IT polymers and reported significant differences in photostability: after 600 hours of illumination, polymers containing selenophene *versus* thiophene co-monomers exhibited a 25% variation in performance retention.^22^ While photochemical stability warrants further study, recent advances have started to clarify rational chemical design principles for NFAs. For example, protecting the β-position of the lateral thiophene units in the IDT and IDTT central cores has been demonstrated to enhance photostability.^25^ In general, the all-polymer strategy relies on the entanglement of polymer chains to create a more thermodynamically stable nanomorphology.^26–28^ Overall, the all-polymer approach relies on the entanglement of polymer chains to achieve a more thermodynamically stable nanomorphology.^29–31^ Nevertheless, only a limited number of new NFA-based polymers have been reported, and studies focusing on polymerization conditions and the degree of polymerization remain scarce.^32^ In this work, we investigate the synthesis and properties of a novel family of NFA-based polymers featuring an IDT core, *n*-phenyloctyl side chains, and δ-IC-Br flanking groups.

In this study, by employing different co-monomers, three materials, LuNi-2, LuNi-3, and LuNi-4, were synthesized. The polymerization conditions for the Stille cross-coupling reaction were first optimized using the 2,5-bis(trimethylstannyl)-thiophene co-monomer, and these optimal conditions were subsequently applied throughout the study. The influence of the co-monomers on the optoelectronic and structural properties of the pristine polymers, as well as on the photovoltaic performance and the thermal stability of the resulting devices, was systematically investigated.

## Results and discussion

### Synthesis of materials

**Scheme 1:**
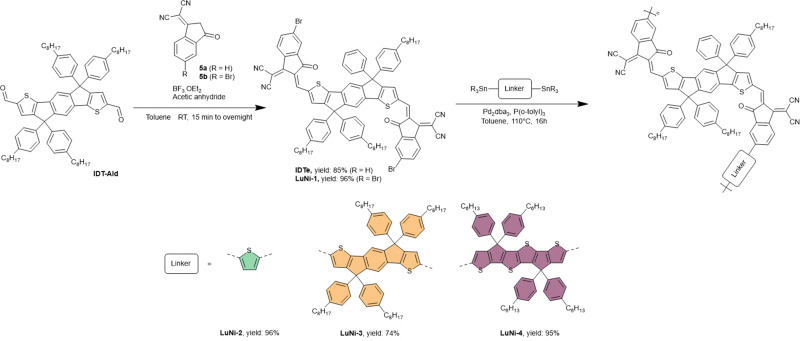
Synthetic pathways for LuNi-2, LuNi-3, and LuNi-4 *via* Stille polymerization.

The synthetic route for the monomer LuNi-1 and the optimization of the polymerization conditions used to produce LuNi-2 are presented in Scheme 1 and Table 1 respectively. LuNi-1 was synthesized in four steps, starting from diethyl 2,5-dibromoterephthalate and 2-(tributylstannyl)thiophene according to previous studies.^33,34^ Recently, Li's group reported a modified Knoevenagel condensation method employing an acid-catalyzed mechanism based on BF_3_·OEt_2_. This approach significantly accelerates the reaction and yields fewer impurities, thereby simplifying the purification of the final product.^35^

**Table 1:** LuNi-2 Polymer properties (*M*_n_, *M*_w_, *Đ*, yield, DP) as a function of palladium source and phosphine ligand

Experiment	Palladium source	Phosphine	*M*_n_ (kg mol^−1^)	*M*_w_ (kg mol^−1^)	*Đ*	Yield (%)	DP
1	Pd(PPh_3_)_4_	—	4.6	13.6	2.9	84	3.1
2	Pd_2_dba_3_	PPh_3_^a^	10.0	50	3.6	83	6.7
3	Pd_2_dba_3_	P(*o*-tolyl)_3_^a^	6.5	27.8	4.3	93	4.3
4	Pd_2_dba_3_	P(*t*-Bu)_3_^b^	1.9	2.5	1.3	55	1.3
5	Pd_2_dba_3_	PCy_3_^a^	8.0	26.4	3.3	97	5.3
6	Pd_2_dba_3_	dppf	5.4	11.7	2.1	96	3.6
7	Pd_2_dba_3_	SPhos	3.8	8.8	2.3	—	2.5

a Average.

b Degradation.

Several studies have highlighted that the choice of one of the isomers (related to the position of the Br atom) for the IC-Br flanking units significantly affects the polymer's conjugation and its photovoltaic performance.^36^ In this context, we isolated the desired δ-IC-Br isomer from the isomeric mixture *via* recrystallization in acetone. Finally, a BF_3_·OEt_2_-mediated Knoevenagel condensation was carried out using the purified isomer of 2-(5-bromo-3-oxo-2,3-dihydro-1*H*-inden-1-ylidene) malononitrile, affording LuNi-1 in excellent yield (96%) confirmed by ^1^H and ^13^C NMR (see Fig. S2–S27).

A brief survey of the literature highlights the importance of optimizing Stille polymerization conditions, as many studies rely solely on Pd(PPh_3_)_4_ as a catalyst and report highly variable molecular weights.^32^ A series of commercially available monodentate phosphine ligands, spanning a wide range of electronic richness and steric bulkiness, were selected to systematically investigate their influence on the oxidative addition and reductive elimination steps of the Stille polymerization process.^37,38^

To the best of our knowledge, no previous studies have systematically examined how different phosphine ligands affect the polymerisation of NFA-based monomers. The steric and electronic properties of phosphine ligands can significantly influence key steps of the catalytic cycle, so their selection is crucial for determining the yield and level of control over the polymerisation process.^37^ However, only a limited number of phosphine ligands have been tested to date, with Pd(PPh_3_)_4_ remaining the most commonly employed one in reported studies.^11,22,32,39^ Unfortunately, Pd(PPh_3_)_4_ suffers from limited air stability, as it readily oxidizes to its corresponding phosphine oxide, PPh_3_ = O. To address this issue, we conducted a comparative study using a series of mono- and bidentate phosphine ligands with varying cone angles and electron-donating properties (see Table S1). In all cases, Pd_2_(dba)_3_ was employed as the palladium source, owing to its greater air stability and versatility in ligand screening.^37^

All polymerization conditions, along with the corresponding number-average molecular weights (*M*_n_), weight-average molecular weights (*M*_w_), dispersity (*Đ*), yields, and degrees of polymerization (DP) prior to Soxhlet purification, are summarized in Table 1. The reaction performed using Pd(PPh_3_)_4_ (Experiment 1) served as the reference system. To assess reproducibility, polymerizations yielding the highest number-average molecular weights (*M*_n_), specifically those employing PPh_3_, P(*o*-tolyl)_3_, and PCy_3_, were repeated. The average values for each parameter across both trials are reported in Table 1, while the complete list of experimental conditions and results is available in Table S2.

Among the phosphine ligands, P(*t*-Bu)_3_ likely exhibited excessive reactivity, leading to the rapid degradation of the LuNi-1 monomer. In contrast, the use of dppf and SPhos ligand led to polymers with significantly lower *M*_n_, indicating reduced polymer growth efficiency. Switching the palladium source from Pd(PPh_3_)_4_ to Pd_2_(dba)_3_ while retaining PPh_3_ as the ligand (Experiment 2) yielded significantly different outcomes. Specifically, *M*_n_ values were substantially higher but more variable (6.0 and 14.0 kg mol^−1^, compared to 4.6 kg mol^−1^ with Pd(PPh_3_)_4_), while the yields remained comparable (79–87% *vs.* 84%).

The relatively low molecular weight obtained with Pd(PPh_3_)_4_ can be partly attributed to the excess free PPh_3_ present in solution. According to Farina *et al.*, this surplus phosphine inhibits the Stille coupling by deactivating the catalytic cycle and accelerating the formation of inactive palladium black, especially under oxidative conditions.^40^

In contrast, PCy_3_ demonstrated the ability to achieve relatively high number-average molecular weights (*M*_n_), although with some variability between experiments (6.1 and 9.9 kg mol^−1^). On the other hand, P(*o*-tolyl)_3_ yielded more consistent polymer sizes, with *M*_n_ values of 6.3 and 6.7 kg mol^−1^ across two trials. Notably, PCy_3_ also produced the most consistent dispersity indexes (*Đ* = 3.2 and 3.4), suggesting improved control over polymer growth. Overall, all three ligands, PPh_3_, P(*o*-tolyl)_3_, and PCy_3_, delivered high polymerization yields, averaging between 83% and 97%. Fig. S1 illustrates the correlation between the polymerization outcomes and two key parameters of the phosphine ligands: cone angle and Tolman's electronic parameter. With the exception of P(*t*-Bu)_3_, the results reveal only a weak correlation between the efficiency of the Stille polymerization and the electron-donating ability of the phosphine ligands. In this sense, utilising more electron-rich phosphine sources, as per Tolman's electronic parameter,^41^ such as P(*o*-tolyl)_3_ (Experiment 3) and PCy_3_ (Experiment 6)—tends to lower the molecular weight (*M*_n_) of the resulting polymers while increasing the polymerization yield. Additionally, electron-donating phosphines appear to correlate with a reduction dispersity. A closer examination of the influence of phosphine cone angle suggests a stronger correlation between polymerization outcomes and the steric bulk of the ligand, highlighting the significant role of steric hindrance in controlling the efficiency and precision of the Stille reaction.^42^ In our case, the results indicate that bulkier phosphine ligands tend to yield polymers with lower number-average molecular weights (*M*_n_) and narrower dispersity, while simultaneously enhancing the overall polymerization yield. These observations are consistent with previously reported mechanistic studies of the Stille reaction, which highlight the significant impact of the phosphine ligand's steric and electronic properties on the kinetics of the catalytic cycle. Specifically, while increased steric bulk is known to facilitate the reductive elimination step, it has also been shown that bulky and electron-rich phosphines can hinder the transmetallation step, thereby affecting the overall rate and control of the polymerization.^43^ Overall, the study highlights the strong influence of phosphine ligand structure on both the efficiency and control of Stille polymerization. While PPh_3_ is commonly considered an optimal ligand due to its balanced steric and electronic properties, PCy_3_ also performed well in our system, yielding polymers with molecular weights approaching 10.0 kg mol^−1^. Although P(*o*-tolyl)_3_ did not deliver the highest molecular weights, it provided the best overall compromise between efficiency and reproducibility, consistently affording high yields and uniform polymer sizes across multiple experiments. Moreover, since the molecular weight of the acceptor polymer has not been identified as the most critical factor for the long-term stability of organic solar cells (OSCs),^27^ P(*o*-tolyl)_3_ was chosen as the ligand for the synthesis of LuNi-3 and LuNi-4. Ongoing studies aim to further elucidate the role of phosphine ligands in influencing the reaction mechanism and the resulting polymer properties.

In order to investigate the impact of co-monomers on the optical absorption properties of the materials, LuNi-1 was copolymerised with two different fused-ring units. LuNi-3 incorporates a co-monomer structurally analogous to the central core of LuNi-1 (IDT), while LuNi-4 was synthesised using a modified IDT unit featuring a more electron-rich thieno-thiophene-based central core (Scheme 1 Synthetic routes of (a) the NFA monomer LuNi-1 and (b) the optimisation of the Stille polymerization). These co-monomers are expected to narrow the bandgap and promote planarity of the molecular backbone, while preventing over-aggregation thanks to their out-of-plane *n*-phenyloctyl side chains.^44,45^ The three materials were synthesized under the previously optimized conditions using the Pd_2_(dba)_3_/P(*o*-tolyl)_3_ catalytic system. Full synthetic procedures are provided in the SI. The resulting copolymers were purified by precipitation in methanol, followed by sequential Soxhlet extraction with hexane, dichloromethane, chloroform, and chlorobenzene. The final yields were 79%, 74%, and 95% for LuNi-2, LuNi-3, and LuNi-4, respectively. The polymers exhibit good solubility in common organic solvents such as chloroform, chlorobenzene, and *o*-xylene. Their molecular weights were determined by size exclusion chromatography (SEC) performed at 30 °C using chloroform as the eluent and polystyrene standards for calibration (Table 2 and Fig. S28). The number-average molecular weights (*M*_n_) of LuNi-2, LuNi-3, and LuNi-4 were found to be 12.7 kg mol^−1^, 5.8 kg mol^−1^, and 19.3 kg mol^−1^, respectively, with corresponding dispersity (*Đ*) values of 2.5, 2.1, and 3.0.

**Table 2:** Yields, molecular weights, dispersity indexes, optical and electrochemical properties of LuNi-2, LuNi-3 and LuNi-4

Polymer	Yields (%)	*M*_n_ (kg mol^−1^)	*M*_w_ (kg mol^−1^)	*Đ*	*λ*_onset_ (nm)	*E* ^opt^_g_ (eV)	*E*_HOMO_ (eV)	*E*_LUMO_ (eV)	*E* ^elec^_g_ (eV)
LuNi-2	79	12.7	31.4	2.5	747	1.66	−5.8	−4.0	1.8
LuNi-3	74	9.6	23.2	2.4	745	1.66	−5.6	−3.9	1.7
LuNi-4	95	19.3	57.7	3.0	834	1.49	−5.3	−3.9	1.4

### Optoelectronic properties

**Fig. 1:**
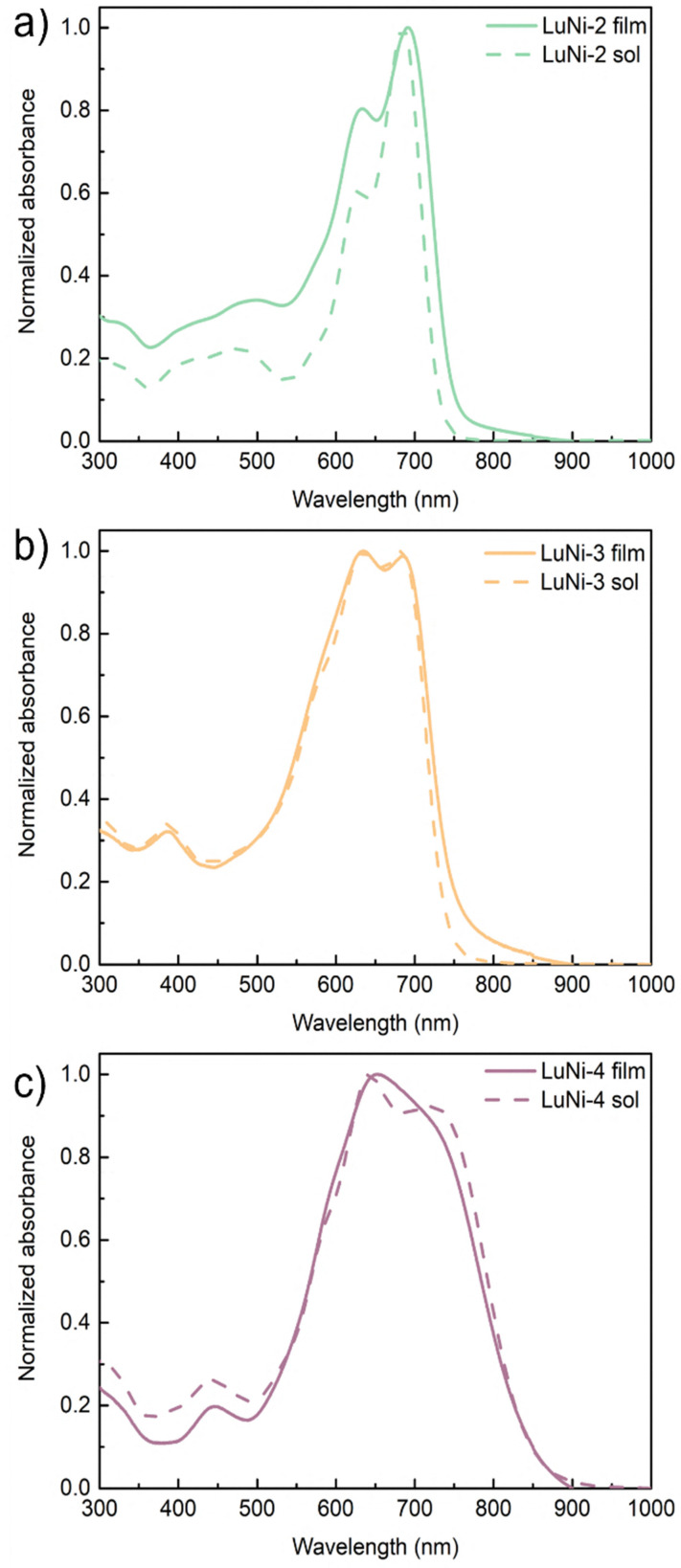
UV-Visible spectra in solution and in thin film of (a) LuNi-2, (b) LuNi-3 and (c) LuNi-4.

**Fig. 2:**
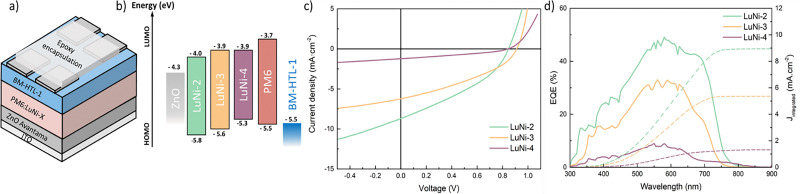
(a) Scheme of an inverted device, (b) energy diagram of LuNi-2, LuNi-3, LuNi-4 thin film compared to ZnO, BM-HTL-1 and PM6 materials, (c) *J–V* curves and (d) EQE curves of the best performing pixels.

Thin-film cyclic voltammetry (CV) measurements were conducted to evaluate the energy level alignment of polymers in relation to potential donor materials, such as PM6. The HOMO and LUMO energy levels were estimated from the oxidation and reduction onset potentials, respectively, and calibrated against the ferrocene/ferrocenium (Fc/Fc^+^) redox couple (see Fig. S29–S31 for details).^46^ The results are summarized in Table 2. The HOMO and LUMO energy levels were estimated to be −5.8 eV and −4.0 eV for LuNi-2, −5.6 eV and –3.9 eV for LuNi-3, and –5.3 eV and –3.9 eV for LuNi-4, respectively. The corresponding electrochemical bandgap values (*E*^elec^_g_) were calculated to be 1.74 eV for LuNi-2, 1.66 eV for LuNi-3 and 1.39 eV for LuNi-4 (Fig. 1). These values align well with the optical band gaps determined by UV-vis spectroscopy and the theoretical predictions from DFT calculations (see Fig. S35). Additionally, the LUMO levels of these polymers are approximately −3.9 eV, which is close to that of typical small-molecule NFAs and favourable for electron injection from the LUMO level of the donor material.^47^ However, the HOMO level of LuNi-4 (5.3 eV) is higher than that of the PM6 donor material (5.5 eV), while that of LuNi-3 is quite similar (5.6 eV). Accurately predicting energy level alignment remains challenging, particularly as the HOMO level can vary depending on molecular orientation and interfacial effects within the complete device architecture. In our case, the measured energy levels suggest a potential mismatch with PM6, which could lead to suboptimal energy level alignment and reduced device efficiency (Fig. 2).^48^ To gain a deeper insight into the electronic structure and charge distribution, theoretical calculations were performed using the B3LYP/def2-TZVP method. To improve computational efficiency, each polymer was represented by a single repeat unit and the long alkyl side chains were replaced with methyl groups. The resulting electron density distributions of the HOMO and LUMO orbitals, based on the optimised geometries, are shown below in Fig. S32–S34. For LuNi-2, the HOMO–LUMO overlap was localized exclusively on the NFA core, with no significant delocalization through the thiophene linker (Fig. S32). These findings indicate that, in this case, the thiophene unit functions primarily as a structural spacer, contributing minimally to the electronic delocalization along the conjugated polymer backbone. By contrast, the other two polymers exhibit significant differences in the localisation of their HOMO and LUMO, which extend from the NFA core to the comonomer units (see Fig. S33 and S34). However, the torsion angle between the different monomers (see Fig. S36) remains rather low and few differences are observed between these three polymers by DFT calculation (respectively 0°, 5° and 6° for LuNi-2, LuNi-3 and LuNi-4). This suggests a greater degree of electronic delocalisation along the polymer backbone for LuNi-3 and LuNi-4, a characteristic commonly found in traditional donor–acceptor copolymer structures.

UV-Vis absorption spectroscopy was used to study the optical properties of the three polymers in solution and as thin films (see Fig. 1 and Fig. S37). The corresponding optoelectronic parameters are summarised in Table 2. The absorption spectrum of LuNi-2 is quite similar to that of the LuNi-1 monomer, but shows a slight red shift in both the absorption maximum and the onset of the band. Incorporating a thiophene spacer unit results in a modest increase in absorption intensity within the 400–500 nm range. For LuNi-3, introducing a more extended conjugated unit only causes a minor shift in the absorption maximum. However, the absorption band significantly broadens, extending across the 400–650 nm region, which confirms improved light-harvesting capabilities. LuNi-4 features the most conjugated comonomer and its absorption spectrum is further broadened and red-shifted. The absorption edge extends well into the near-infrared region, beyond 850 nm.

In the solid state, all three polymers exhibit a red shift in the absorption maximum ranging from 13 to 15 nm, accompanied by notable broadening of the absorption bands. The absence of pronounced vibronic features indicates a low level of molecular organisation or aggregation in the solid state.^49^ The optical bandgaps (*E*^opt^_g_) estimated from the absorption edges in thin films are 1.66 eV for LuNi-2, 1.66 eV for LuNi-3, and 1.49 eV for LuNi-4 (Table 2).

These results are in good agreement with the cyclic voltammetry (CV) measurements, confirming the similar optical band gaps of LuNi-2 and LuNi-3, as well as the significantly lower band gap of LuNi-4. This reduced band gap is attributed to the presence of an electron-rich co-monomer in LuNi-4, which raises the HOMO energy level relative to the other two polymers.

To further investigate the aggregation behavior of these polymers in solution, temperature-dependent UV-vis absorption measurements were conducted in *o*-xylene at a low concentration of 0.05 mg mL^−1^ (see Fig. S38). Upon increasing the temperature, the absorption spectrum of LuNi-2 exhibits a slight blue shift, but the aggregation-related peak (associated with the 0–1 transition) does not show a clear decrease, indicating relatively weak or thermally stable aggregation under these conditions.

### Structural organisation and film morphology of polymers

To investigate the molecular organization of the pristine materials in thin films, grazing incidence wide-angle X-ray scattering (GIWAXS) measurements were performed in both in-plane (IP) and out-of-plane (OOP) geometries (Fig. S39 and S40). All three polymers exhibit a quasi-amorphous morphology, indicative of a low degree of structural order. In contrast to the structurally related PZ1 polymer,^11^ the characteristic π–π stacking (010) reflection at 3.92 Å and the lamellar (100) peak at 27.92 Å are absent in the GIWAXS patterns of LuNi-2, LuNi-3, and LuNi-4, suggesting a lack of long-range crystalline order. This observation corroborates the UV-vis analysis that suggested a low ordering in the solid state.

The surface morphology of the polymer thin films was further examined by atomic force microscopy (AFM) in peak force mode. The corresponding topographic images and calculated root-mean-square (RMS) roughness values are provided in the SI (Fig. S41). AFM analysis reveals that the surfaces of all three polymers are relatively featureless, with low roughness values of approximately 2 nm. However, in agreement with the GIWAXS data, LuNi-2 displays slightly more surface structuring compared to LuNi-3 and LuNi-4, indicating a marginally higher degree of molecular organization at the surface.

In comparison to PZ1, one hypothesis is that the out-of-plane phenyl groups on the IDT core increase steric hindrance, thereby hindering crystallization and limiting the suitability of this specific core for highly ordered polymer assemblies. However, LuNi-1 demonstrates a fibrillar morphology, which are characteristic of this class of materials (see Fig. S41), suggesting that the structure of the core itself does not fully prevent ordering. An alternative explanation is that the use of more elongated co-monomers, as in LuNi-4, disrupts backbone planarity and linearity, making it more challenging to achieve tight interchain packing and ordered domains.^19^ It is also suspected that the loss of crystallinity is due to the addition of a *para*-phenyl-octyl side chain to the spiro carbon. This lengthens the side chains and increases the distance between the π-conjugated backbones.^50,51^

### Fabrication of solar cells for stability assessment

One of the goals of this study was to demonstrate that an all-polymer active layer can offer high thermal stability. To evaluate this in real operation conditions, we fabricated devices using the configuration shown in Fig. 2. Due to the amorphous nature of the synthesized materials, which inherently limits efficiency compared to state-of-the-art systems, we did not aim for full performance optimization in these devices. To evaluate the photovoltaic performance and stability of blends based on LuNi-2, LuNi-3, and LuNi-4, inverted bulk heterojunction solar cells were fabricated with the structure ITO/ZnO/active layer/BM-HTL/Ag.^52^ The polymers were blended with the benchmark donor polymer PM6 in a 1 : 1.5 ratio using *o*-xylene as processing non-chlorinated solvent. The completed devices were encapsulated with epoxy resin (see SI for further details). Table S3 summarizes the key photovoltaic parameters: open-circuit voltage (*V*_OC_), short-circuit current density (*J*_SC_), fill factor (FF), and power conversion efficiency (PCE). Fig. 2 presents the *J*–*V* characteristics of the devices measured under AM1.5G illumination, along with the external quantum efficiency (EQE) spectra of the best-performing device for each polymer.

The PCE values extracted from the *J*–*V* curves show that LuNi-2 is the most efficient material, with a *V*_OC_ of 0.85 V, a *J*_SC_ of 8.69 mA cm^−2^ and a fill factor of 36%. LuNi-2's current density surpasses that of LuNi-3 and LuNi-4 (6.22 and 1.23 mA·cm^−2^, respectively), consistent with trends observed in the IPCE spectra. LuNi-4's poor performance (PCE = 0.34%) can be attributed to its high HOMO energy level, which is poorly aligned with that of PM6. Although LuNi-2 and LuNi-3 have similar LUMO energy levels, the LuNi-3-based device has a slightly higher *V*_OC_ (0.91 V) than the LuNi-2 device (0.85 V). This suggests that the LuNi-2-based device may experience greater voltage losses, which are likely to arise from enhanced non-radiative recombination. This phenomenon is a key factor influencing the performance of bulk heterojunction solar cells and can be investigated through light-intensity-dependent measurements^53–55^ or impedance spectroscopy. Thus, to better understand the *V*_OC_ difference between LuNi-2 and LuNi-3, light-intensity-dependent *J*–*V* characteristics and impedance spectroscopy were measured for PM6 blended with these different polymers. The prevailing charge recombination mechanism, whether monomolecular or bimolecular, can be elucidated by analysing the slope of the open-circuit voltage (*V*_OC_) as a function of the logarithm of the incident light intensity (ln *I*), in order to determine the recombination order. Fig. S43a shows the dependence of *V*_OC_ on light intensity for devices based on the PM6:LuNi-2 and PM6:LuNi-3 blend systems. A slope of 2*kT*/*q*, where *k* is the Boltzmann constant, *T* is the absolute temperature and *q* is the elementary charge, is typically indicative of monomolecular (trap-assisted) recombination. In contrast, a slope approaching *kT*/*q* suggests that bimolecular recombination is the dominant loss mechanism.^56^ Under open-circuit conditions, the extracted slope for LuNi-2 (1.12 *kT*/*q*) is lower than that for LuNi-3 (1.31 *kT*/*q*), implying that bimolecular recombination contributes slightly more to the loss mechanism in the LuNi-2-based device. Higher slope values generally indicate that monomolecular recombination dominates the charge loss processes. In the case of LuNi-2, the lower *V*_OC_ combined with the higher *J*_SC_ suggests more pronounced overall charge losses due to bimolecular recombination, particularly at elevated carrier densities. This is also reflected in the EQE measurements. To investigate the recombination dynamics further, impedance spectroscopy was conducted under *V*_OC_ conditions and at varying light intensities for PM6:LuNi-2 and PM6:LuNi-3 devices (see Fig. S42). The results show that LuNi-2 exhibits a shorter recombination lifetime than LuNi-3, indicating faster recombination kinetics. This is consistent with the lower *V*_OC_ observed for LuNi-2, despite the two materials having relatively similar optical band gaps. These findings also align with the analysis under short-circuit conditions as a function of light intensity. The relationship between the steady-state short-circuit current density (*J*_SC_) and the incident light intensity (*I*) typically follows a power-law dependence of the form *J*_SC_ ∝ *I*^*α*^ (Fig. S43b and S44). The exponent *α* serves as an indicator of the dominant recombination mechanism: a value of *α* close to 1 suggests that first-order (monomolecular) recombination processes dominate, whereas an α value approaching 0.5 is indicative of significant second-order (bimolecular) recombination.^56,57^LuNi-2 (0.918) and LuNi-3 (0.917) exhibit similar values, indicating that the recombination is predominantly monomolecular. Conversely, the *V*_OC_ behaviour at various light intensities suggests that the recombination is primarily bimolecular. This discrepancy has already been discussed in literature^58,59^ and is due to the fact that the devices are limited by electronic transport rather than by recombination. This is also consistent with the high *V*_OC_ levels achieved despite the good absorption properties and the low *J*_SC_ observed.

To complement the light-intensity dependent analysis and gain further insight into the excited-state dynamics, steady-state and time resolved photoluminescence measurements were performed on pristine polymers and their blends with PM6 (Fig. S45–S48 and Table S4).^60–62^LuNi-2 exhibits a slightly smaller Stokes shift than LuNi-3, consistent with a more rigid excited-state structure induced by the thiophene spacer. The PM6:LuNi-2 blend exhibits stronger PL quenching (82%) and shorter exciton lifetimes (0.81 ns) compared to PM6:LuNi-3 (48% quenching, 1.05 ns), indicating more efficient exciton dissociation but faster bimolecular recombination. This behaviour is consistent with the shorter recombination lifetimes observed by impedance spectroscopy and explains the higher *J*_SC_ and the lower *V*_OC_ of LuNi-2-based devices.

### Thermal and photostability

**Fig. 3:**
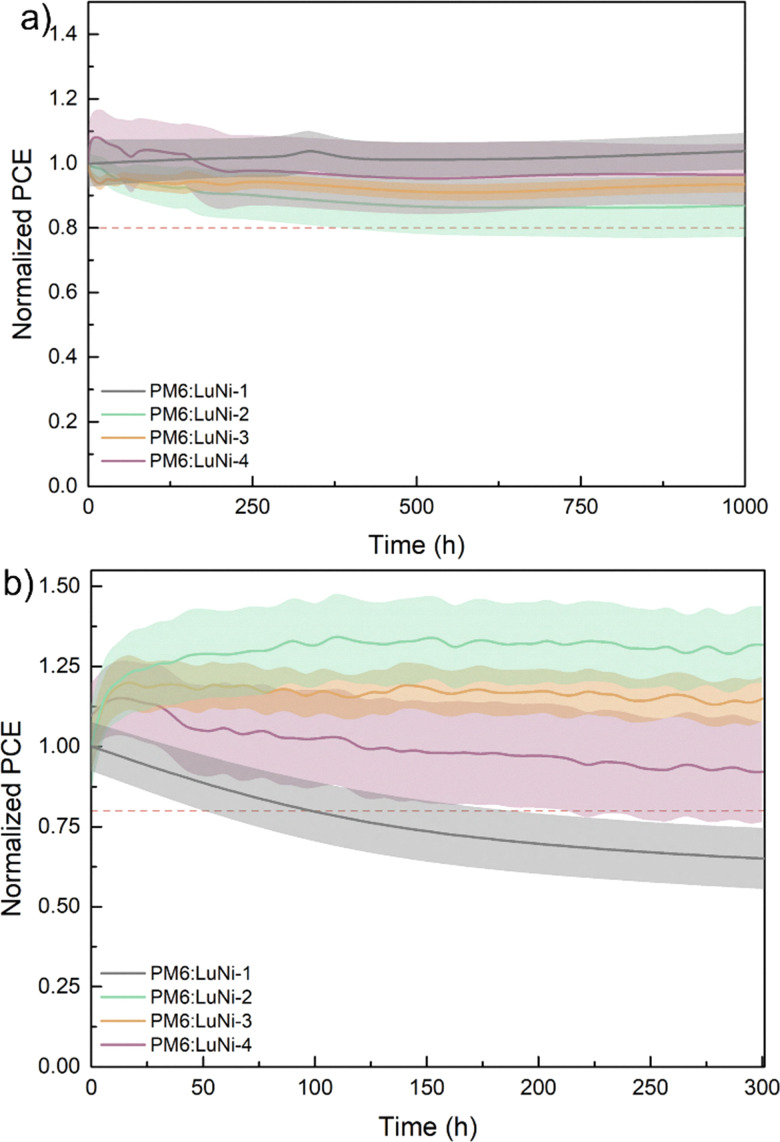
Variation of normalized PCE of the relevant devices. (a) Thermal stability of the devices based on PM6:LuNi-1, PM6:LuNi-2, PM6:LuNi-3, and PM6:LuNi-4 baked in an oven at 65 °C, (b) photostability devices based on PM6:LuNi-1, PM6:LuNi-2, PM6:LuNi-3, and PM6:LuNi-4 measured under one sun illumination in ambient humidity at room temperature (The continuous line represents the average and the colored bands represent the standard deviation of 8 pixels).

Before studying the thermal stability of the active layer in complete devices, it is important to study the thermal properties of LuNi-2, LuNi-3 and LuNi-4 in their pure state, and to do this we used thermogravimetric analysis (TGA). Thermogravimetric analysis (TGA) was performed under a nitrogen atmosphere at a heating rate of 10 °C per minute. The polymers exhibited excellent thermal stability, with no significant degradation observed up to 426 °C for LuNi-2 and LuNi-4 and 415 °C for LuNi-3. This confirms their robustness under thermal stress (see Fig. S49). To assess long-term thermal stability further, all-polymer solar cells encapsulated with PM6:LuNi-1, PM6:LuNi-2, PM6:LuNi-3 and PM6:LuNi-4 were subjected to thermal ageing in accordance with the ISOS-D-2 protocol: stored in the dark at 65 °C for 1000 hours. After 1000 hours of continuous thermal ageing, the PM6:LuNi-2, PM6:LuNi-3 and PM6:LuNi-4 devices all retained between 90% and 100% of their initial PCE and demonstrated stable performance. Notably, negligible burn-in effects were observed over the course of the experiment. (see Fig. 3a and Fig. S50–S52 for all photovoltaic parameters).

Demonstrating strong photochemical stability under prolonged polychromatic illumination is also essential when designing new materials for photovoltaic applications. Consequently, the photostability of the same devices was evaluated under continuous illumination at one sun intensity with UV-filter, in accordance with the ISOS-L-1 protocol (see Fig. 3b and Fig. S53–S55 for all photovoltaic parameters). An improvement in performance is observed under illumination over the first 50 hours. This is due to the progressive activation of the zinc oxide (ZnO)-based electron transport layer (ETL) with UV filter.^52,63^ Interestingly, all-polymer solar cells (APSCs) outperformed the PM6:LuNi-1 bulk heterojunction device, which showed a significant decline in performance after just six hours of illumination. In contrast, the APSCs maintained stable operation for over 300 hours. Devices incorporating LuNi-2 as the acceptor demonstrated exceptional thermal and photochemical stability, thereby strengthening the approach of developing NFA-based polymers for achieving high thermal and photostability in organic solar cells.

## Conclusions

In this study, we synthesized three new polymers based on non-fullerene acceptors, each incorporating a different co-monomer. We systematically investigated the effect of the phosphine ligand structure on the reaction yield and control of Stille polymerisation. Of the various conditions tested, using tris(*o*-tolyl) phosphine (P(*o*-tolyl)_3_) alongside Pd_2_(dba)_3_ as the palladium source produced the best balance of reactivity and control. This yielded polymers with high molecular weights and good yields. All three polymers exhibited broad UV-vis absorption and medium optical band gaps ranging from 1.5 to 1.7 eV, with HOMO energy levels between −5.3 and −5.6 eV and LUMO levels between −3.9 and −4.0 eV. Structural characterisation of the polymers in thin films revealed that they are all amorphous. To evaluate their thermal and photochemical robustness, all-polymer solar cells (APSCs) were fabricated using these materials as acceptors and PM6 as the donor. Device performance remained modest (below 2.7%), primarily due to the amorphous nature of the polymers, which limits charge transport. Nevertheless, all three systems exhibited excellent thermal and photochemical stability. The devices retained between 90% and 100% of their initial power conversion efficiency (PCE) after 1000 hours at 65 °C and exhibited less than a 10% efficiency loss after 300 hours of continuous one-sun illumination.

The stability of most reported devices is widely distributed, with the majority retaining 70–90% of their initial efficiency within the first 500–1000 hours. By contrast, our materials demonstrate superior stability, retaining over 90% of their PCE after 1000 hours of thermal aging (Fig. S56 and Table S5, S6).

Y6-based devices exhibit a wide variety of photostability outcomes, with many experiencing significant degradation within the first 1000 hours. Our materials however exhibit excellent stability, maintaining ∼95% of their initial efficiency after 300 hours of continuous illumination. This stability demonstrates the effectiveness of the all-polymer strategy in improving long-term operational stability of organic solar cells.

This work emphasises the importance of identifying appropriate polymerisation conditions for producing new family of polymeric materials, allowing to develop more robust active layers in terms of both thermal and photochemical properties.

## Author contributions

L. R., C. J. and S. F. synthesized and characterized the monomers and the polymers. L. R. performed the DFT calculations. L. R. fabricated the devices and L. R., A. C. and A. J. R. characterized them. A. J. R. and L.R. performed impedance spectroscopy and analysed the data. R. D. analysed the data and edited the manuscript and C. A. supervised the work, designed the experiments, analysed the data, wrote and edited the manuscript. C. A. acquired the funding. All the authors contributed to the work and gave approval to the manuscript before submission.

## Conflicts of interest

There are no conflicts to declare.

## Supplementary Material

TC-013-D5TC02570B-s001

## Data Availability

All data supporting the findings of this study are provided in the supplementary information (SI). Supplementary information: this includes theoretical calculations, synthetic procedures, attributed and ^1^H and ^13^C NMR spectra of intermediates and final compounds, optical measurements, cyclic voltammetry curves, and details regarding the preparation and characterization of the devices and the thermal and light soaking stability measurements. See DOI: https://doi.org/10.1039/d5tc02570b. The raw data supporting the conclusions of this article are available from the corresponding authors upon reasonable request.
