# Supplementary material for: Synthesis and characterization of NFA-based polymers for solar cells with improved thermal stability
Source: J Mater Chem C Mater. 2025 Sep 25;13(42):21357–66. doi: 10.1039/d5tc02570b (PMC12461689; doi:10.1039/d5tc02570b)
Supplement: TC-013-D5TC02570B-s001 [file TC-013-D5TC02570B-s001.pdf]

Supplementary Information (SI) for Journal of Materials Chemistry C.
This journal is © The Royal Society of Chemistry 2025

Supporting Information

Synthesis and characterization of NFA-based polymers for solar cells with improved thermal stability

Lucie Rivet[a], Antoine Curé [a], Camille Jutard [a], Samuel Fauvel [a], Renaud Demadrille [a], Antonio J. Riquelme[a] and Cyril Aumaître*[a]

[a] IRIG-SyMMES, Université Grenoble Alpes/CEA/CNRS/Grenoble INP, 38000 Grenoble, France.
E-mail : cyril.aumaitre@cea.fr

GENERAL METHODS 2
- Materials. 2
- Electrochemical characterization. 2
- Theoretical calculations. 2
- Thin film preparation and characterization 3
- Solar cells fabrication. 3
- Photovoltaic characterization. 3

SYNTHETIC ROUTES 4
- Synthesis of compound 3 (Scheme S2) 4
- Synthesis of compound 5 (Scheme S1) 5
- Synthesis of IDTe and LuNi-1 (Scheme S3) 6
- Synthesis of compound 6 (Scheme S4) 7
- Synthesis of compound 10 (Scheme S5) 7
- Synthesis of polymers (LuNi-2, LuNi-3 and LuNi-4) (Scheme S6) 9

STUDY OF THE DIFFERENT PHOSPHINE FOR THE POLYMERIZATION 11
NMR SPECTRA 13
SIZE-EXCLUSION CHROMATOGRAPHY 27
CYCLIC VOLTAMMETRY 28
DFT CALCULATIONS 29
UV-Vis SPECTROSCOPY 31
GRAZING INCIDENCE WIDE ANGLE X-RAY SCATTERING 32
PEAK FORCE MODE ATOMIC FORCE MICROSCOPY 33
PHOTOVOLTAIC PERFORMANCES 34
IMPEDANCE SPECTROSCOPY 34
RECOMBINATION KINETICS 35
PHOTOLUMINESCENCE 36
THERMAL GRAVIMETRIC ANALYSIS 38
THERMAL STABILITY 38
UNDER ILLUMINATION STABILITY 41

LITERATURE REVIEW 44
REFERENCES 47

GENERAL METHODS

Materials.

All chemical reactions were performed using oven-dried glassware under an argon atmosphere. Anhydrous solvents, including toluene, chloroform, and acetonitrile (purchased from Aldrich or Acros Organics), were used without further purification. Tetrahydrofuran (THF) was freshly distilled over sodium/benzophenone prior to use. Starting materials were obtained from Sigma-Aldrich, Acros Organics, or TCI Chemicals and used as received. *o*-xylene and chlorobenzene (CB) were purchased from Sigma-Aldrich. HTL layer was purchased from Brilliant Matters (BM-HTL-1). BM-HTL-1 is a fully printable, hole-transporting layer (HTL)with a high work function. It enables us to create a fully spin-coatable stack prior to evaporating the metallic counter electrode. Other studies have already employed this solution to speed up the deposition process[1,2].

Electrochemical characterization.

Cyclic voltammograms (CVs) were recorded on a Solartron 1287 potentiostat using platinum electrodes at a scan rate of 50 mV.s^{-1} and a Ag/Ag^{+} (0.01 M of AgNO3 in elecetrolyte) reference electrode in an anhydrous and argon-saturated solution of 0.1 M of tetrabutylammonium tetrafluoborate (Bu_4NBF_4) in acetonitrile. Tetrabutylammonium tetrafluoborate (98%, Sigma Aldrich) was recrystallized from a methanol/water mixture and dried at 70°C under reduced pressure.[3] In these conditions, for a freshly prepared reference electrode, the half-wave oxidation potential ($E^{½}$) of ferrocene should be around 0.091 V versus Ag/Ag+ as reported by Li et al.[4] The HOMO and LUMO energy levels were determined from the oxidation and reduction onsets and by assuming the energy level of ferrocene/ferrocenium (Fc/Fc^{+}) to be -4.8 eV below the vacuum level.[5–7] The onsets potential (E') was determined at the point at which the current began to differ from the baseline.

$$E_{HOMO}(eV) = -[(E^{'}_{ox\ vs\ Ag/Ag^{+}}) - (E^{1/2}_{Ferrocene\ vs\ Ag/Ag^{+}}) + 4.8$$

$$E_{LUMO}(eV) = -[(E^{'}_{red\ vs\ Ag/Ag^{+}}) - (E^{1/2}_{Ferrocene\ vs\ Ag/Ag^{+}}) + 4.8$$

$$E_{LUMO} = E_{HOMO} + E_{g}$$

Theoretical calculations.

Geometry optimizations were performed using the ORCA computational package with the PBE functional and the def2-svp basis set, corrected for improved structural optimization. Orbital simulations were conducted via single-point energy calculations employing the B3LYP hybrid functional on the previously optimized geometries and the def2-tzvp basis set. To reduce computational cost, only a single repeating unit of each polymer was considered, and long alkyl side chains were replaced with methyl groups.

Thin film preparation and characterization

To prepare polymers films, 12 mg/mL of the polymer acceptor was dissolved in chloroform and stirred overnight at 60 °C. The polymer solution was spin coated on top of cleaned glass at different speed and then annealed for 10 min at 110 °C. UV–visible spectra were obtained on a Agilent Technologies Cary 60 spectrophotometer. The absorbance of the samples was measured and integrated in the 300-900 nm range at different temperatures. The atomic force microscopy (AFM) images were recorded with a Dimension Icon Bruker AFM in peak force tapping mode with ScanAsyst-Air tips (NanoScope software version 9.40). All AFM experiments were carried out under ambient conditions. The images were treated using the WSxM software (version 5.0 Develop 9.1). Thin films were further analyzed by grazing incidence wide-angle-X-ray scattering (GIWAXS) using a Rigaku SmartLab setup. The incident angle and wavelength of X-rays were 0.18 ° and 0.154 nm respectively.

Solar cells fabrication.

PM6:**LuNi-2** or **LuNi-3** or **LuNi-4** inks were prepared in o-xylene in a total concentration of 20 mg/mL in ratio 1:1.5 and stirred at 65 °C overnight. ITO-coated glass(sheet resistance 15 Ω/square) purchased from Visiontek were sequentially cleaned with deionized water, acetone and isopropanol under sonication for 15 minutes each, dried with argon and then treated in a UV-ozone oven for 30 minutes at 80 °C. All the devices were fabricated in inverted structure ITO/ZnO/Active Layer/HTL-BM/Ag. ZnO nanoparticles (purchased from Avantama) layer was spin-coated in air from at 4000 rpm follow by thermal annealing at 120°C for 10 minutes. Then the blend solution (at 65 °C) was dynamically spin coated at 2000 or 4000 or 6000 rpm during 40 seconds on top of ZnO films. The contact surface was cleaned with DCM. The BM-HTL-1 was spin coated at 3000 rpm during 20 seconds on the top of the blend previously heated to 65°C. The contact surface was then cleaned with acetone before a thermal annealing at 120 °C for 10 minutes. Finally, 100 nm of silver was deposited by thermal evaporation through a mask to define an active area of 0.0256 cm^2.

Photovoltaic characterization.

The current density-voltage characterization of the devices under dark and under AM 1.5G irradiation condition (1000 W·m^{-2}) using a OSSILA measurement platform coupled to an AM 1.5G solar simulator (Newport class AAA). It was calibrated prior to the measurements using a reference silicon photodiode. This reference photodiode consisted of a readout device and a 2 x 2 cm calibrated monocrystalline silicon solar cell. The cell was also equipped with a thermocouple assembled in accordance with IEC 60904-2. The certification is accredited by the National Institute of Standards and Technology to the ISO-17025 standard and is traceable to the National Renewable Energy Laboratory. The EQE of the solar cells was measured using a monochromator Newport led by Arkeo Cicci Research instrument. The electrical impedance spectroscopy (EIS) measurements were done using an Autolab PGSTAT30 FRA2 potentiostat controlled by the NOVA 2.1.7 software. The studies were conducted on complete solar cells under white light illumination using Thorlabs LED in a wide range of DC light intensities. The impedance spectra were measured using the open circuit voltage attained at each illumination as the applied bias potential and applying a sinusoidal voltage perturbation of 10 mV. The thermal stability in the dark of the devices were measured using a LED-based solar simulator (Ossila). The stability under illumination of the devices were measured using a AM1.5G light solar simulator (Lumartix Solixon A-22).

SYNTHETIC ROUTES

Synthesis of compound 3 (Scheme S2)

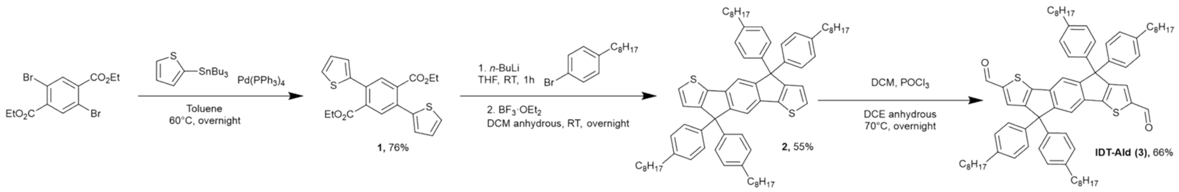

<u>Diethyl 2,5-di(thiophen-2-yl)terephthalate (1).</u> Diethyl 2,5-dibromoterephthalate (5.76 g, 15.2 mmol) and tetrakis(triphenylphosphine) palladium(0) (438 mg, 0.38 mmol) were dried under vacuum for 15 minutes before being dissolved in toluene (80 mL) under argon atmosphere. The reaction mixture was bubbled with argon for another 15 minutes before adding 2-(Tributylstannyl)thiophene (10.6 mL, 33.34 mmol) and then stirred at 80 °C overnight. After a night, 2-3 spoons of activated carbon were added to the mixture. The mixture was filtered on celite and then extracted with ether, washed three times with a saturated ammonium chloride solution, dried over Na_2SO_4 and concentrated under vacuum. The crude product was recrystallised in cold pentane to give the final product (4.42 g, 76 %). ^{1}H NMR (400 MHz, $CDCl_3$): δ 7.81 (s, 2H), 7.39 (dd, *J* = 1.43, 4.87 Hz, 2H), 7.08 (m, 4H), 4.22 (q, *J* 7.14 Hz, 4H), 1.15 (t, *J* = 7.14 Hz, 6H). ^{13}C NMR (400 MHz, $CDCl_3$): 167.70, 140.50, 134.08, 133.45, 131.88, 127.35, 126.98, 126.48, 61.66, 13.81. Anal. Calcd for $C_{20}H_{18}O_4S_2$: C, 62.16; H, 4.69; S, 16.59. Found: C, 60.73; H, 4.48; S, 16.37.

<u>4,4,9,9-tetrakis(4-octylphenyl)-4,9-dihydro-s-indaceno[1,2-b:5,6-b'] dithiophene (2).</u> Under argon atmosphere, n-BuLi (5.69 mL, 2.5 M, 14.23 mmol) was added dropwise to a solution of 1-bromo-4-octylbenzene (3.80 mL, 15.52 mmol) in THF (180 mL) cooled at -78 °C. The reaction mixture was stirred at -78 °C during 30 minutes before the dropwise addition of a solution of compound **1** (1 g, 2.59 mmol) in THF (10 mL) previously bubbled with argon for 30 minutes. Then the reaction mixture was allowed to warm to room temperature and stirred overnight. The reaction was quenched with 40 mL of a saturated baking soda solution and 160 mL of water. The reaction mixture was stirred during 20 minutes. The organic phase was extracted with ether, washed with water and brine, dried over Na_2SO_4 and concentrated under vacuum. The crude product (yellow oil) is used in the next reaction without further purification. Under argon, a solution of the crude product (2.73 g, 2.59 mmol) in anhydrous DCM (200 mL) was cooled at 0 °C before adding boron trifluoride etherate (6.6 mL, 53.27 mmol) dropwise. Then the reaction mixture was allowed to warm to room temperature and stirred overnight. The reaction was quenched with 50 mL of a saturated baking soda solution and then washed with water. The organic phase was dried over Na_2SO_4 and concentrated under vacuum. The crude was purified by silica gel chromatography using hexane:dichloromethane (10:1) as eluent to obtain a yellow solid (1.44 g, 55 %). ^{1}H NMR (400 MHz, $CDCl_3$): δ 7.44 (s, 2H), 7.24 (d, *J* = 4.91 Hz, 2H), 7.16 (d, *J* = 8.33 Hz, 8H), 7.05 (d, *J* = 8.37 Hz, 8H), 7.00 (d, *J* = 4.90 Hz, 2H), 2.56 (t, *J* = 7.80 Hz, 8H), 1.59 (m, 8H), 1.29 (m, 40H), 0.88 (t, *J* =6.62 Hz, 12H). ^{13}C NMR (400 MHz, $CDCl_3$): 155.89, 153.44, 142.10, 141.38, 141.30, 135.13, 12830, 127.92, 127.40, 123.15, 117.51, 62.69, 35.60, 31.93, 31.41, 29.51, 29.28, 22.71, 14.15. Anal. Calcd for $C_{72}H_{90}S_2$: C, 84.81; H, 8.90; S, 6.29. Found: C, 83.36; H, 8.55; S, 6.19.

4,4,9,9-tetrakis(4-octylphenyl)-4,9-dihydro-s-indaceno[1,2-b:5,6-b']dithiophene-2,7-dicarbaldehyde IDT-Ald (3). Phosphoryl chloride (0.5 mL, 5.35 mmol) was added to a solution of dimethylformamide (0.5 mL, 6.46 mmol) and dichloroethane (4 mL) at 0 °C under argon atmosphere. After 30 minutes, this Vilsmeier reagent was added dropwise to a solution of compound **2** (2 g, 1.87 mmol) in anhydrous dichloroethane (10 mL) and the reaction mixture was then heated at 70 °C overnight. After cooling to room temperature, the reaction was poured into ethyl acetate and stirred for 4 hours. The crude product was extracted by dichloromethane. The organic phase was washed several times with water and dried over sodium sulphate, filtered and concentrated under vacuum. The crude was purified by silica gel chromatography using hexane/ethyl acetate (10:1) as eluent to obtain a yellow solid (1.32 g, 66 %). ^{1}H NMR (400 MHz, $CDCl_3$): δ 9.83 (s, 2H), 7.65 (s, 2H), 7.59 (s, 2H), 7.13 (d, *J* = 8.24 Hz, 8H), 7.08 (d, *J* = 8.27 Hz, 8H), 2.56 (t, *J* = 7.8 Hz, 8H), 1.57 (m, 8H), 1.27 (m, 40H), 0.87 (t, *J* = 6.71 Hz, 12H). ^{13}C NMR (400 MHz, $CDCl_3$): 182.75, 157.00, 155.23, 150.02, 146.39, 142.22, 140.58, 135.92, 132.06, 128.65, 127.66, 119.17, 62.99, 35.55, 31.88, 31.35, 29.45, 29.24, 22.67, 14.11. Anal. Calcd for $C_{72}H_{90}S_2$: C, 82.63; H, 8.43; S, 5.96. Found: C, 82.76; H, 8.25; S, 5.41.

Synthesis of compound 5 (Scheme S1)

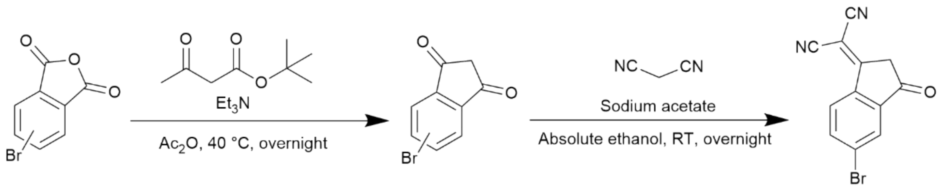

4, 96%   **5**, 19%

5-Bromo-1H-indene-1,3(2H)-dione (4). 4-bromophthalic anhydride (10.0 g, 44.05 mmol) was dissolved in acetic anhydride (62 mL) in a 250 ml three-necked round bottom flask under argon atmosphere. Then t-butyl acetoacetate (9.43 mL, 57.27 mmol) was successively added to the reaction flask and heated at 40 °C. Finally, triethylamine (16 mL, 114.53 mmol) was slowly added to the reaction that was stirred overnight at 40 °C. The reaction was then poured into a beaker containing ice water (160 mL) and concentrated HCl (60 mL), and the mixture was heated to 55 °C for about 30 minutes. After cooling to room temperature, the precipitate was filtered, washed with water and petroleum ether, and dried under vacuum to give a light brown powder that was used without further purification. ^{1}H NMR (400 MHz, $CDCl_3$): δ 8.12 (d, *J* = 1.28 Hz, 1H), 7.96 (dd, *J* = 1.69, 8.14 Hz, 1H), 7.85 (d, *J* = 8.12 Hz, 1H), 3.25 (s, 2H). ^{13}C NMR (400 MHz, $CDCl_3$): 193.11, 138.78, 131.35, 126.47, 124.60, 44.99. Anal. Calcd for $C_9H_5BrO_2$: C, 48.04; H, 2.20. Found: C, 47.36; H, 2.62.

2-(5-bromo-3-oxo-2,3-dihydro-1H-inden-1-ylidene)malononitrile (5). A mixture of compound **4** (9.91 g, 44.05 mmol), sodium acetate (10.84 g, 132.11 mmol) and malononitrile (5.82 g, 88.07 mmol) dissolved in absolute ethanol (120 mL) was stirred at room temperature overnight. The reaction was quenched with water and then acidified at pH 1-2 with concentrated HCl. The precipitate was filtered, washed with distilled water then oven dried at 60 °C. The crude product was recrystallized in acetone to obtain the final product (2.27 g, 19 %). ^{1}H NMR (400 MHz, $CDCl_3$): δ 8.51 (d, *J* = 8.52 Hz, 1H), 8.11 (d,

J = 1.69 Hz, 1H), 8.00 (dd, J = 1.83, 8.53 Hz, 1H), 3.74 (s, 2H). ^{13}C NMR (400 MHz, $CDCl_3$): 193.49, 165.07, 141.80, 141.04, 139.28, 131.71, 128.10, 127.18, 112.16, 111.99, 79.76, 43.35. Anal. Calcd for $C_{12}H_5BrN_2O$: C, 52.78; H, 1.85; N, 10.26. Found: C, 51.82; H, 1.86; N, 10.17.

Synthesis of IDTe and LuNi-1 (Scheme S3)

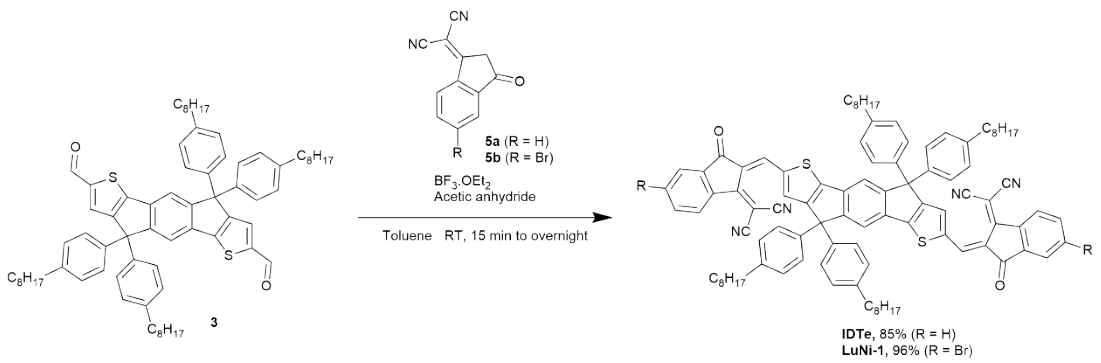

IDTe and LuNi-1. Under argon atmosphere, compound **5a** (273 mg, 1.41 mmol) or compound **5b** (335 mg, 1.3 mmol) and compound **3** (699 mg, 0.65 mmol) were dissolved in toluene (33 mL). Acetic anhydride (0.7 mL) was added and the mixture was stirred until becoming homogeneous. Then, boron trifluoride etherate (0.8 mL, 6.5 mmol) was added dropwise and the solution was stirred at room temperature overnight. The solvent was removed under reduced pressure and the crude product was precipitated twice in methanol. The precipitate was filtered and dried under vacuum to obtain a dark blue solid **IDTe** (440 mg, 85 %) or **LuNi-1** (1.03 g, 96 %).

IDTe ^{1}H NMR (400 MHz, $CDCl_3$): δ 8.90 (s, 2H), 8.69 (d, J = 7.04 Hz, 2H), 7.91, (dd, J = 1.90, 6.51 Hz, 2H), 7.73 (m, 8H), 7.14 (d, J = 8.46 Hz, 8H), 7.11 (d, J = 8.47 Hz, 8H), 2.58 (t, J = 7.60 Hz, 8H), 1.60 (m, 8H), 1.27 (m, 40H), 0.86 (t, J = 6.79 Hz, 12H). ^{13}C NMR (400 MHz, $CDCl_3$): 188.40, 160.34, 158.76, 157.95, 156.36, 142.38, 141.49, 140.34, 139.98, 139.41, 138.48, 137.11, 136.91, 135.24, 134.54, 128.79, 127.65, 125.38, 125.05, 123.83, 122.52, 120.00, 114.59, 69.46, 63.00, 35.57, 32.22, 31.90, 31.35, 29.71, 29.48, 29.43, 29.24, 26.41, 23.44, 22.67, 14.12. Anal. Calcd for $C_{98}H_{98}Br_2N_4O_2S_2$: C, 82.43; H, 6.92; N, 3.92; S, 4.49. Found: C, 81.68; H, 6.85; N, 3.28; S, 4.03. MALDI-TOF calcd for $C_{98}H_{98}N_4O_2S_2$ $[M+H]^+$: 1427.72; found: 1428.2489.

LuNi-1 ^{1}H NMR (400 MHz, $CDCl_3$): δ 8.91 (s, 2H), 8.54 (d, J = 8.49 Hz, 2H), 8.00 (d, J = 1.76 Hz, 2H), 7.86 (dd, J = 1.94, 8.47 Hz, 2H), 7.73 (m, 4H), 7.13 (m, 16H), 2.58 (t, J = 7.70, 8H), 1.59 (m, 8H), 1.28 (m, 40H), 0.86 (t, J = 6.75 Hz, 12H). ^{13}C NMR (400 MHz, $CDCl_3$): 187.01, 159.55, 158.40, 156.68, 142.59, 141.72, 140.31, 139.34, 139.03, 138.50, 138.36, 138.04, 137.33, 129.88, 128.95, 127.77, 127.10, 126.66, 121.96, 120.32, 114.60, 114.51, 69.72, 63.12, 35.69, 32.02, 31.47f, 29.59, 29.54, 29.36, 22.79, 14.24. Anal. Calcd for $C_{98}H_{96}Br_2N_4O_2S_2$: C, 74.23; H, 6.10; N, 3.53; S, 4.04. Found: C, 73.05; H, 5.98; N, 3.38; S, 3.99. MALDI-TOF calcd for $C_{98}H_{96}Br_2N_4O_2S_2$ $[M+H]^+$: 1585.54; found: 1587.0521.

Synthesis of compound 6 (Scheme S4)

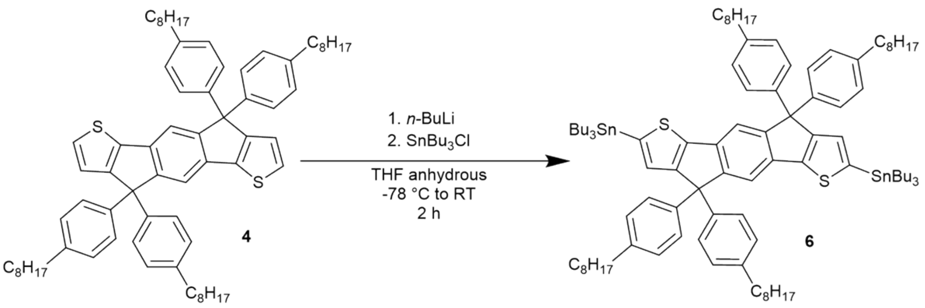

(4,4,9,9-tetrakis(4-octylphenyl)-4,9-dihydro-s-indaceno[1,2-b:5,6-b']dithiophene-2,7-diyl)bis-(tributylstannane) (6).

Under argon atmosphere, compound **4** (200 mg, 0.2 mmol) was dissolved in anhydrous THF (30 mL). The mixture was bubbled with argon during 15 minutes. After cooling to -78 °C, n-butillithium (0.2 mL, 2.5 M, 0.4 mmol) was added dropwise to the mixture and then the solution was stirred at -78 °C for 1 hour. After one hour, tributyltin chloride (0.15 mL, 0.4 mmol) was added dropwise at -78 °C. Then the reaction mixture was allowed to warm to room temperature and stirred for 2 hours before being quenched with water. The organic phase was extracted with ether, washed three times with a saturated ammonium chloride solution, dried over Na_2SO_4 and concentrated under vacuum. The crude product was used without further purification due to the high toxicity (333 mg, 95 %). ^1H NMR (400 MHz, $CDCl_3$): δ 7.43 (s, 2H), 7.18 (d, *J* = 7.90 Hz, 8H), 7.06 (d, *J* = 8.00 Hz, 8H), 7.03 (s, 2H), 2.57 (t, *J* = 7.74 Hz, 8H), 1.57 (m, 20H), 1.29 (m, 70H), 0.90 (t, *J* = 7.03 Hz, 30H).

Synthesis of compound 10 (Scheme S5)

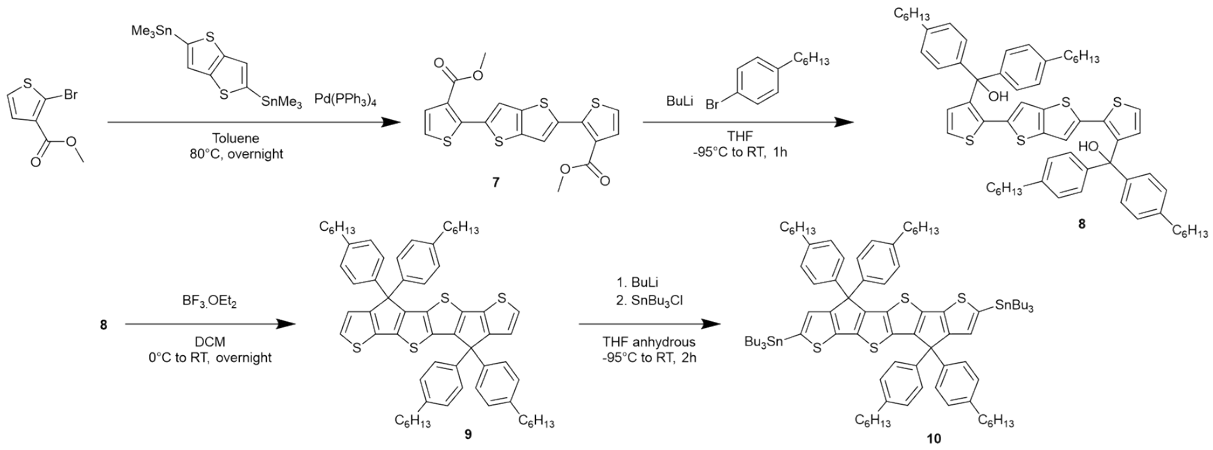

Dimethyl 2,2'-(thieno[3,2-b]thiophene-2,5-diyl)bis(thiophene-3 carboxylate) (7).

Methyl-2-bromothiophene-3-carboxylate (1.1 g, 5.2 mmol) and 2,5-bis(trimethylstannyl)thieno[3,2-b]thiophene (1 g, 2.1 mmol) were dried under vacuum and placed under argon. Toluene (50 mL) was added and the solution was degassed for 10 minutes with argon. Tetrakis(triphenylphosphine)palladium(0) (124 mg, 0.1 mmol) was added and the mixture was further degassed for 5 minutes. The solution was then heated to 80 °C overnight. At the end of the reaction, methanol (10 mL) was added, the mixture was cooled down and the yellow solid was recovered by filtration, washed with ethanol, hexane and diethyl ether and dried under vacuum to obtain the final product as a yellow solid (707 mg, 78 %). ^1H NMR (400 MHz, $CDCl_3$): δ 7.70 (s, 2H), 7.54 (d, *J* = 5.44 Hz, 2H), 7.27 (d, *J* = 5.44 Hz, 2H), 3.88 (s, 6H). ^{13}C NMR (400 MHz, $CDCl_3$): 163.47, 143.16, 136.19, 130.68, 127.91, 124.54, 121.26, 51.88.

(thieno[3,2-b]thiophene-2,5-diylbis(thiophene-2,3-diyl))bis(bis(4-hexylphenyl)methanol) (8). n-BuLi (3.14 mL, 2.5 M, 7.8 mmol) was added dropwise to a solution of 1-bromo-4-hexylbenzene (2.1 g, 8.5 mmol) in anhydrous THF (150 mL) at -95 °C. The reaction mixture was stirred 30 minutes before compound **7** (600 mg, 1.4 mmol) was added as a solid at -95 °C. The mixture was further stirred at this temperature for 5 minutes and then allowed to reach room temperature and react for one hour. At the end of the reaction, the mixture was quenched with a saturated solution of $NaHCO_3$ (10 mL), diluted with water (50 mL) and ethyl acetate (100 mL) and stirred at room temperature for 10 minutes. The organic layer was washed with water and brine, dried over Na_2SO_4, filtered off and concentrated under vacuum. The resulting crude product was purified by column chromatography to obtain a yellow-green oil (928 mg, 64 %). ^{1}H NMR (400 MHz, $(CD_3)_2CO$): δ 7.37 (d, *J* = 5.40 Hz, 2H), 7.20 (d, *J* = 8.25 Hz, 8H), 7.10 (d, *J* = 8.19 Hz, 8H) 6.82 (s, 2H), 6.65 (d, *J* = 5.40 Hz, 2H), 5.09 (s, 2H), 2.58 (t, *J* = 7.71 Hz, 8H), 1.59 (m, 8H), 1.33 (m, 24H), 0.89 (m, 12H). ^{13}C NMR (400 MHz, $CDCl_3$): 153.41, 147.93, 135.41, 130.44, 130.24, 129.42, 128.77, 128.26, 115.03, 36.04, 35.05, 31.71, 31.17, 28.98, 28.93, 22.59, 14.10.

4,4,9,9-Tetrakis(4-hexylphenyl)-4,9-dihydrothieno[3',2':4,5]cyclopenta[1,2-b]thieno[2'',3'':3',4']-cyclopenta[1',2':4,5]thieno[2,3-d]thiophene (9). Compound **8** (920 mg, 0.9 mmol) was solubilized in anhydrous dichloromethane (100 mL) and the entire system was degassed by freeze pumping (3 times). The solution was then continuously bubbled with argon. $BF_3.OEt_2$ (2 mL, excess) was then added at 0 °C and the reaction was allowed to warm up to room temperature and react until completion (followed by TLC Hex/EtOAc 9/1). At the end of the cyclization, the mixture was quenched with a saturated solution of $NaHCO_3$ (50 mL) under argon bubbling and washed with water twice. The organic layer was washed with water and brine, dried over Na_2SO_4, filtered off and concentrated under vacuum. The resulting crude product was purified by column chromatography to obtain orange crystals (420 mg, 47 %). ^{1}H NMR (400 MHz, $CDCl_3$): δ 7.29 (s, 2H), 7.16 (m, 8H), 7.10 (m, 8H), 2.57 (t, *J* = 7.81 Hz, 8H), 1.59 (m, 8H), 1.30 (m, 24H), 0.89 (m, 12H). ^{13}C NMR (400 MHz, $CDCl_3$): 157.02, 148.44, 141.74, 139.8988, 134.93, 128.49, 127.79, 125.23, 123.27, 35.59, 31.71, 31.26, 29.14, 22.59, 14.09.

1,1'-[4,4,9,9-tetrakis(4-hexylphenyl)-4,9-dihydrothieno[3',2':4,5]cyclopenta[1,2-b]thieno[2'',3'':3',4']-cyclopenta[1',2':4,5]thieno[2,3-d]thiophene-2,7-diyl]bis[1,1,1-tributyl-stannane) (10). Compound **9** (400 mg, 0.4 mmol) was dried under vacuum and placed under argon. Distilled THF (60 mL) was then added and the solution was degassed by argon bubbling. The mixture was then cooled down to -96 °C and n-BuLi (0.66 mL, 2.5 M, 1.7 mmol) was added dropwise. The reaction mixture was then allowed to reach -20 °C before being cooled down to -96 °C. Tributyltin chloride (0.47 mL, 1,7 mmol) was then added dropwise and the reaction mixture was allowed to warm up to room temperature. At the end of the reaction, ethyl acetate (50 mL) and water (50 mL) were added and stirred for 5 minutes. The organic layer was washed with water and brine, dried over Na_2SO_3, filtered off and concentrated under vacuum. The resulting product was purified by recrystallization in methanol to obtain orange crystals (579 mg, 91 %). ^{1}H NMR (400 MHz, $CDCl_3$): δ 7.14 (d, *J* = 8.21 Hz, 8H), 7.06 (d, *J* = 8.71 Hz, 8H), 7.04 (s, 2H), 2.54 (t, *J* = 7.80 Hz, 8H), 1.55 (m, 32H), 1.31 (m, 36H), 0.89 (m, 30H). ^{13}C NMR (400 MHz, $CDCl_3$): 158.91, 148.12, 142.81, 141.47, 140.37, 137.76, 134.75, 131.03, 128.36, 127.92, 61.34, 35.60, 31.71, 31.27, 29.16, 28.98, 27.21, 22.59, 17.53, 14.09, 13.69, 10.98.

Synthesis of polymers (LuNi-2, LuNi-3 and LuNi-4) (Scheme S6)

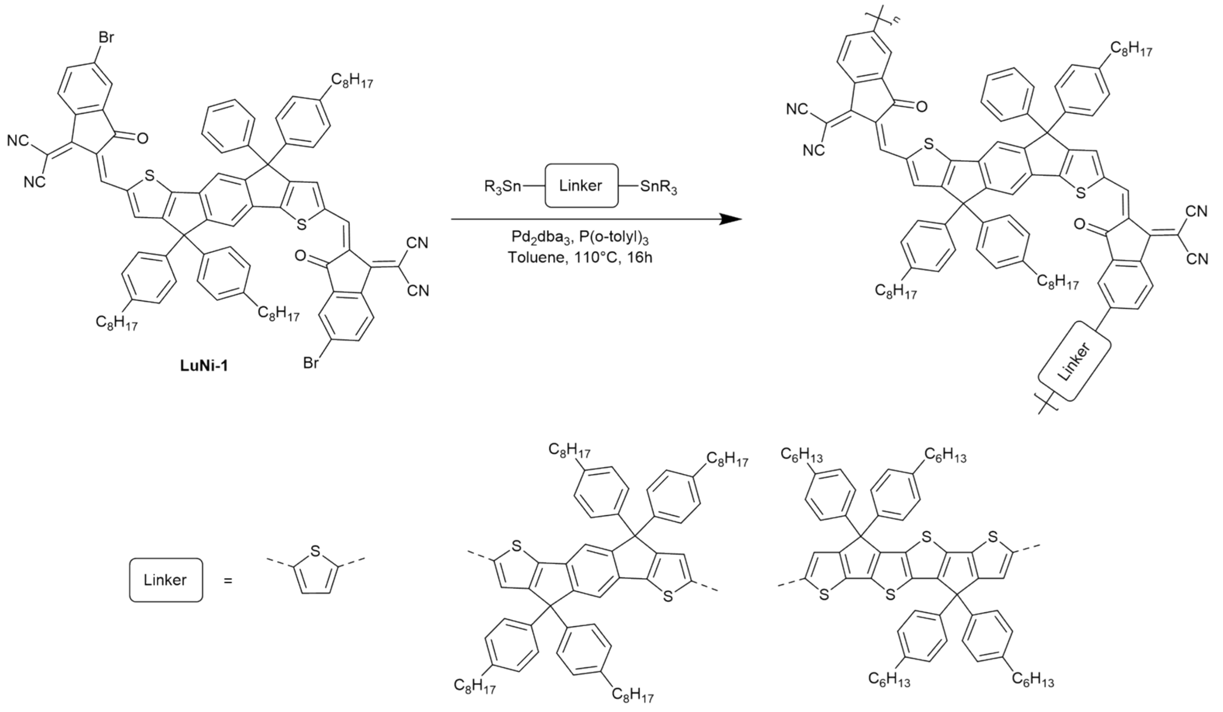

LuNi-2, 96% **LuNi-3**, 74% **LuNi-4**, 95%

LuNi-2. Under argon, in a sealed tube, **LuNi-1** (180 mg, 0.11 mmol), 2,5bis(trimethylstannyl) thiophene (46.5 mg, 0.11 mmol), tris(dibenzylideneacetone)dipalladium(0) (2.08 mg, 0.002 mmol) and tris(o-tolyl)phosphine (2.76 mg, 0.009 mmol) were dissolved in toluene (6 mL, 20 mmol.L^{-1}). The solution was stirred at 110 °C for 16 hours. After cooling to room temperature, the crude product was precipitated in methanol, filtered and then dried under vacuum. The crude polymer was scavenged by sodium diethyldithiocarbamate (1 g in 100 mL of water) overnight before precipitation in methanol, filtered, dried under vacuum and further purified by Soxhlet extraction in acetone, hexanes and then chloroform. The chloroform fractions were precipitated again in methanol and the precipitates were filtered through a 0.45 µm nylon filter and thoroughly dried under vacuum to obtain polymer **LuNi-2** (M_n = 12.7 kg/mol, M_w = 31.4 kg/mol, Đ = 2.5) as a dark blue solid (135 mg, 79 %).

LuNi-3. Under argon, in a sealed tube, **LuNi-1** (89.33 mg, 0.06 mmol), (4,4,9,9-tetrakis(4-octylphenyl)-4,9-dihydro-s-indaceno[1,2-b:5,6-b']dithiophene-2,7-diyl)bis(tributylstannane) (100 mg, 0.06 mmol), tris(dibenzylideneacetone)dipalladium(0) (1.03 mg, 0.001 mmol) and tris(o-tolyl)phosphine (1.37 mg, 0.005 mmol) were dissolved in toluene (3 mL, 20 mmol.L^{-1}). The solution was stirred at 110°C for 16 hours. After cooling to room temperature, the crude product was precipitated in methanol, filtered and then dried under vacuum. The crude polymer was scavenged by sodium diethyldithiocarbamate (1 g in 100 mL of water) overnight before precipitation in methanol, filtered, dried under vacuum and further purified by Soxhlet extraction in acetone, hexanes and then chloroform. The chloroform fractions were precipitated again in methanol and the precipitates were filtered through a 0.45 µm nylon filter and thoroughly dried under vacuum to obtain polymer **LuNi-3** (Mn = 9.6 kg/mol, Mw = 23.2 kg/mol, Đ = 2.4) as a dark blue solid (102 mg, 74 %).

<u>LuNi-4.</u> Under argon, in a sealed tube, **LuNi-1** (102.5 mg, 0.06 mmol), compound **10** (100 mg, 0.06 mmol), tris(dibenzylideneacetone)dipalladium(0) (1.18 mg, 0.001 mmol) and tris(o-tolyl)phosphine (1.57 mg, 0.005 mmol) were dissolved in toluene (3 mL, 20 mmol.L^{-1}). The solution was stirred at 110 °C for 16 hours. After cooling to room temperature, the crude product was precipitated in methanol, filtered and then dried under vacuum. The crude polymer was scavenged by sodium diethyldithiocarbamate (1 g in 100 mL of water) overnight before precipitation in methanol, filtered, dried under vacuum and further purified by Soxhlet extraction in acetone, hexanes and then chloroform. The chloroform fractions were precipitated again in methanol and the precipitates were filtered through a 0.45 µm nylon filter and thoroughly dried under vacuum to obtain polymer **LuNi-3** (Mn = 9.6 kg/mol [1], Mw = 23.2 kg/mol, Đ = 2.4) as a dark blue solid (147 mg, 74 %).

STUDY OF THE DIFFERENT PHOSPHINE FOR THE POLYMERIZATION

Entry	Phosphine	Cone angle (°)	Tolman electronic parameter (cm^{-1})
**1**	PPh_3	145	2068.9
**2**	$P(o\text{-tolyl})_3$	194	2066.6
**3**	$P(t\text{-Bu})_3$	182	2056.1
**4**	PCy_3	170	2056.4
**5**	dppf	250	2050
**6**	SPhos	227	2060

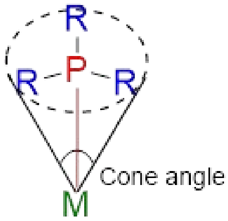

Table S1. Cone angle and Tolman electronic parameter of different phosphine ligand. The cone angle is defined as a geometric parameter that measures the steric hindrance of phosphine ligand coordinated to a metal center. It is measured as the solid angle between the outermost perimeter edge of the ligand and the metal center[8].

Experiment	Palladium source	Phosphine	Mn ($kg.mol^{-1}$)	Mw ($kg.mol^{-1}$)	Đ	Yield (%)	DP
**1**	**$Pd(PPh_3)_4$**	**6h**	4.6	13.6	2.9	84	3.1
**2**	**Pd_2dba_3**	**PPh_3**	14.0	36.4	2.6	79	9.3
**2’**	**Pd_2dba_3**	**PPh_3**	6.0	27.2	4.5	87	4.0
**3**	**Pd_2dba_3**	**$P(o\text{-tolyl})_3$**	6.7	22.9	3.4	88	4.3
**3’**	**Pd_2dba_3**	**$P(o\text{-tolyl})_3$**	6.3	32.7	5.2	98	4.2
**4**	**Pd_2dba_3**	**$P(t\text{-Bu})_3$**	1.9	2.5	1.3	55	1.3
**5**	**Pd_2dba_3**	**PCy_3**	9.9	31.9	3.2	97	6.5
**5’**	**Pd_2dba_3**	**PCy_3**	6.1	20.9	3.4	96	4.0
**6**	**Pd_2dba_3**	**dppf**	5.4	11.7	2.1	96	3.6
**7**	**Pd_2dba_3**	**SPhos**	3.8	8.8	2.3	/	2.5

Table S2. LuNi-2 polymer properties (Mn, Mw, Đ, yield, DP) as a function of palladium source and phosphine ligand.

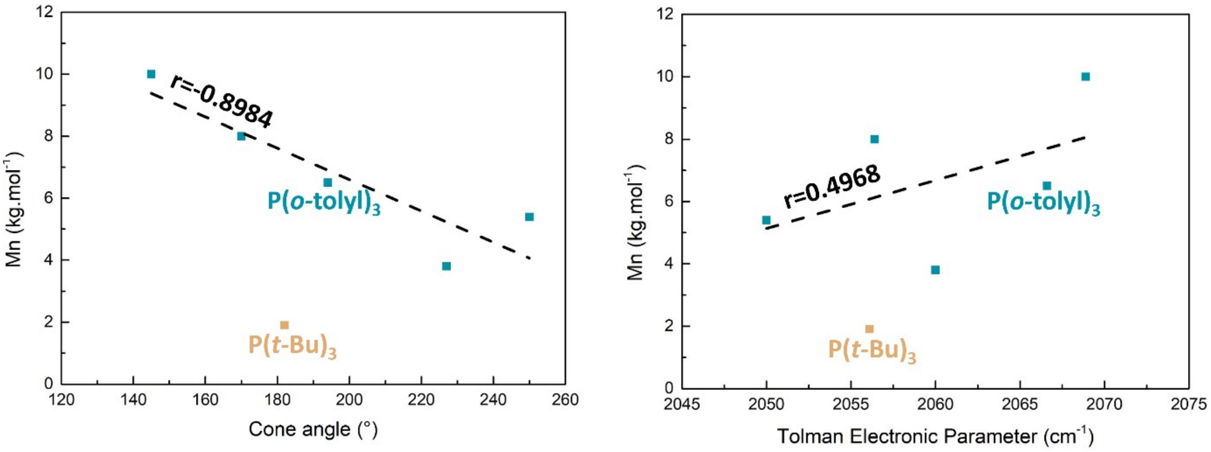

Figure S1. Mn vs Cone angle (left) and Tolman electronic parameter(right) of different phosphine ligand for LuNi-2 polymerization.

NMR SPECTRA

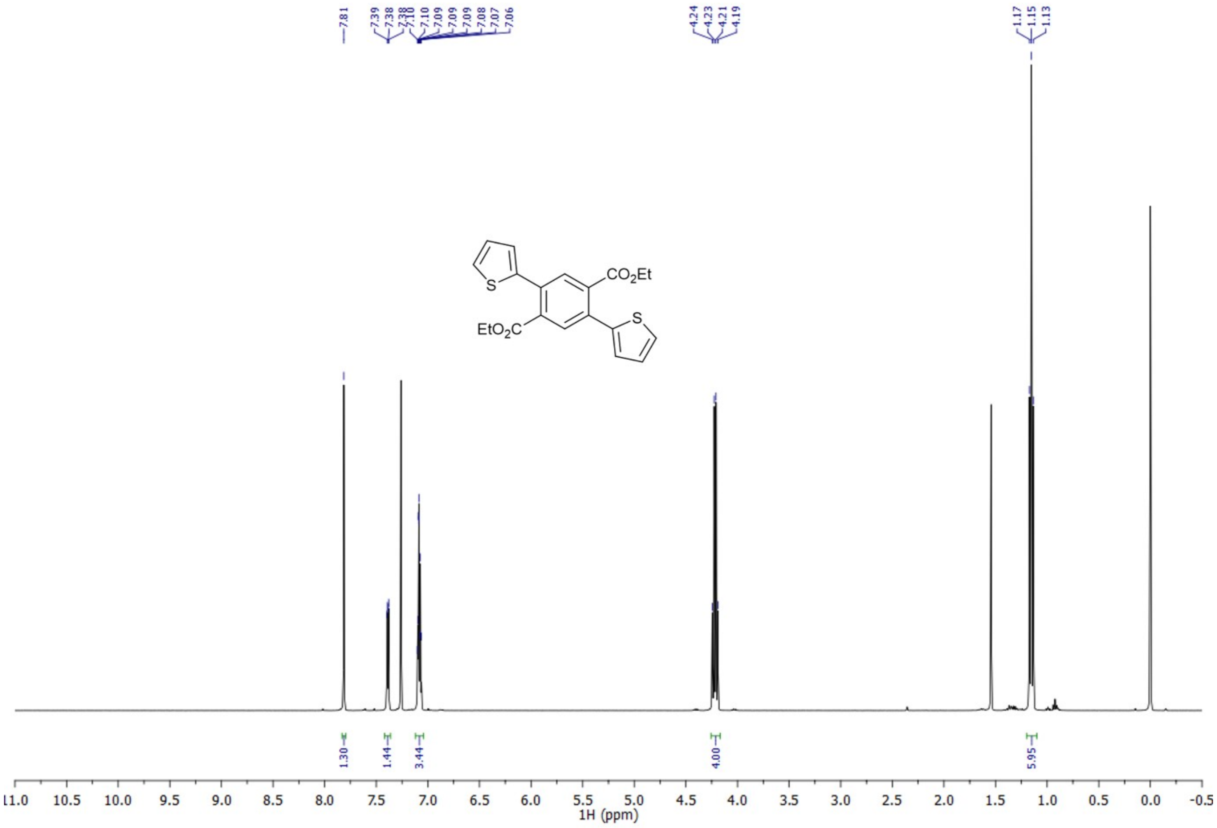

Figure S2. ^{1}H NMR Spectrum of compound 1 in $CDCl_3$.

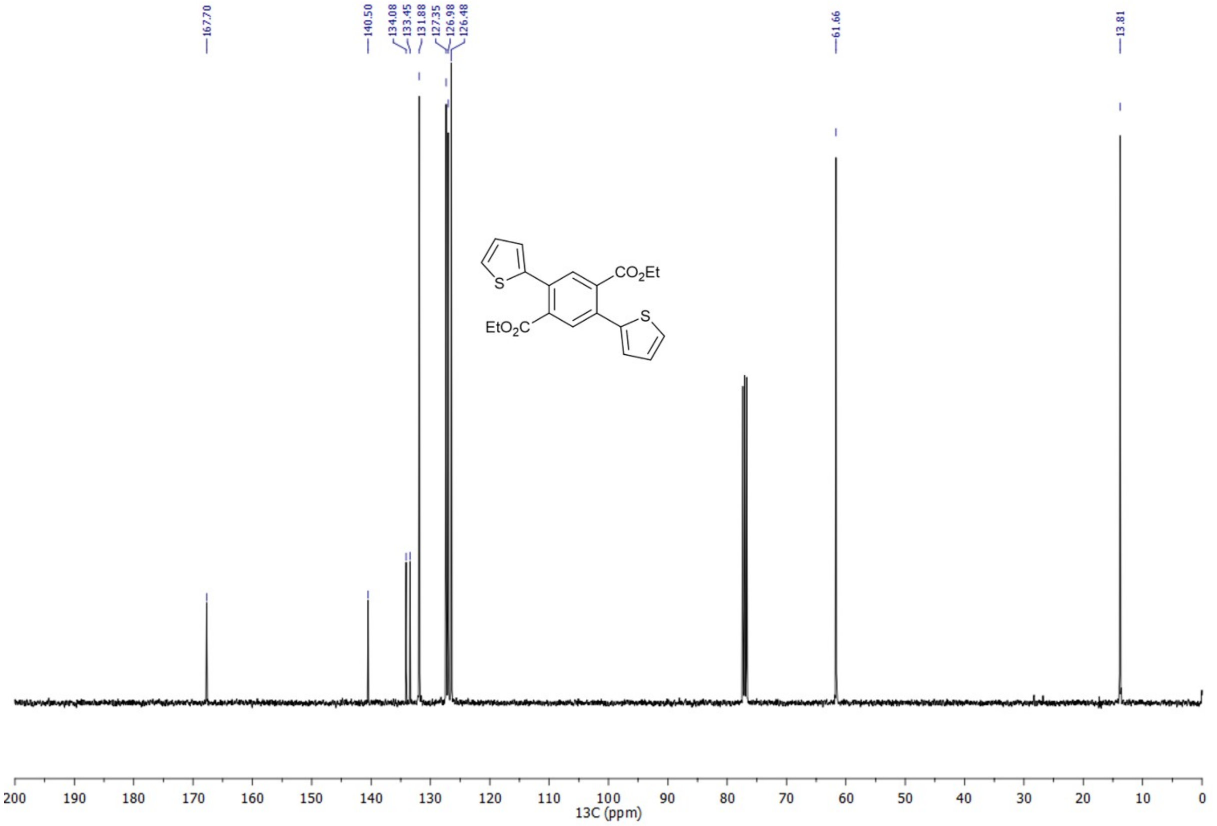

Figure S3. ^{13}C NMR Spectrum of compound 1 in $CDCl_3$.

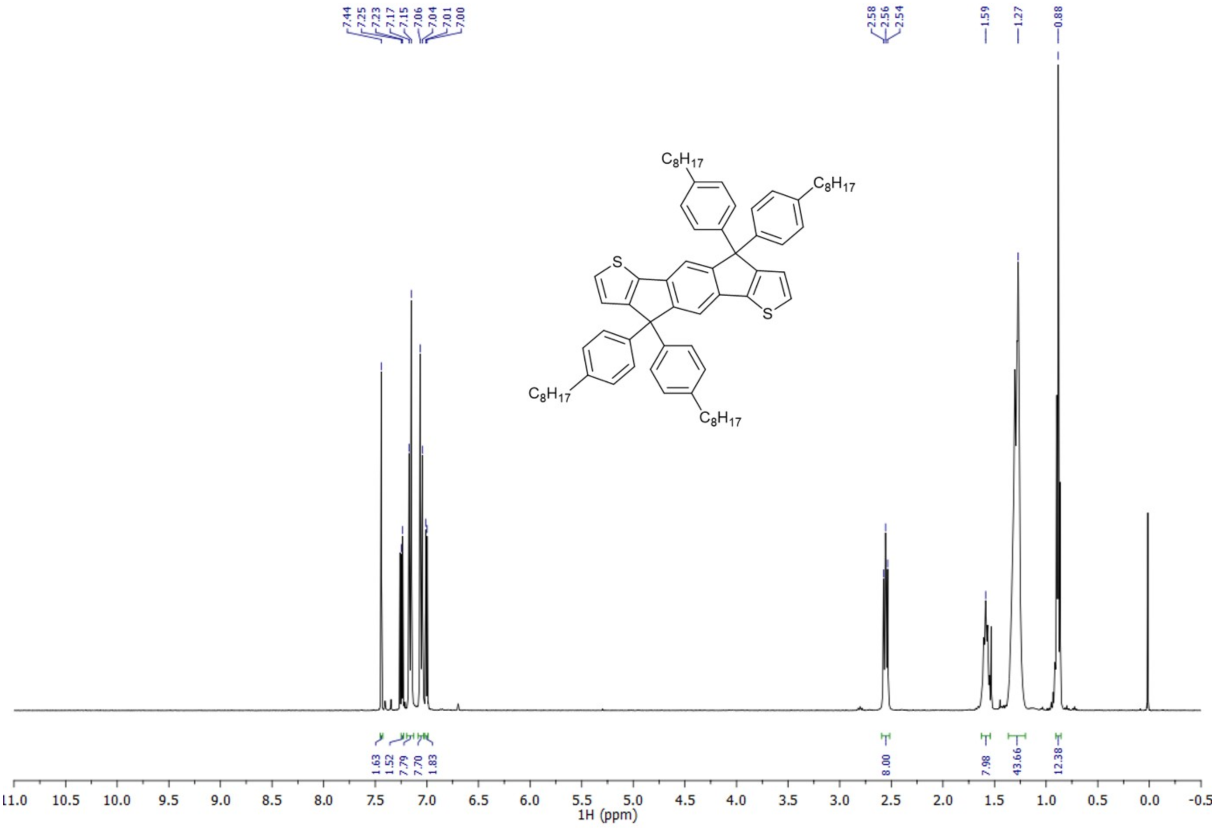

Figure S4. ^{1}H NMR Spectrum of compound 2 in $CDCl_3$.

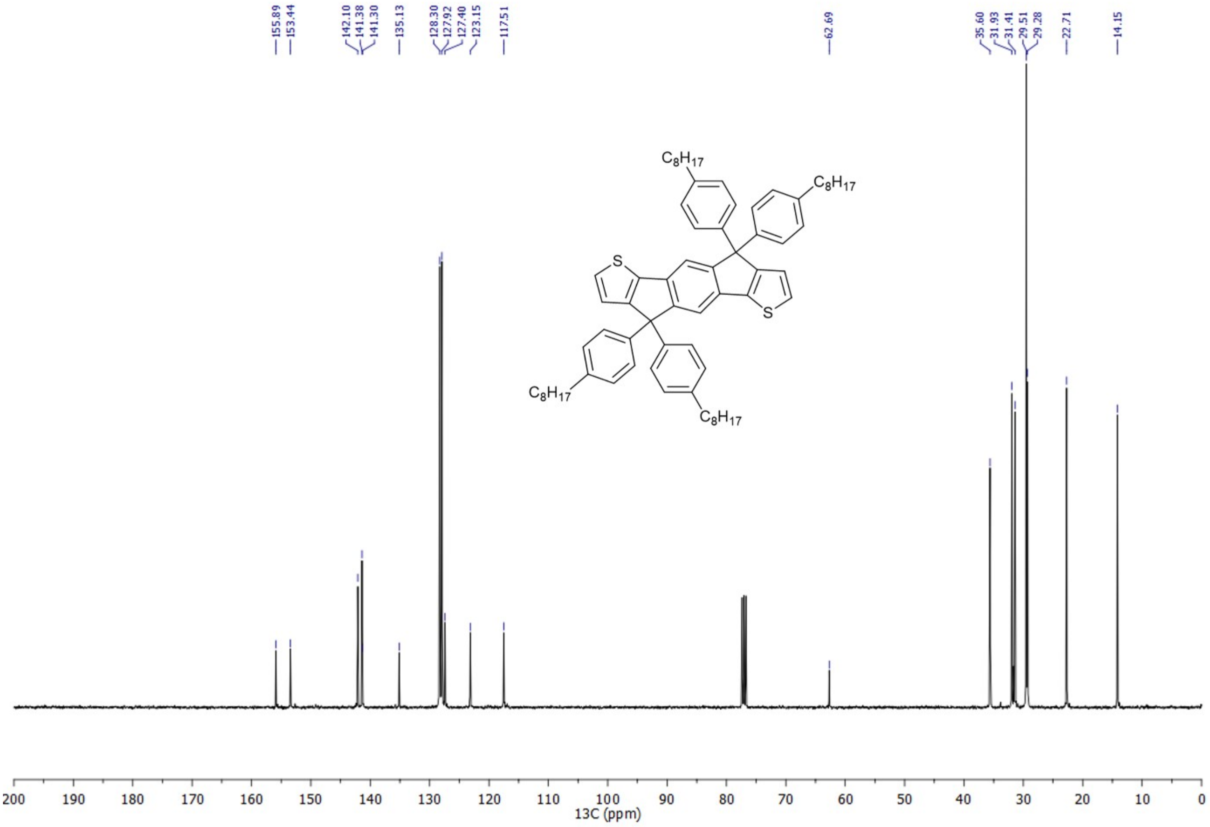

Figure S5. ^{13}C NMR Spectrum of compound 2 in $CDCl_3$.

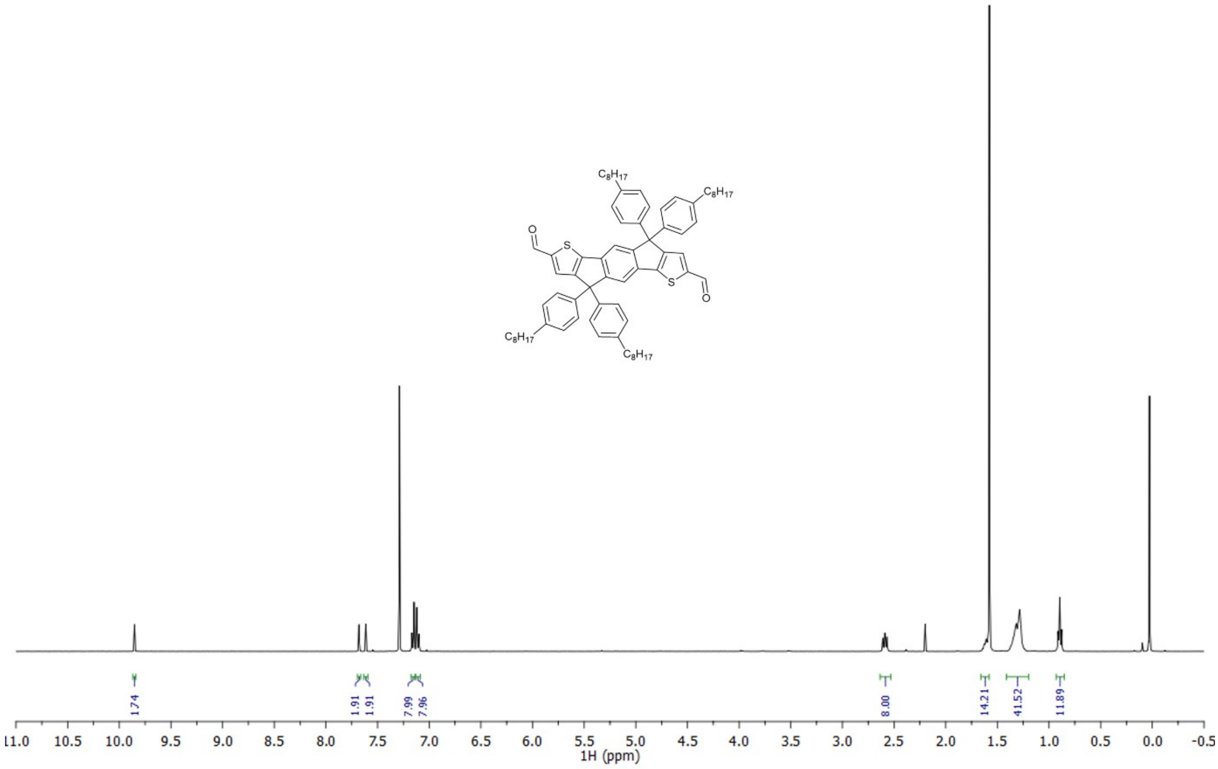

Figure S6. ^{1}H NMR Spectrum of compound 3 in $CDCl_3$.

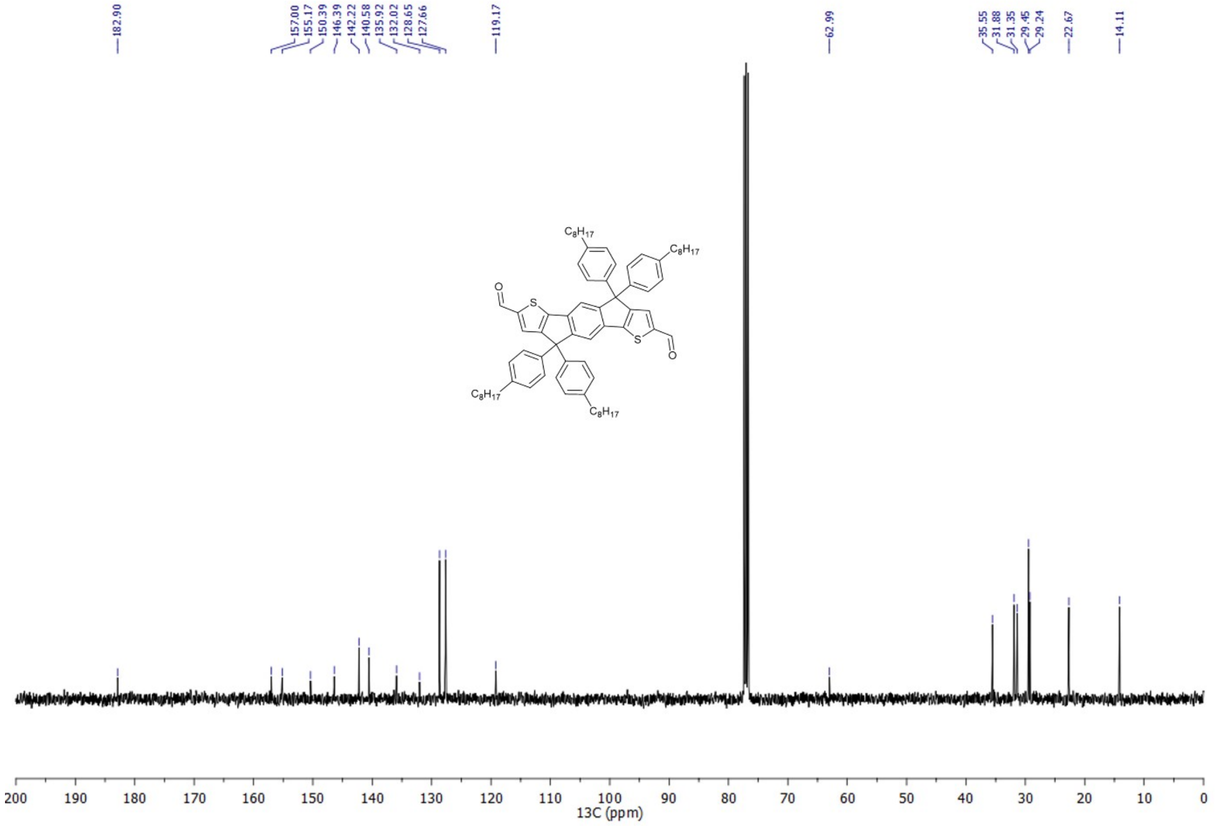

Figure S7. ^{13}C NMR Spectrum of compound 3 in $CDCl_3$.

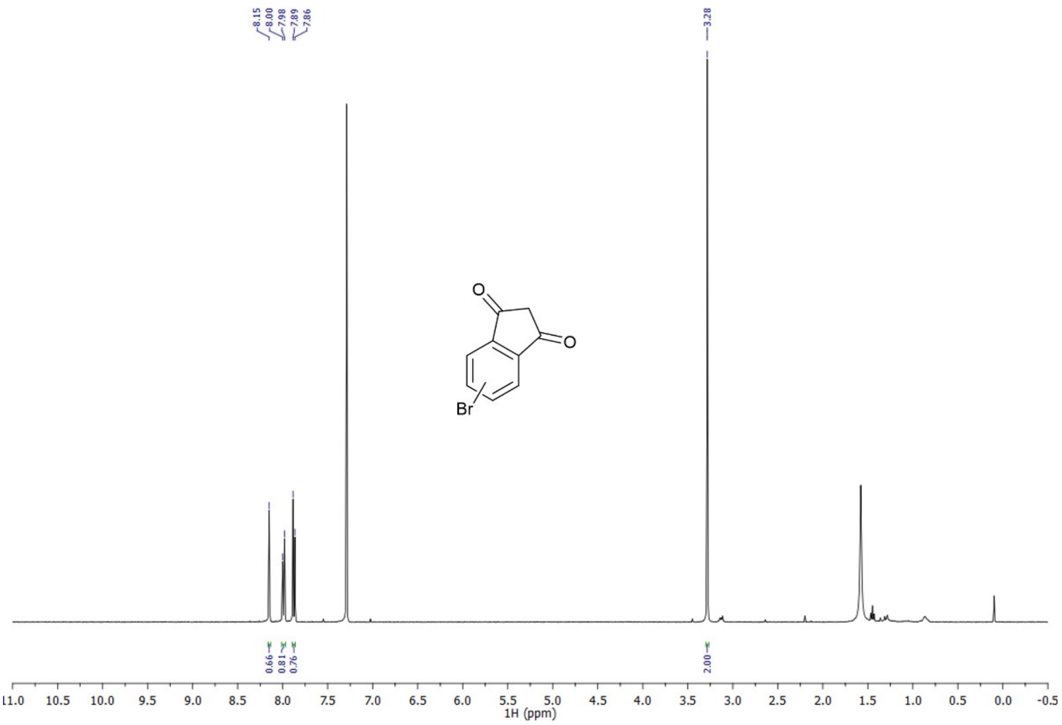

Figure S8. ^{1}H NMR Spectrum of compound 4 in $CDCl_3$.

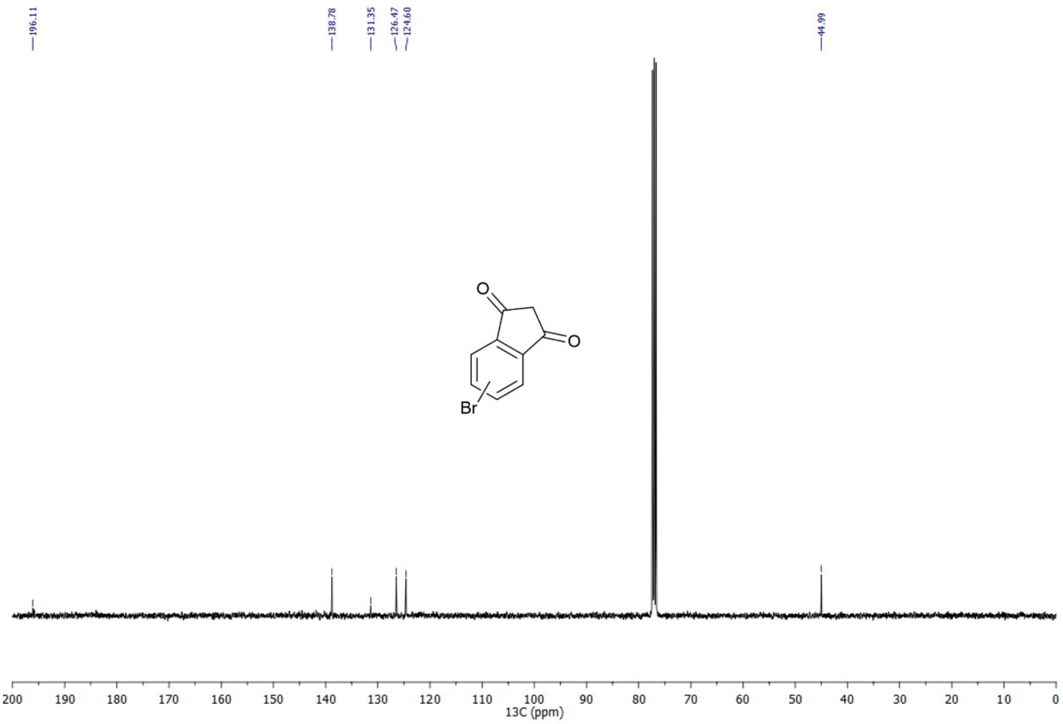

Figure S9. ^{13}C NMR Spectrum of compound 4 in $CDCl_3$.

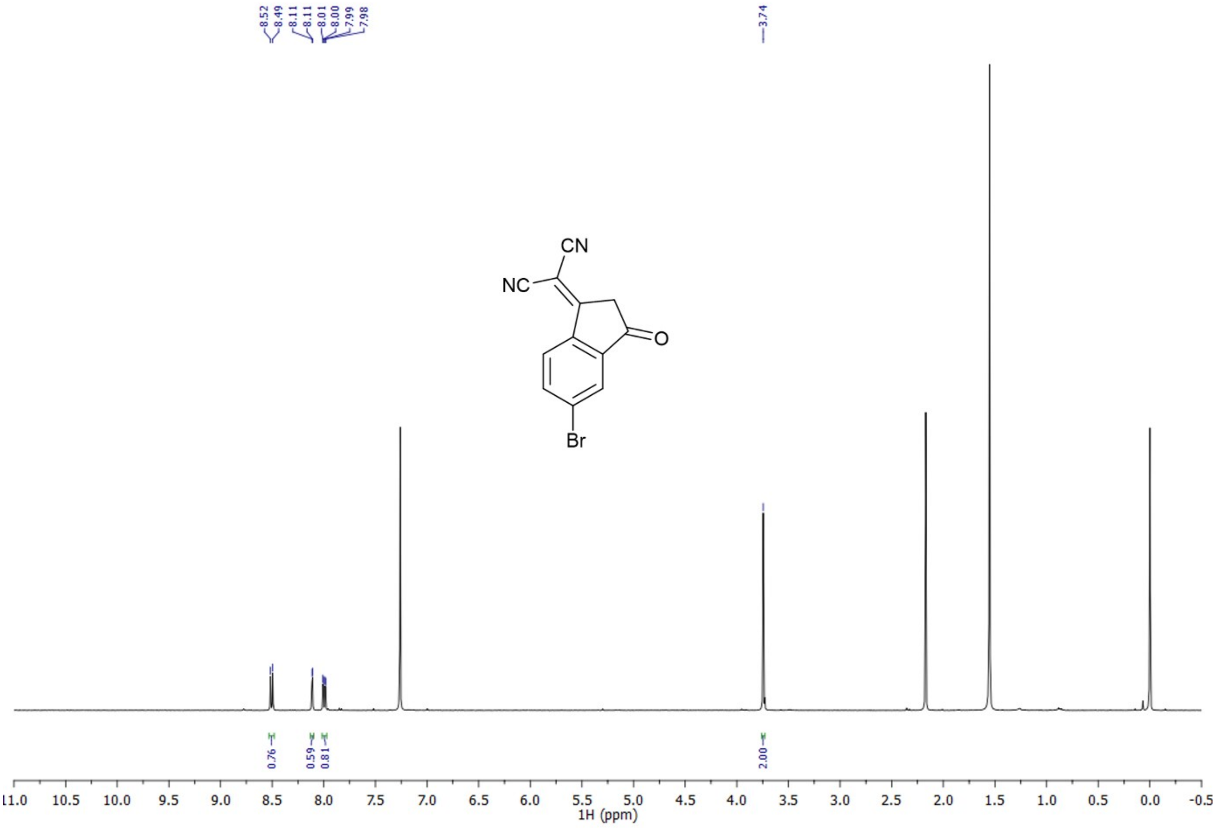

Figure S10. ^{1}H NMR Spectrum of compound 5 in $CDCl_3$.

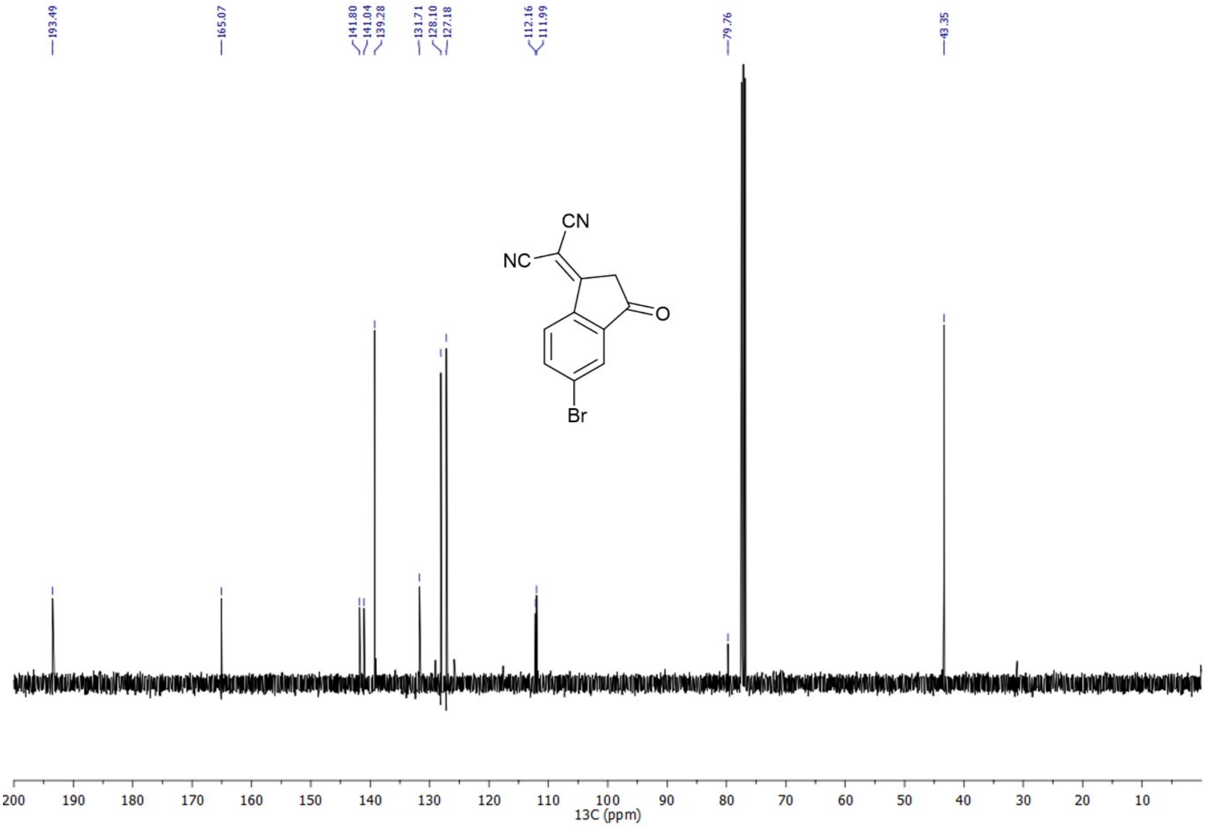

Figure S11. ^{13}C NMR Spectrum of compound 5 in $CDCl_3$.

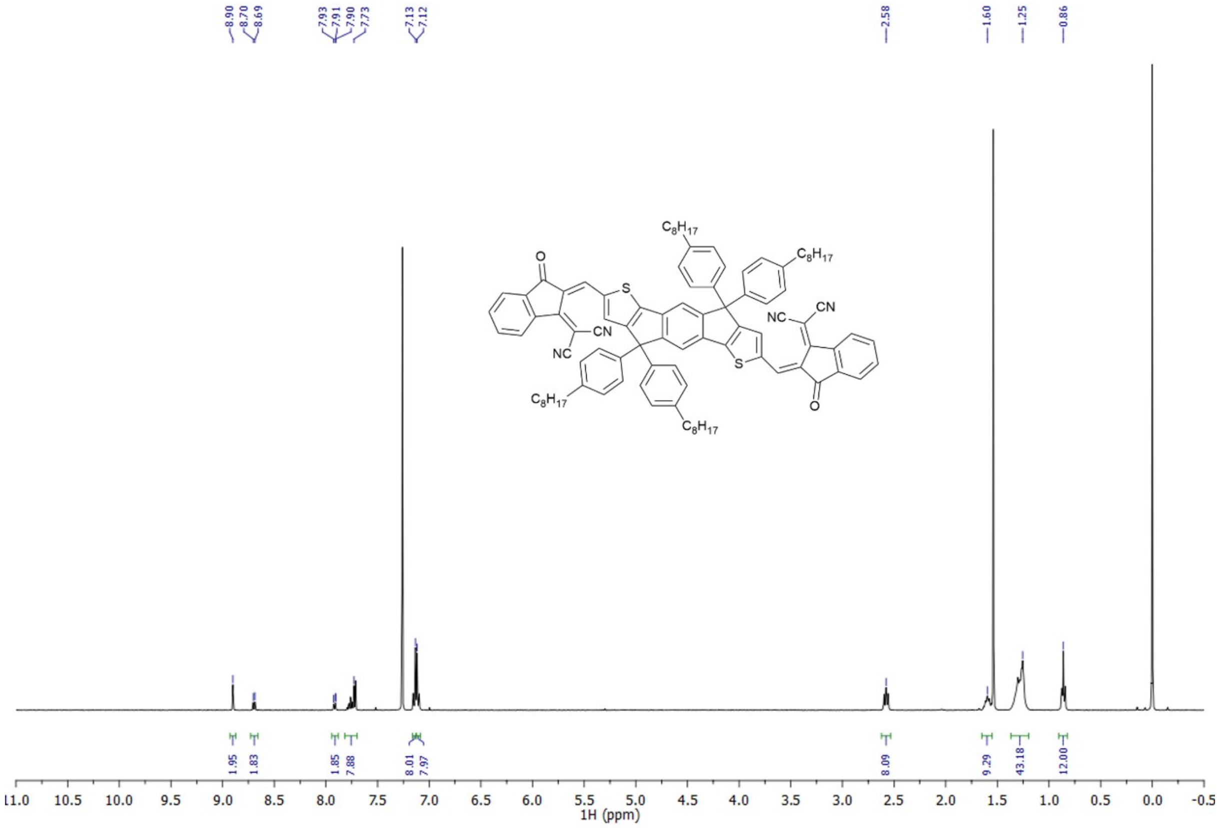

Figure S12. ^{1}H NMR Spectrum of IDTe in $CDCl_3$.

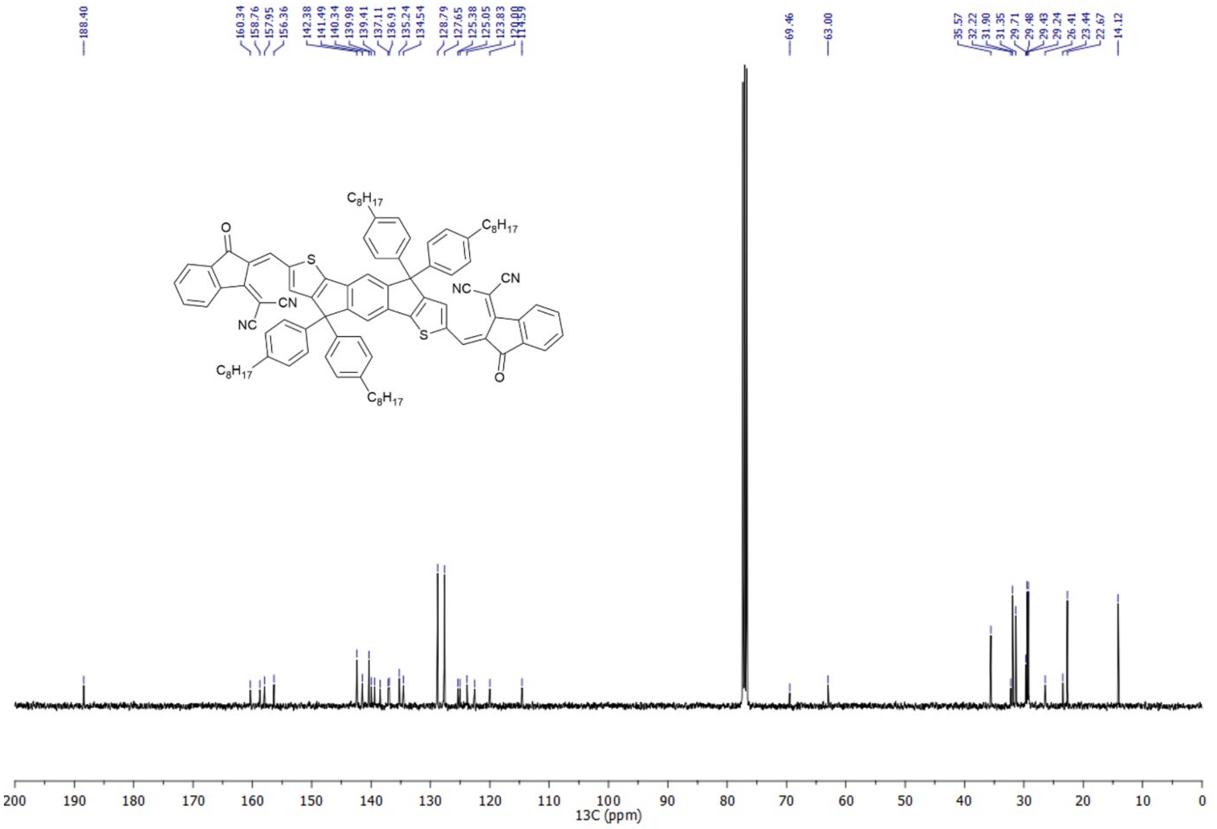

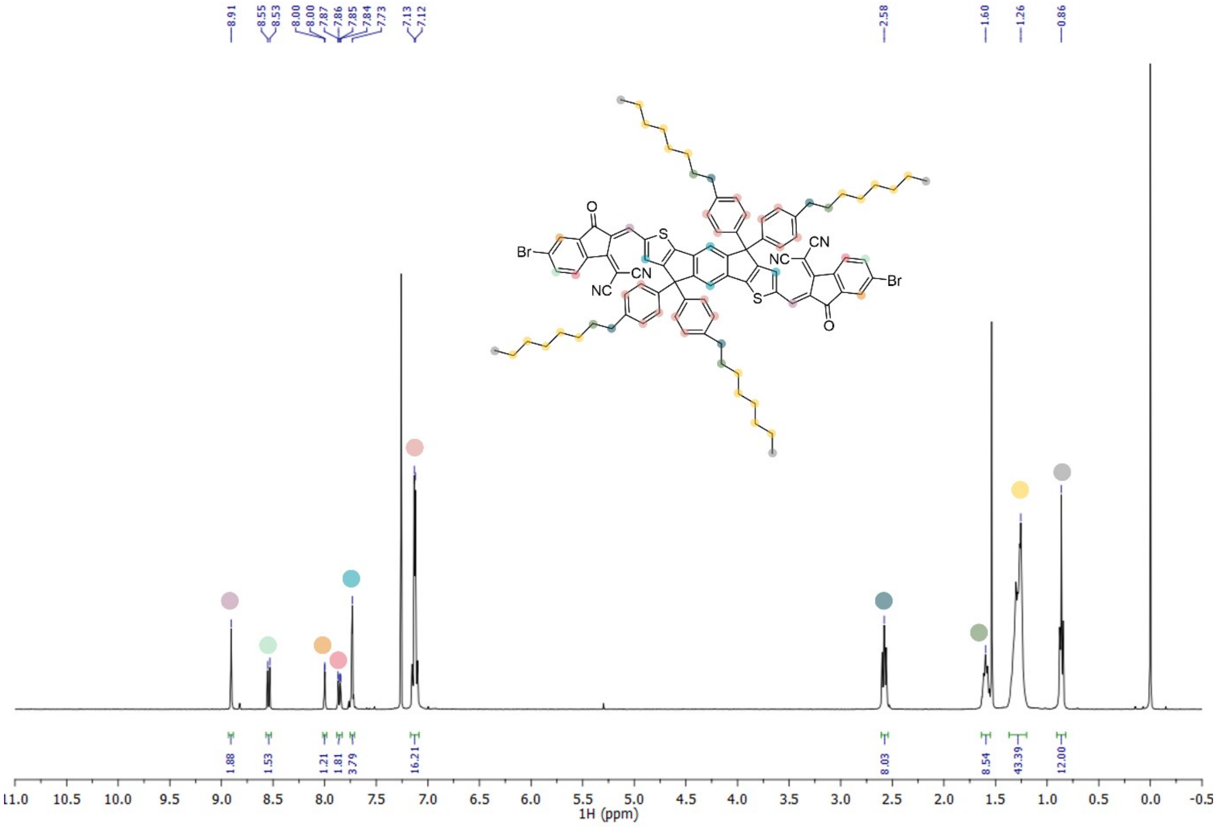

Figure S13. ^{13}C NMR Spectrum of compound IDTe in $CDCl_3$.

Figure S14. ^{1}H NMR Spectrum of LuNi-1 in $CDCl_3$.

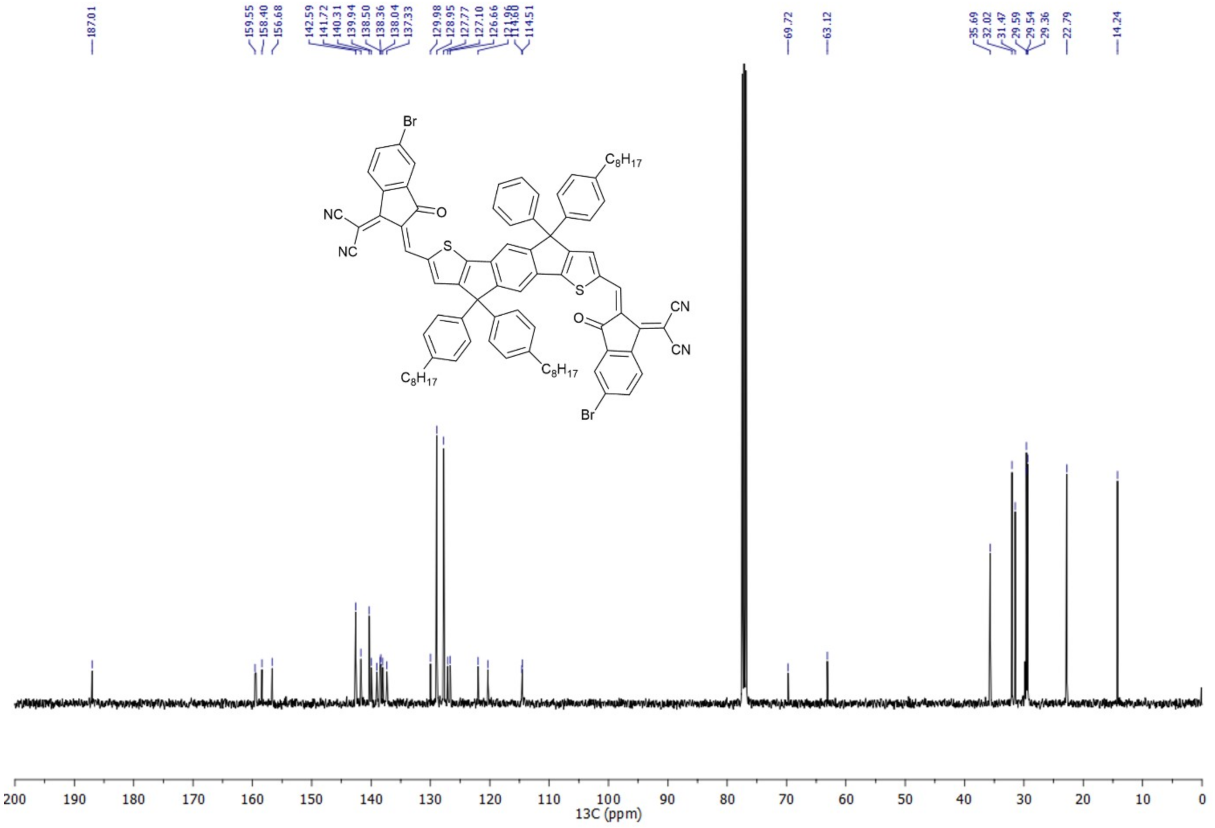

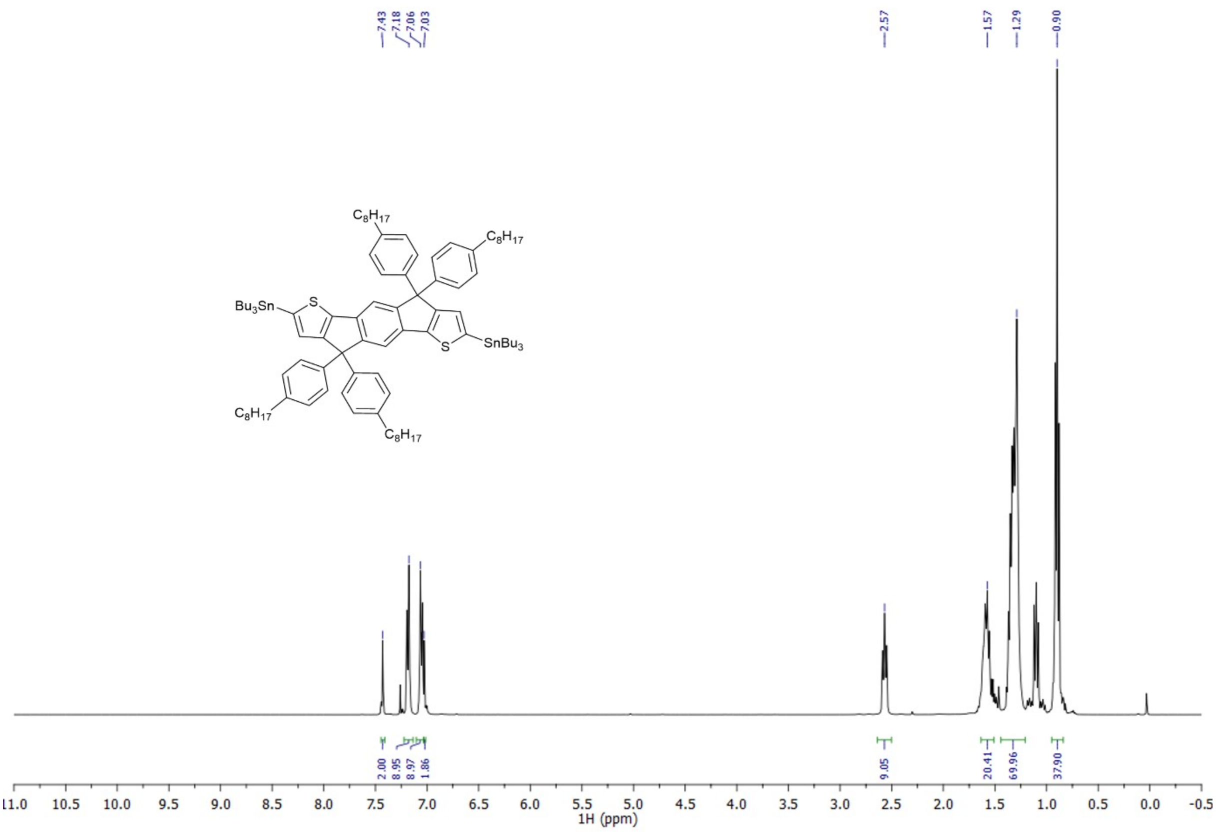

Figure S15. ^{13}C NMR Spectrum of compound LuNi-1 in $CDCl_3$.

Figure S16. ^{1}H NMR Spectrum of compound 6 in $CDCl_3$.

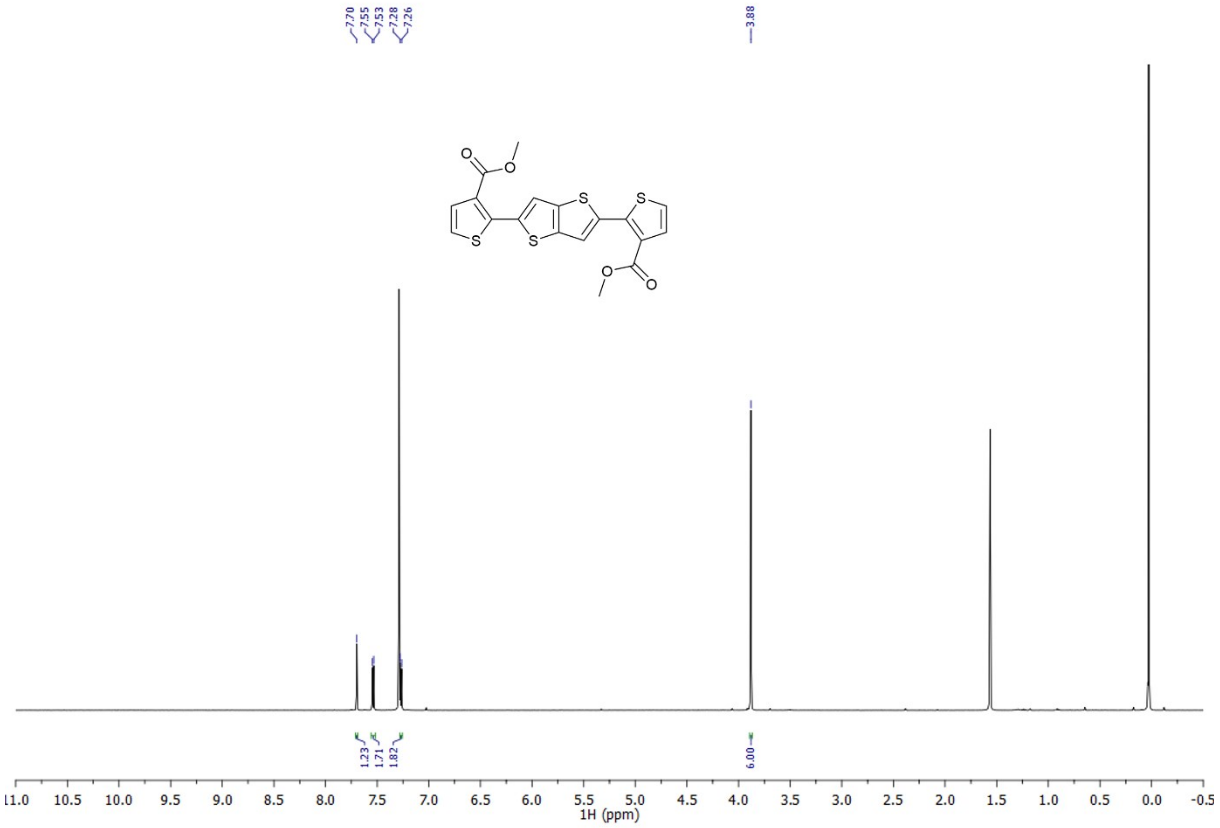

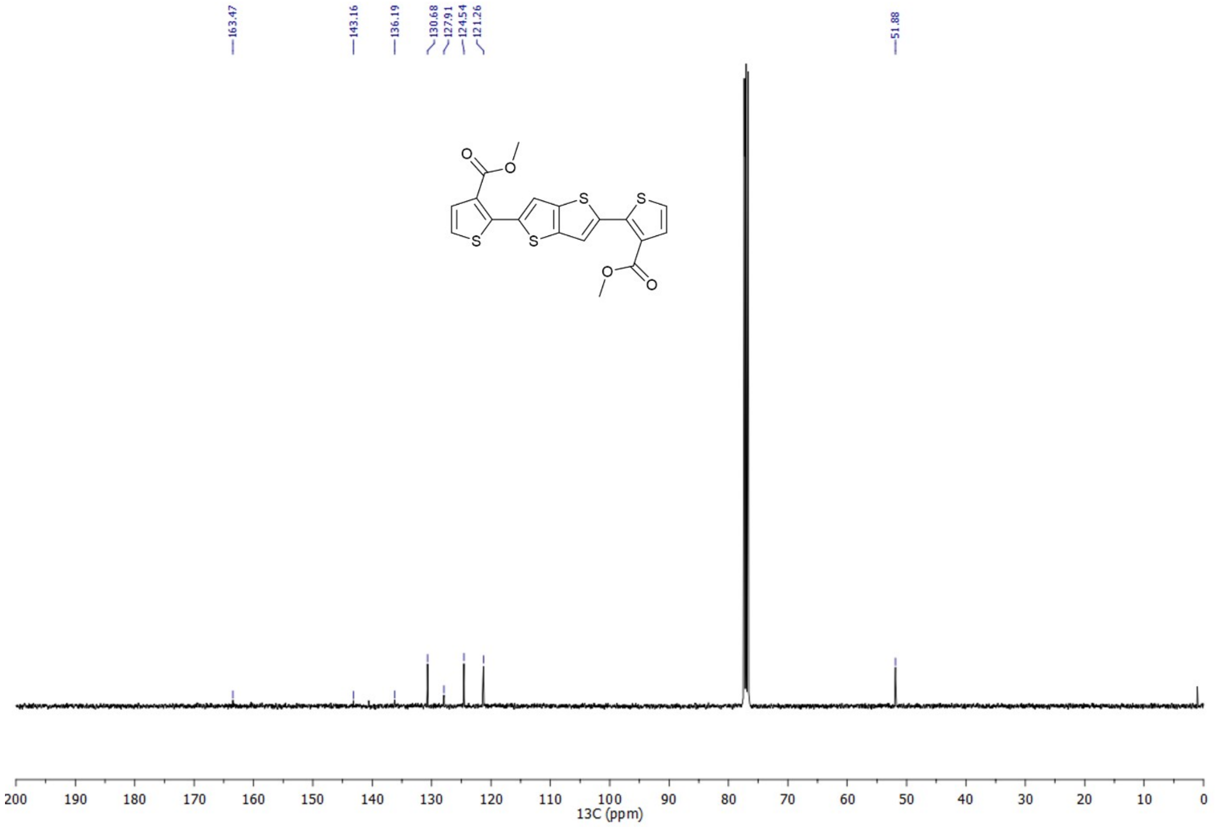

Figure S17. ^{1}H NMR Spectrum of compound 7 in $CDCl_3$.

Figure S18. ^{13}C NMR Spectrum of compound 7 in $CDCl_3$.

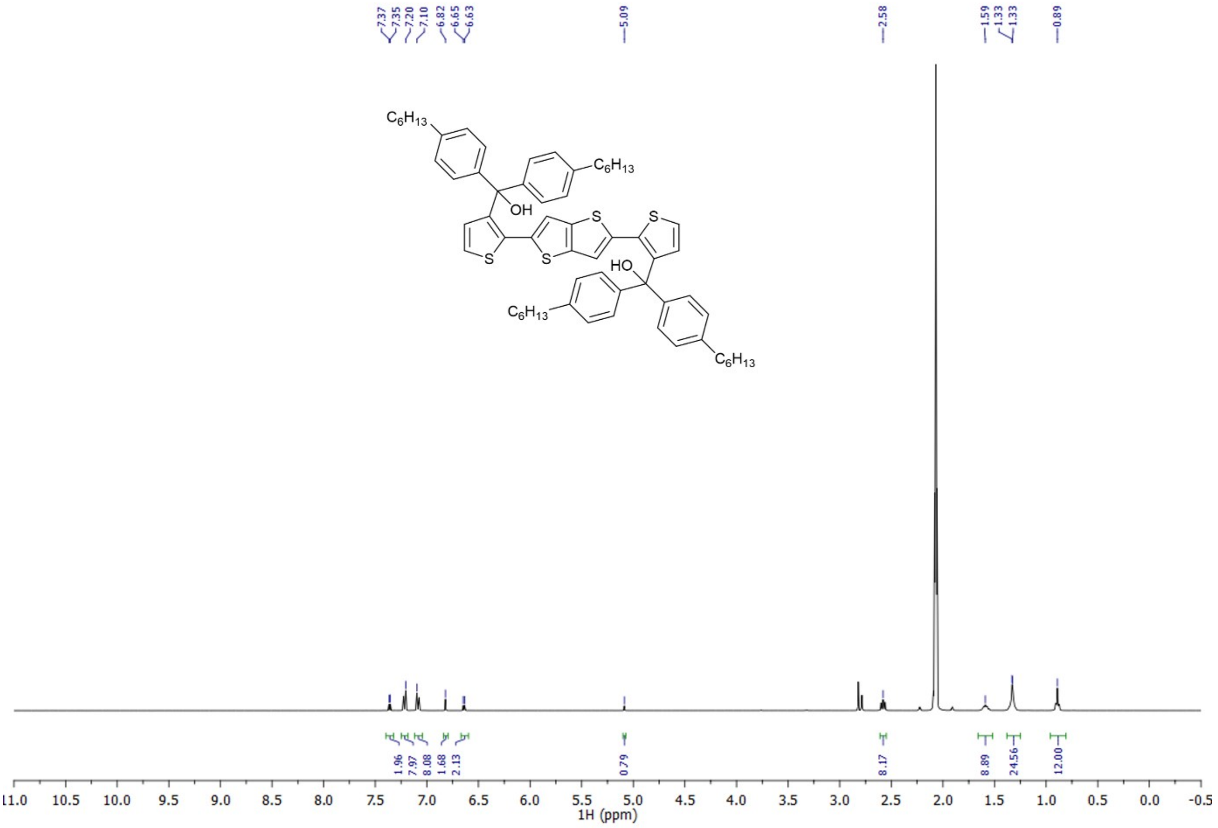

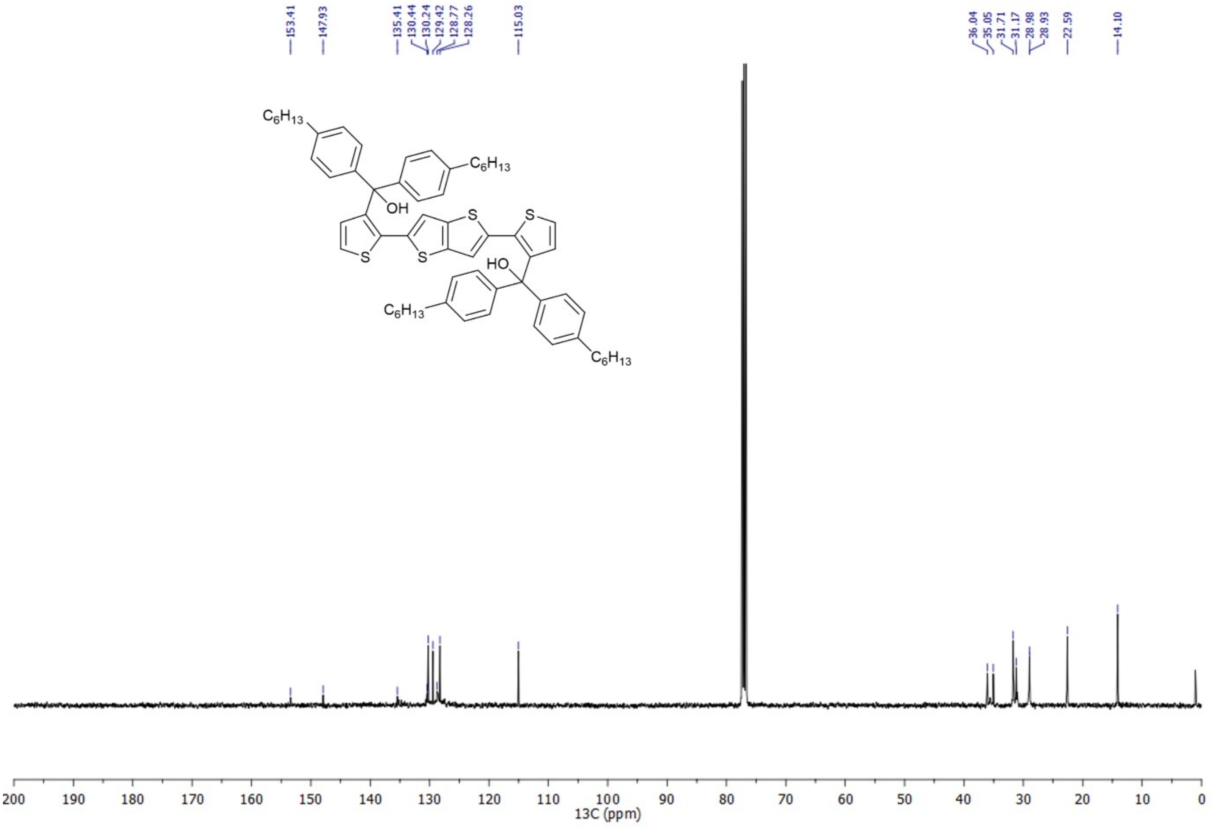

Figure S19. ^{1}H NMR Spectrum of compound 8 in $(CD_3)_2CO$.

Figure S20. ^{13}C NMR Spectrum of compound 8 in $CDCl_3$.

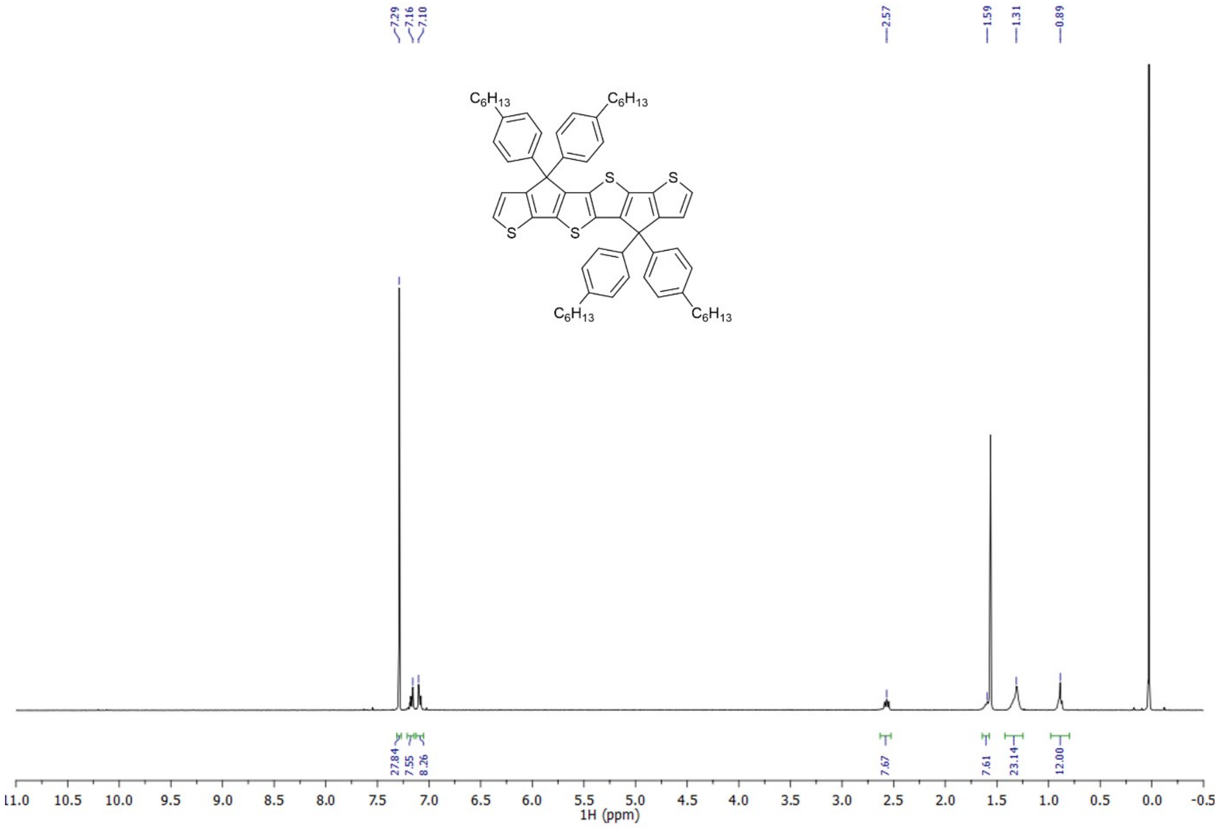

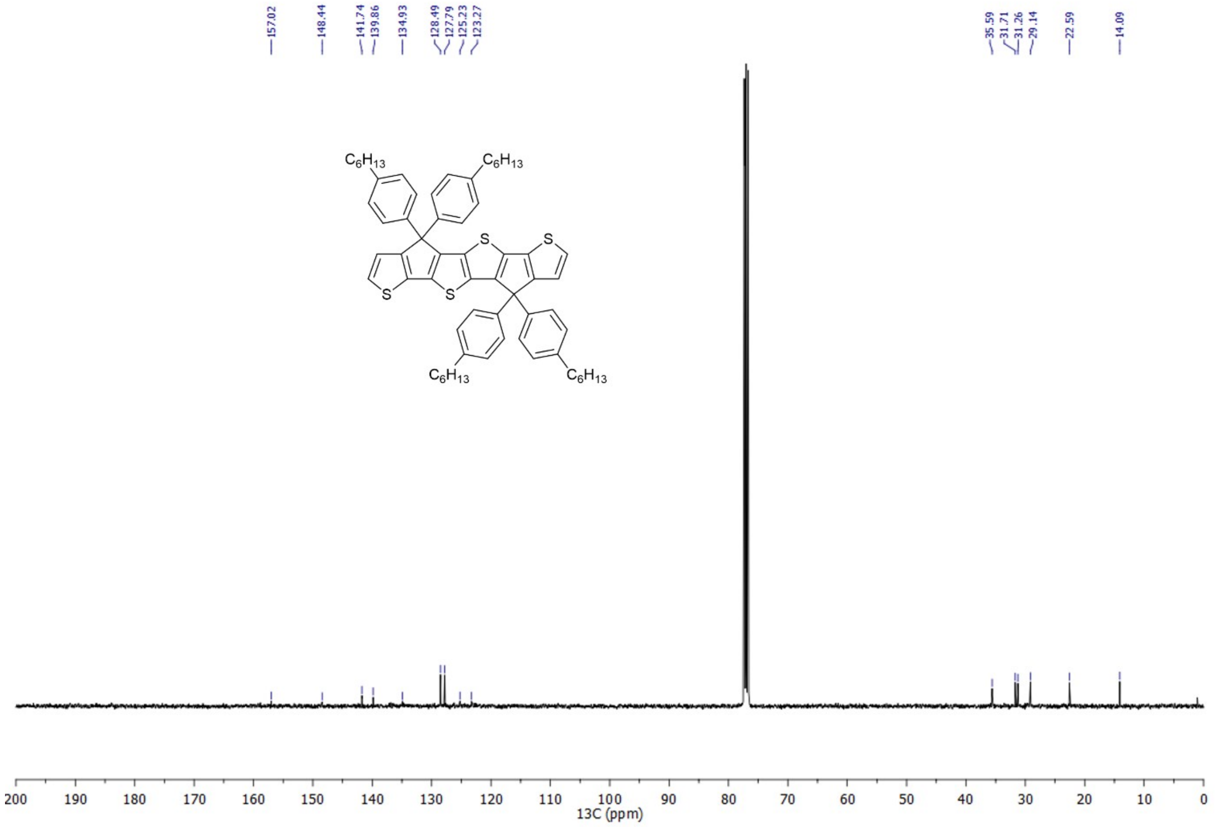

Figure S21. ^{1}H NMR Spectrum of compound 9 in $CDCl_3$.

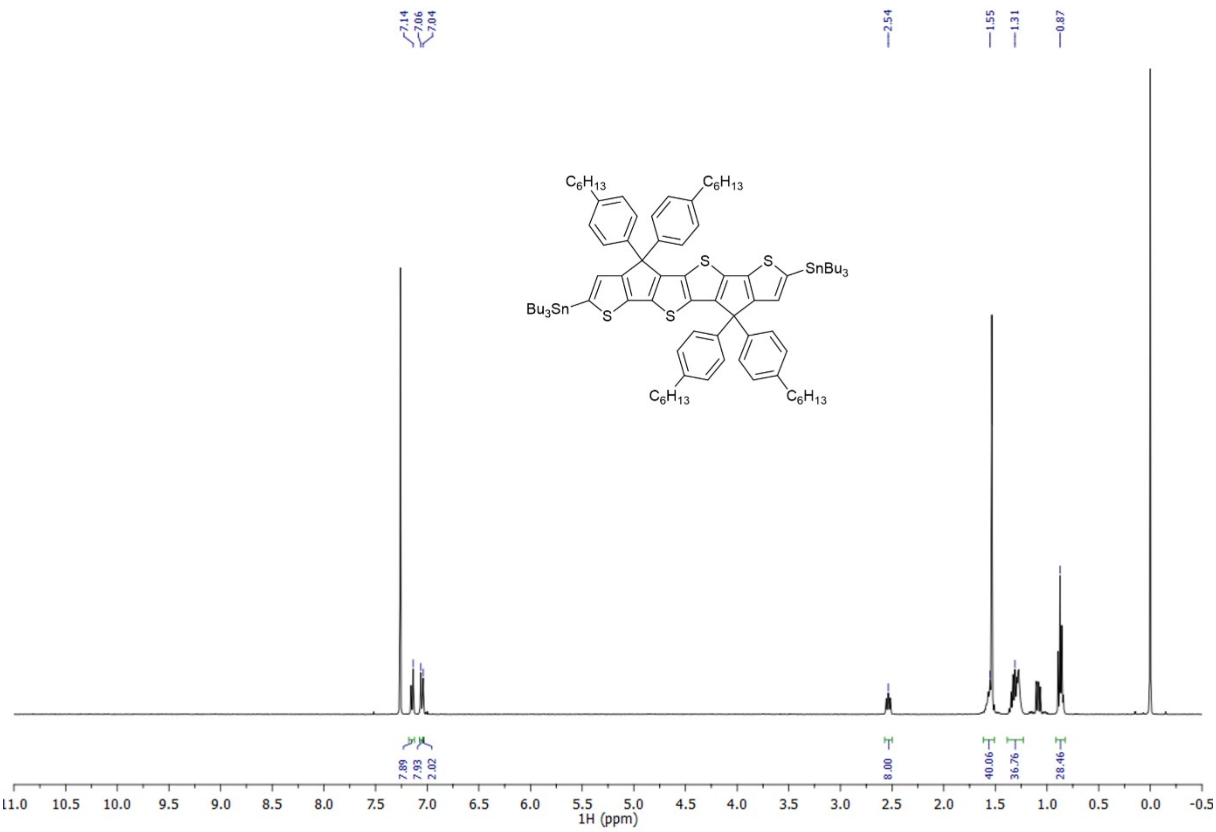

Figure S22. ^{13}C NMR Spectrum of compound 9 in $CDCl_3$.

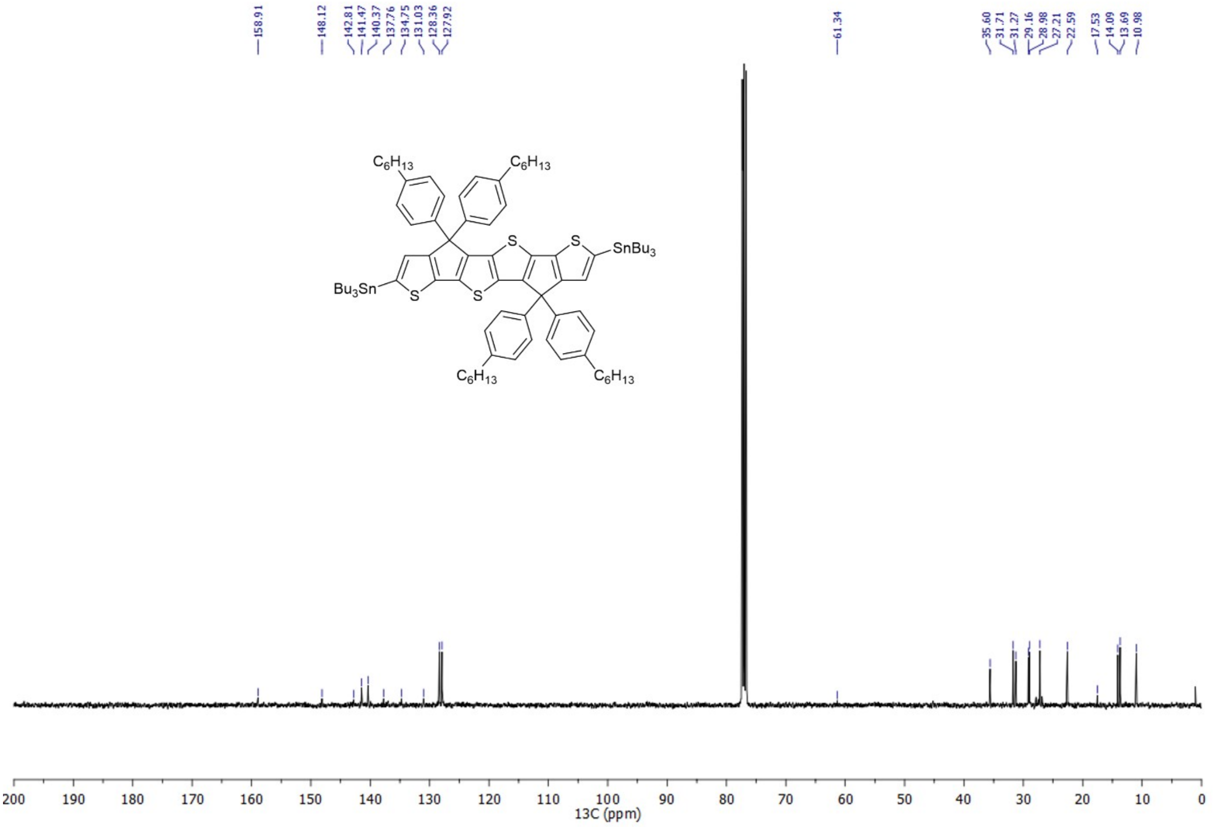

Figure S23. ^{1}H NMR Spectrum of compound 10 in $CDCl_3$.

Figure S24. ^{13}C NMR Spectrum of compound 10 in $CDCl_3$.

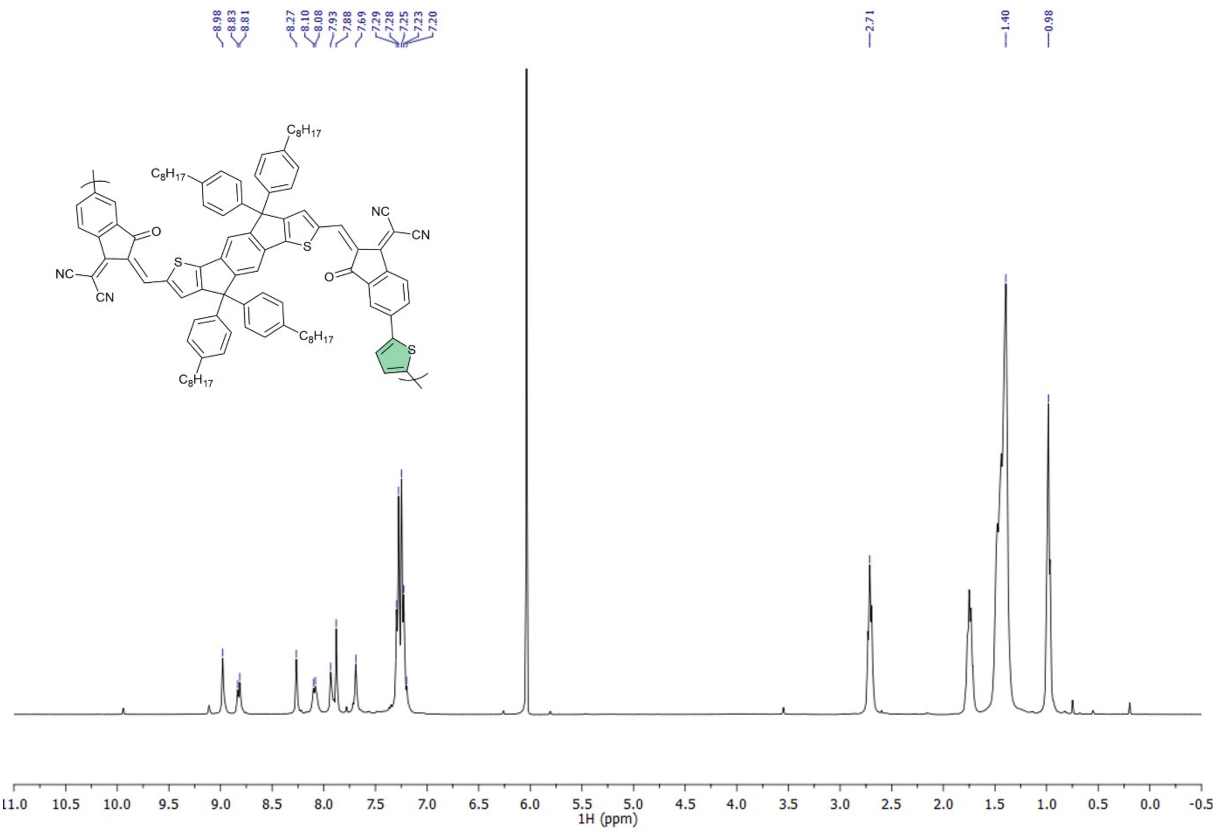

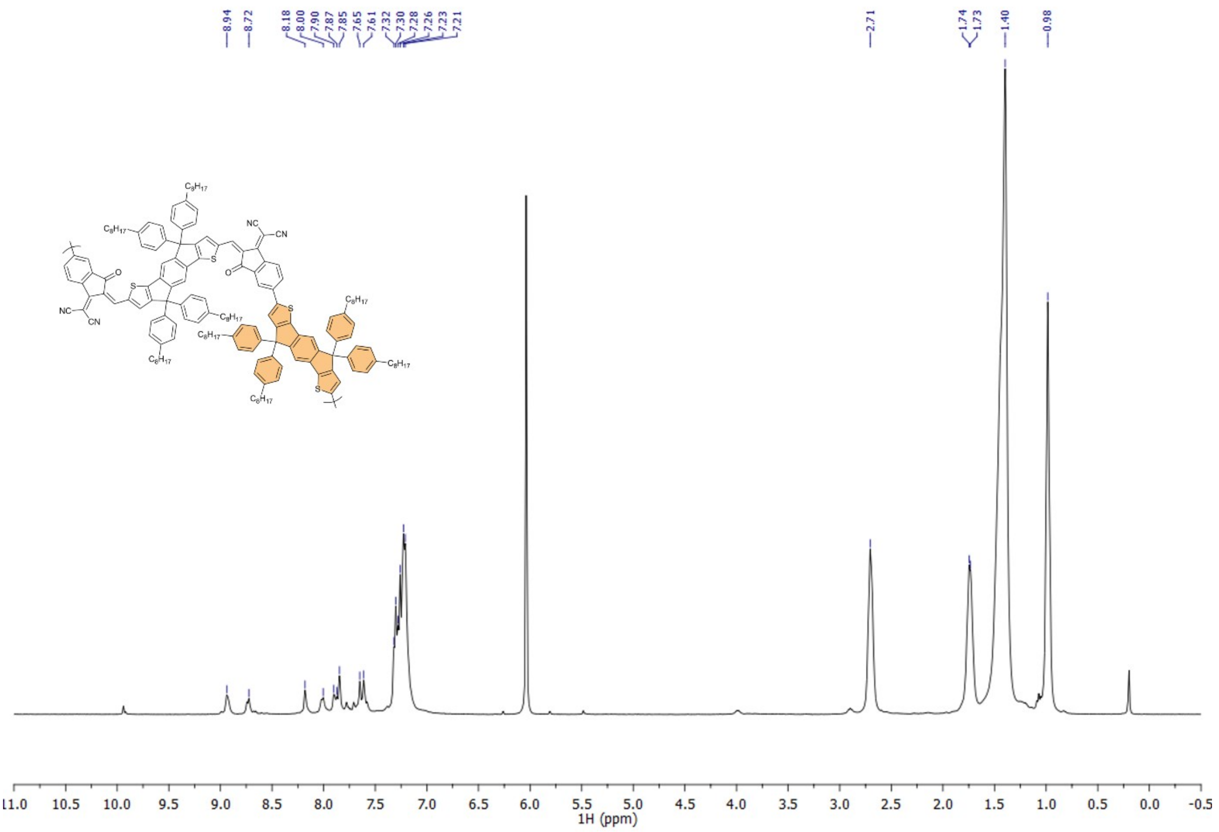

Figure S25. ^1H NMR Spectrum of **LuNi-2** in CD_2Cl_4 at 393K.

Figure S26. ^1H NMR Spectrum of **LuNi-3** in CD_2Cl_4 at 393K.

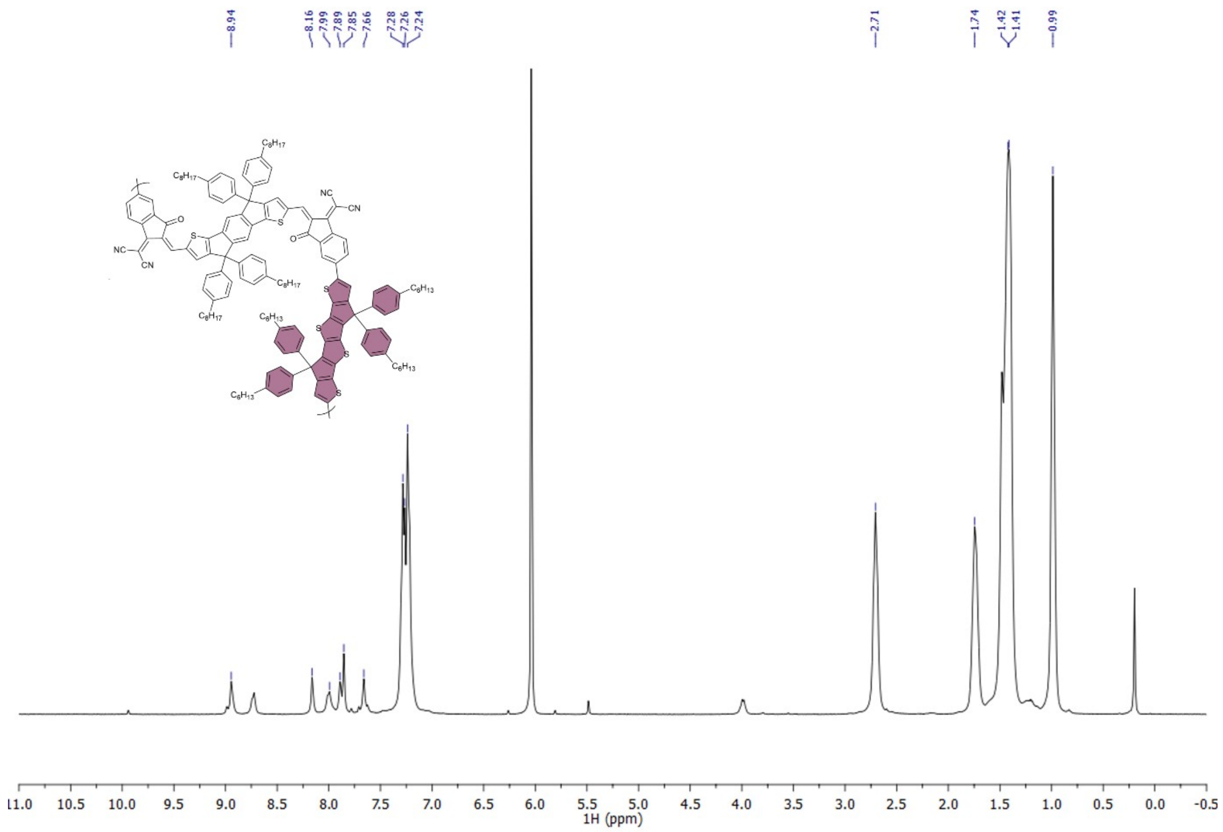

Figure S27. ^{1}H NMR Spectrum of **LuNi-4** in CD_2Cl_4 at 393K.

SIZE-EXCLUSION CHROMATOGRAPHY

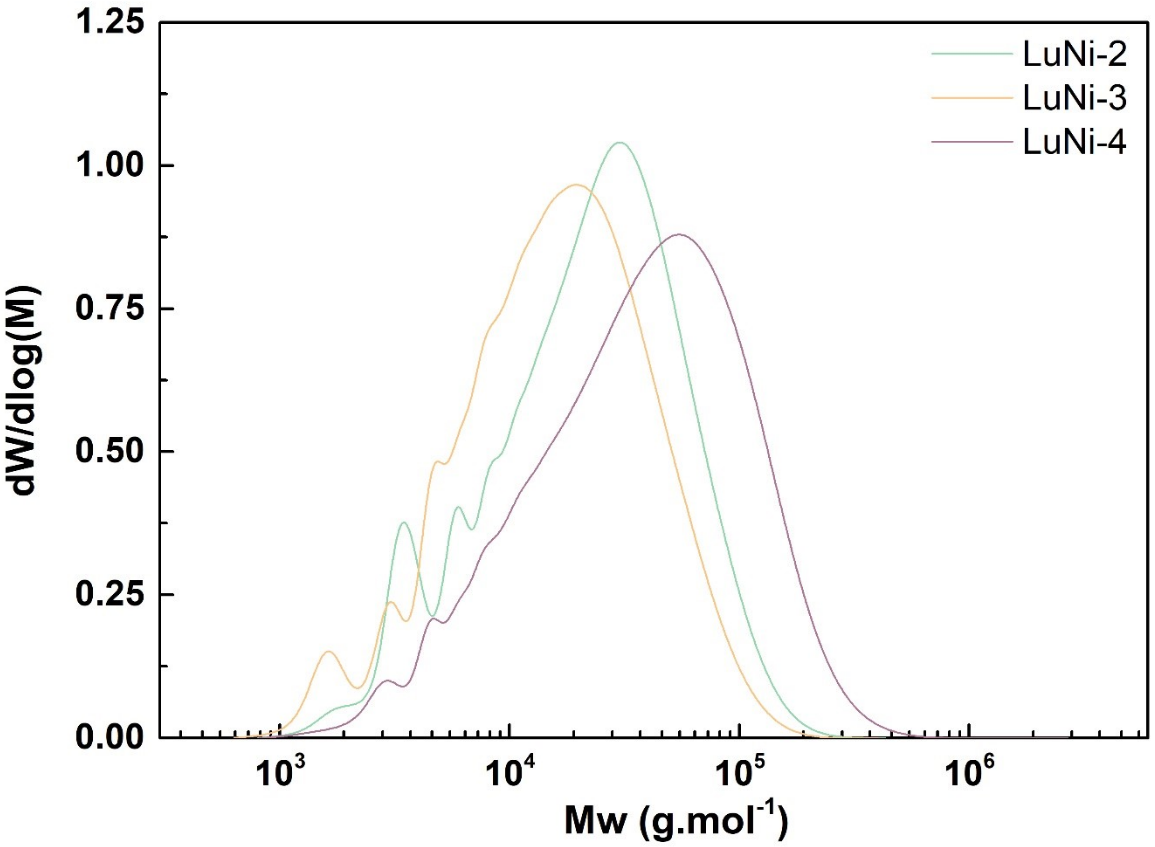

Figure S28. Size exclusion chromatography traces for **LuNi-2** (Mn: 12.7 kg/mol, Mw: 31.4 kg/mol, Đ: 2.5, green), **LuNi-3** (Mn: 9.6 kg/mol, Mw: 23.2 kg/mol, Đ: 2.4, orange), **LuNi-4** (Mn: 19.3 kg/mol, Mw: 57.7 kg/mol, Đ: 3.0, purple).

CYCLIC

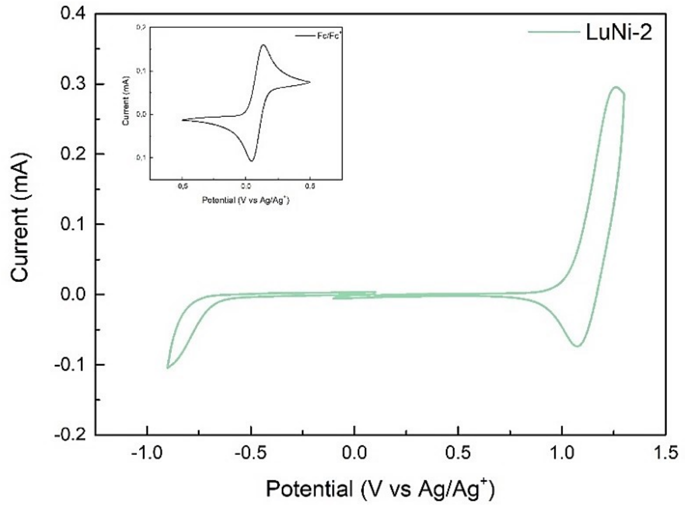

VOLTAMMETRY

Figure S29. Energy levels and electron density of the HOMO and LUMO of **LuNi-2** obtained by cyclic

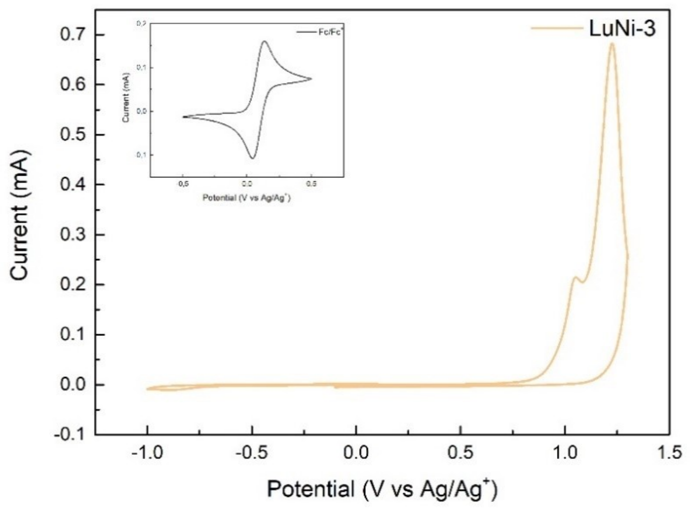

voltammetry.

Figure S30. Energy levels and electron density of the HOMO and LUMO of **LuNi-3** obtained by cyclic voltammetry.

Figure S31. Energy levels and electron density of the HOMO and LUMO of **LuNi-4** obtained by cyclic voltammetry.

DFT CALCULATIONS

Matériau	HOMO (eV)	LUMO (eV)
**LuNi-2**	-5.8	-4.0

HOMO **LUMO**

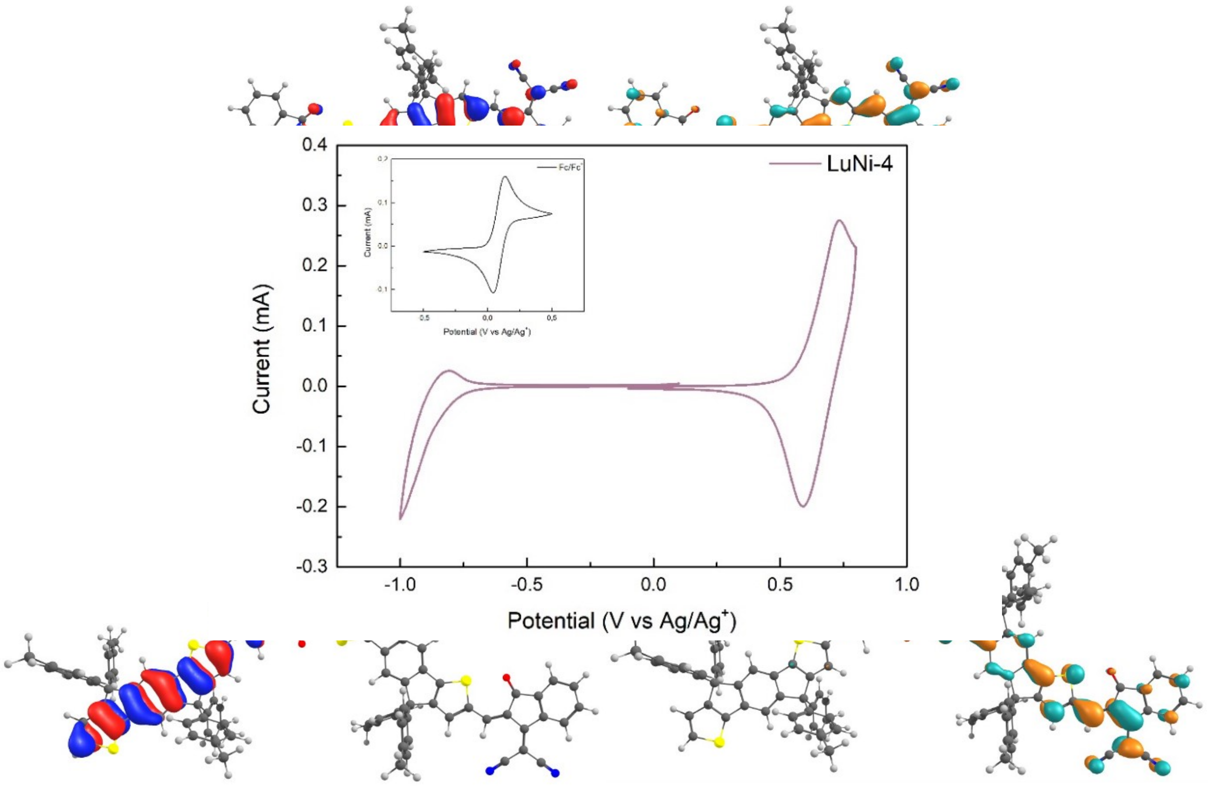

Figure S32. Energy levels and electron density of the HOMO and LUMO of **LuNi-2** obtained by DFT calculations.

Figure S33. Energy levels and electron density of the HOMO and LUMO of **LuNi-3** obtained by DFT calculations.

Matériau	HOMO (eV)	LUMO (eV)
**LuNi-4**	-5.3	-3.9

HOMO **LUMO**

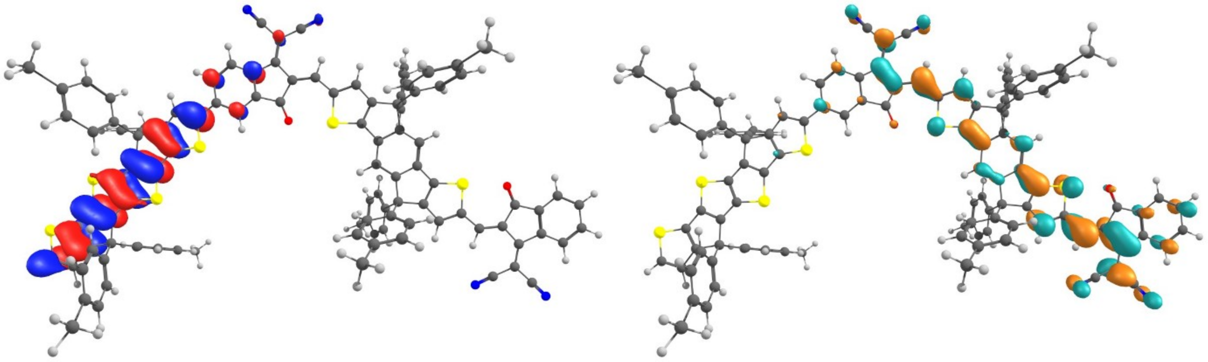

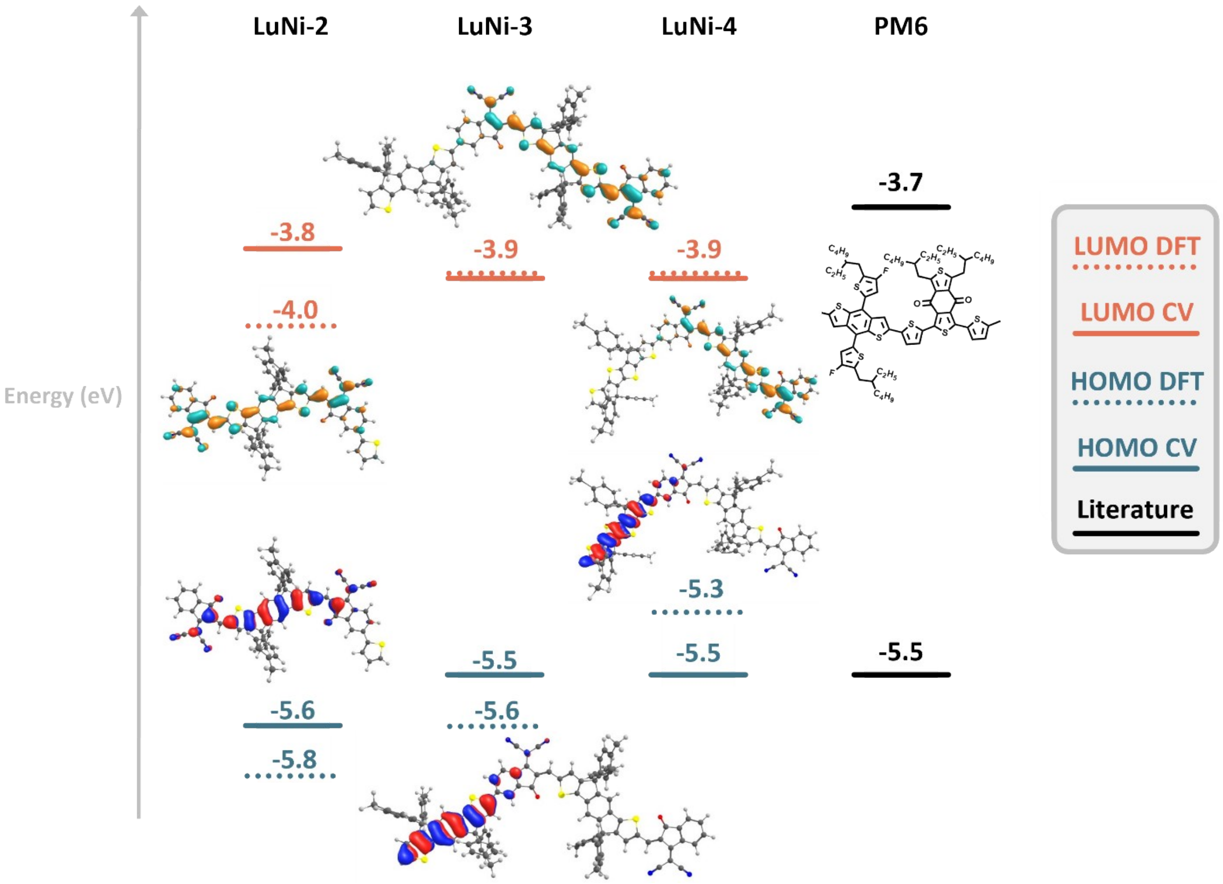

Figure S34. Energy levels and electron density of the HOMO and LUMO of **LuNi-2** obtained by DFT calculations.

Figure S35. Experimental and DFT-calculated energy levels of the frontier orbitals and their spatial localizations for **LuNi-2**, **LuNi-3** and **LuNi-4**, and comparison with energy levels of PM6 extracted from literature.[9]

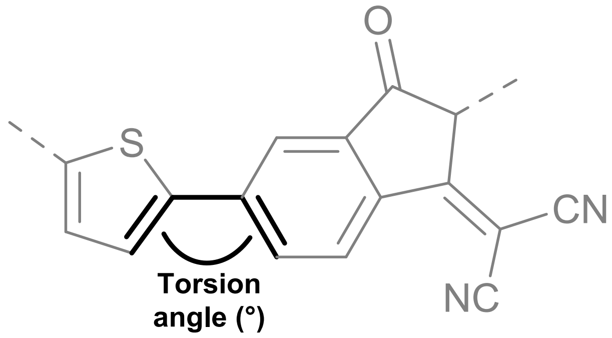

Figure S36. Details of the measurement of torsion angle in all NFA polymers.

UV-Vis SPECTROSCOPY

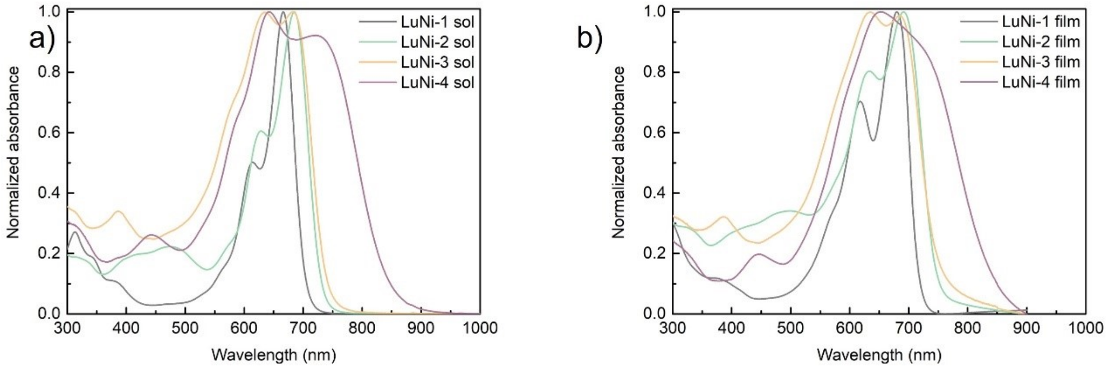

Figure S37. UV-vis spectra in (a) chloroform solution and (b) thin film for monomer **LuNi-1** and three polymers **LuNi-2**, **LuNi-3** and **LuNi-4**.

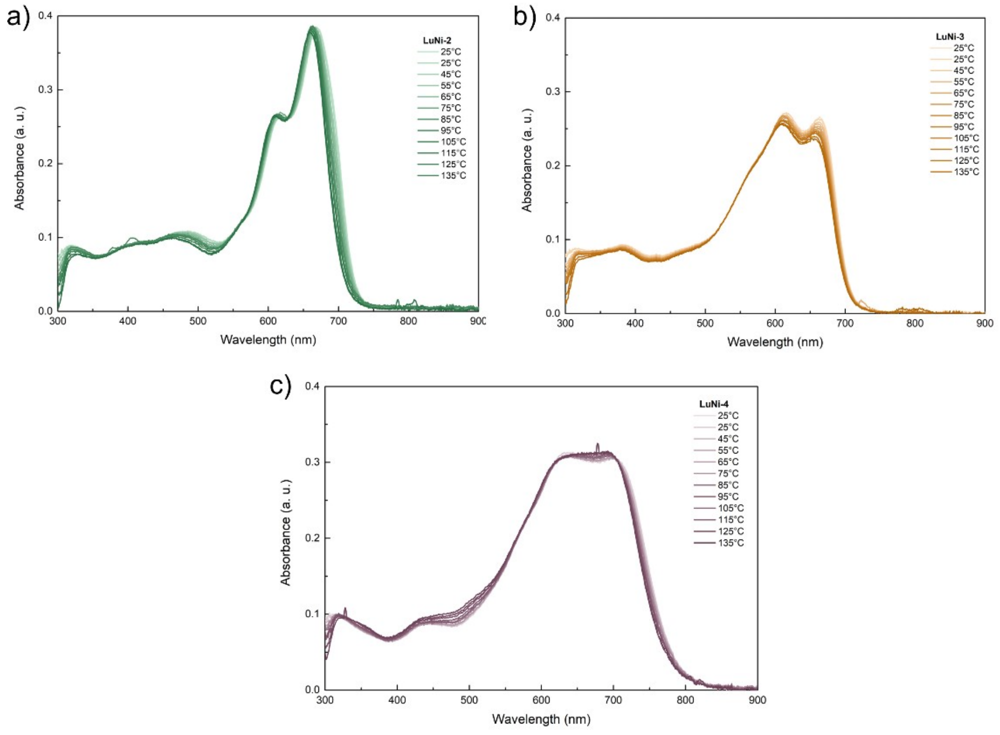

Figure S38. UV-vis spectra in chloroform solution at different temperatures for (a) **LuNi-2**, (b) **LuNi-3** and (c) **LuNi-4**.

GRAZING INCIDENCE WIDE ANGLE X-RAY SCATTERING

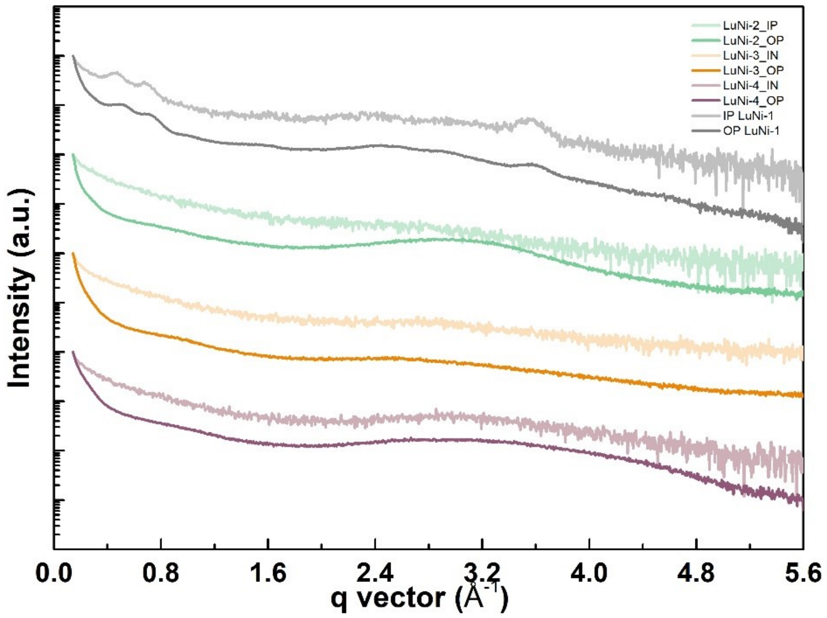

Figure S39. Overlay of GIWAXS profiles of all four materials (grey: LuNi-1, green: LuNi-2, yellow: LuNi--3 and purple: LuNi-4)

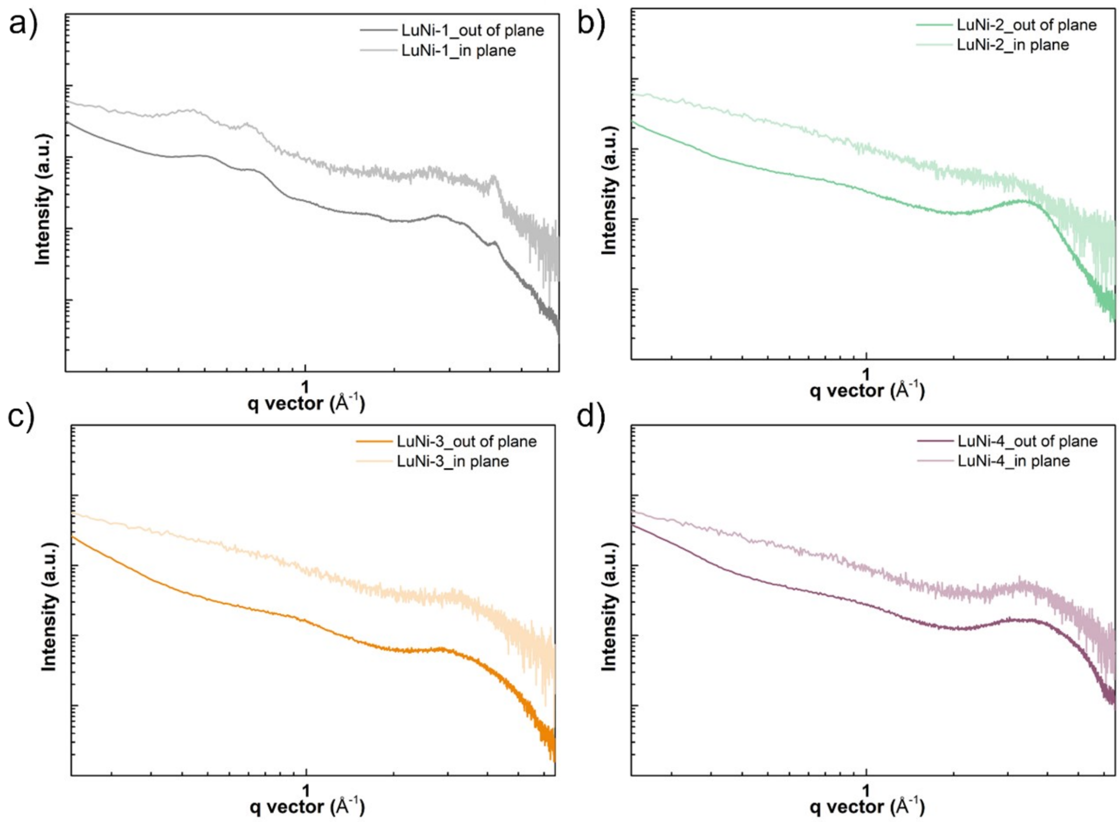

Figure S40. GIWAXS profiles detailled in out of plane and in plane of (a) LuNi-1, (b) **LuNi-2**, (c) **LuNi-3** and (d) **LuNi-4** films from chloroform solutions.

PEAK FORCE MODE ATOMIC FORCE MICROSCOPY

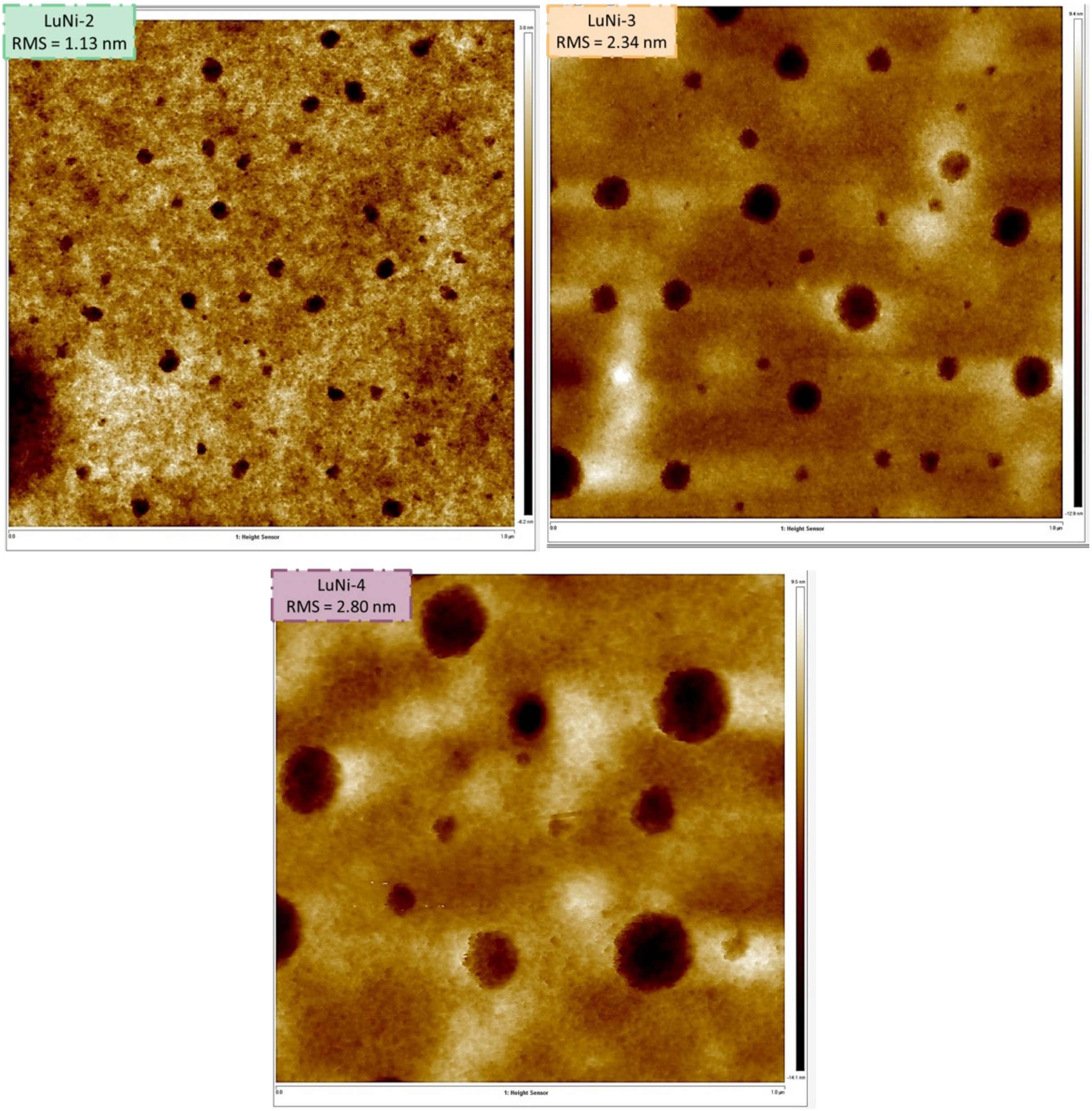

Figure S41. AFM images and RMS of **LuNi-2**, **LuNi-3** and **LuNi-4** films from chloroform solutions.

PHOTOVOLTAIC PERFORMANCES

Device	V_{OC} (V)	J_{SC} (mA·cm-2)	J_{SC} integrated[b] (mA·cm-2)	FF (%)	PCE (%)
**PM6:LuNi-1**	0.977 (0.963±0.016)[a]	1.84 (1.73±0.07)[a]	/	39.62 (38.31±1.54)[a]	0.71 (0.64±0.05)[a]
**PM6:LuNi-2**	0.847 (0.844±0.003)[a]	8.69 (7.74±0.58)[a]	-8.94	35.60 (35.67±0.38)[a]	2.62 (2.33±0.19)[a]
**PM6:LuNi-3**	0.912 (0.906±0.006)[a]	6.22 (5.54±0.46)[a]	-5.36	36.55 (37.36±0.51)[a]	2.07 (1.87±0.14)[a]
**PM6:LuNi-4**	0.843 (0.827±0.019)[a]	1.23 (1.18±0.06)[a]	-1.32	33.02 (31.86±1.14)[a]	0.34 (0.31±0.03)[a]

Table S3. Photovoltaic device parameter of the best pixel for all the polymer solar cells ([a]average of 8 pixels, [b]obtained by IPCE measurements).

IMPEDANCE SPECTROSCOPY

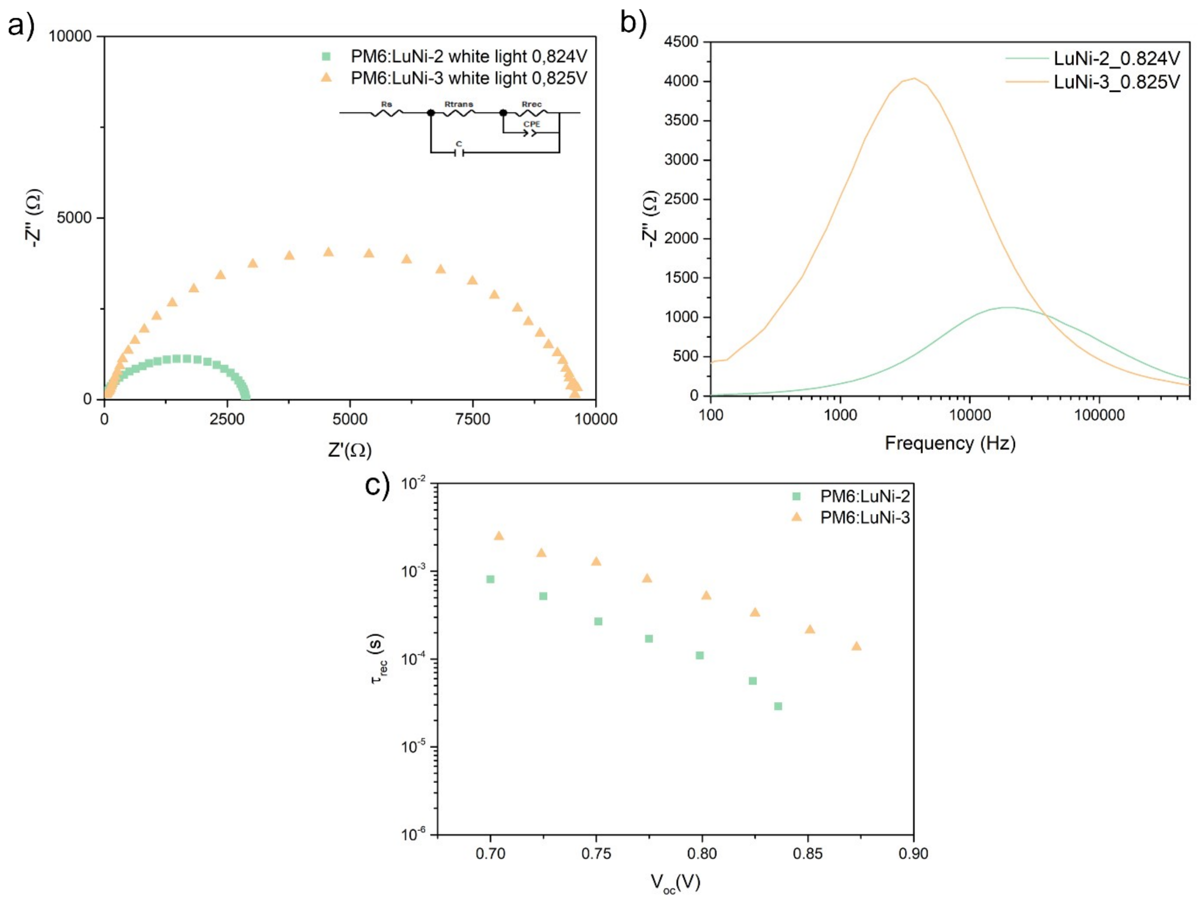

Figure S42. (a) Nyquist (b) Cole-Cole plot and (c) recombination lifetime versus V_{oc} of PM6:**LuNi-2** and PM6:**LuNi-3** devices.

RECOMBINATION KINETICS

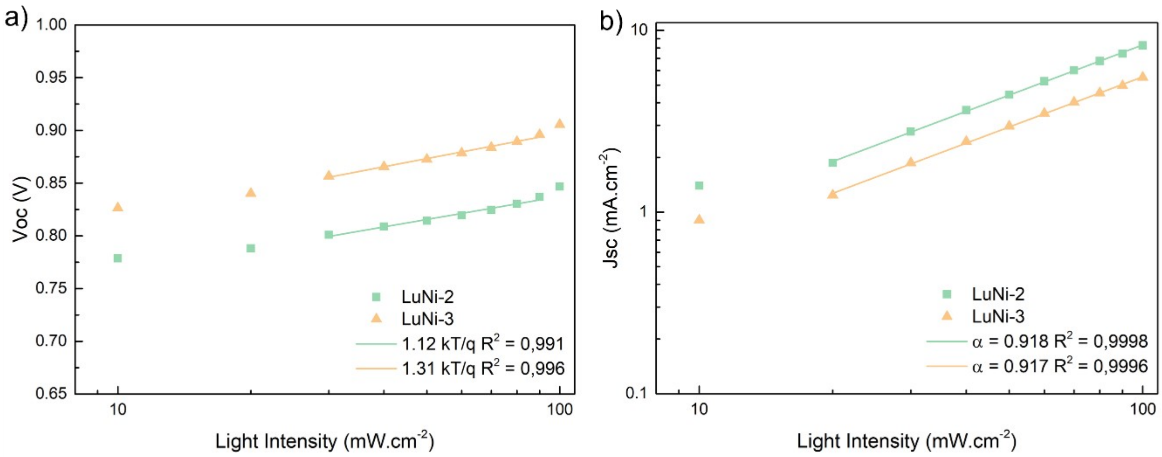

Figure S43. (a) Double logarithmic plot of J_{sc} as a function of the light intensity and fitting line according to the power law. (b) Measurement of V_{oc} versus light intensity for the relevant devices.

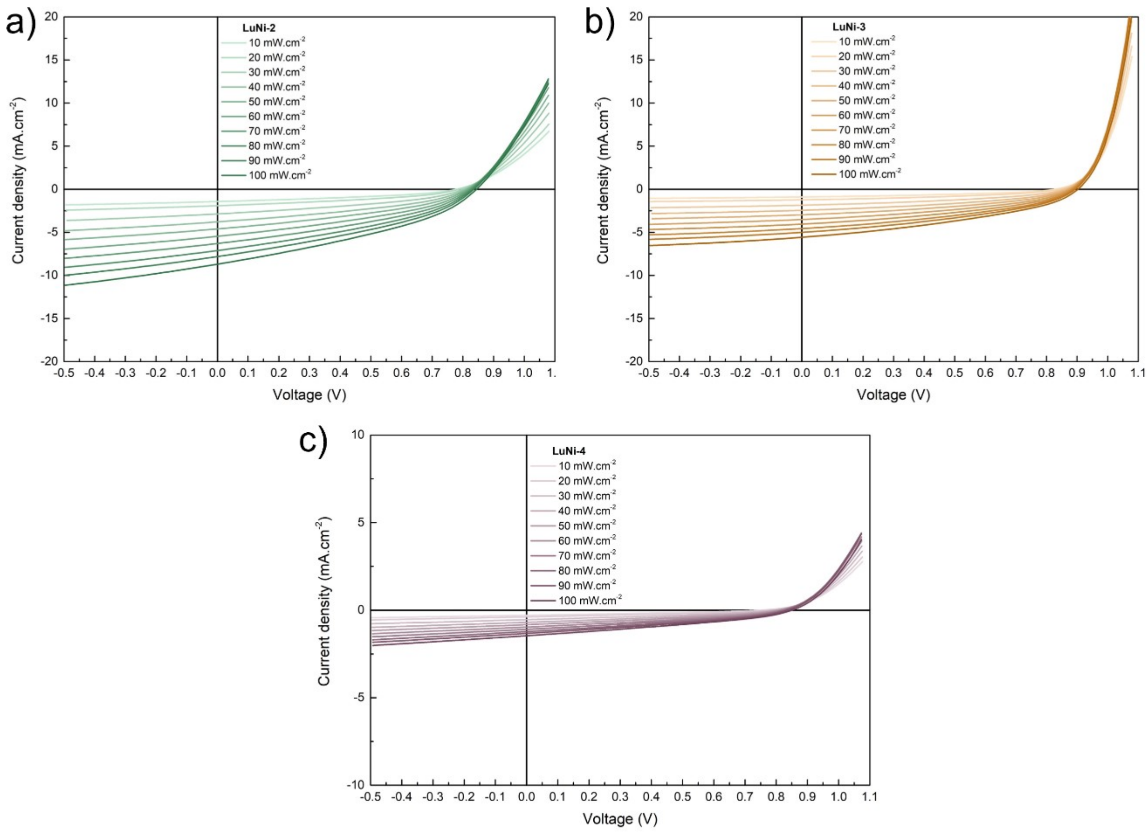

Figure S44. J-V curves for all-polymer solar cells of (a) PM6:**LuNi-2**, (b) PM6:**LuNi-3** and (c) PM6:**LuNi-4** (1:1.5) under different light intensities.

PHOTOLUMINESCENCE

Polymer	Stock shift$_{sol}$ (cm^{-1})	Stock shift$_{film}$ (cm^{-1})	Quenching efficiency (%)^{a}	τ (ns)^{b}	τ$_{blend}$ (ns)^{c}
**LuNi-2**	789	953	82	0.68	0.81
**LuNi-3**	867	961	48	0.55	1.05

Table S4. Parameters Extracted from photoluminescence and absorption curves. [a]The quenching efficiency is calculated by the equation $\Phi q = (I_0 - I)/I_0$, where I_0 is the PL intensity of neat films and I is the PL intensity of blend films. [b,c]The exciton lifetime in solution is calculated according to the literature[10].

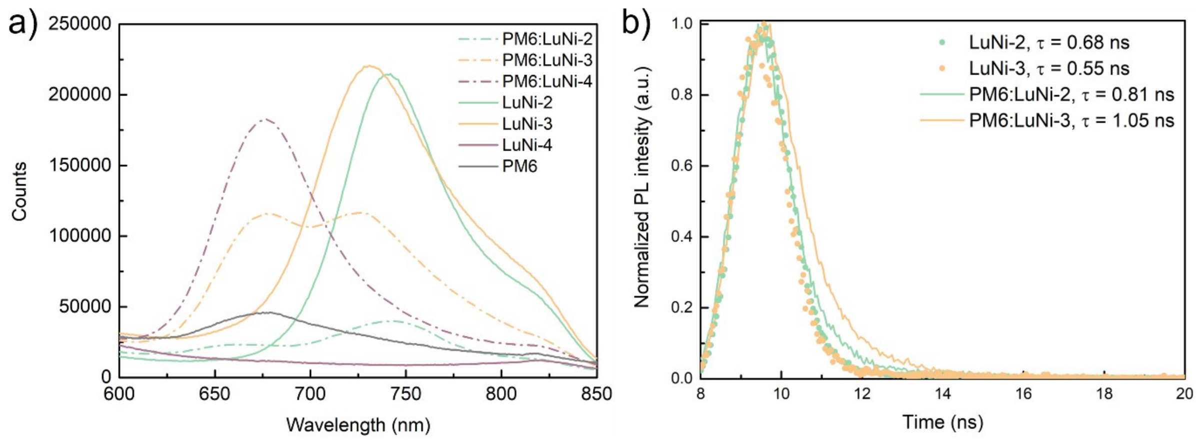

Figure S45. (a) Photoluminescence curves of blend films (dash-dot line) and neat films (continuous line). (b) Time-resolved photoluminescence (TRPL) curves of the neat solutions (scatter plot) and blend solutions (continuous line).

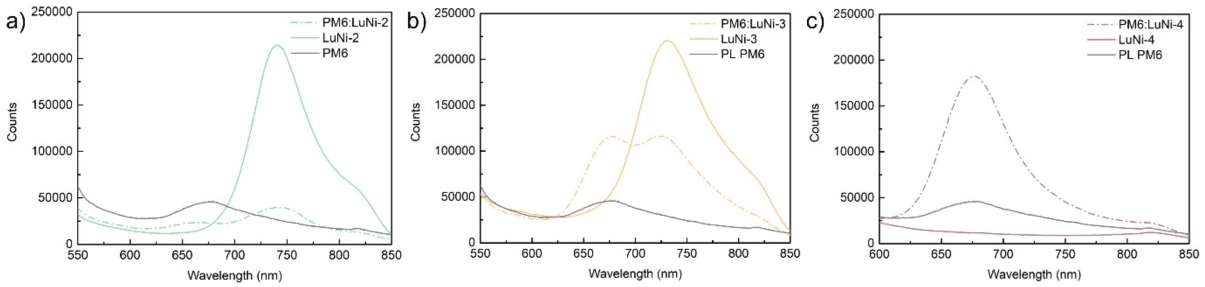

Figure S46. Comparison between the PL in blend film (dash-dot line) and neat film (continuous line) for (a) LuNi-2, (b) LuNi-3 and (c) LuNi-4 materials.

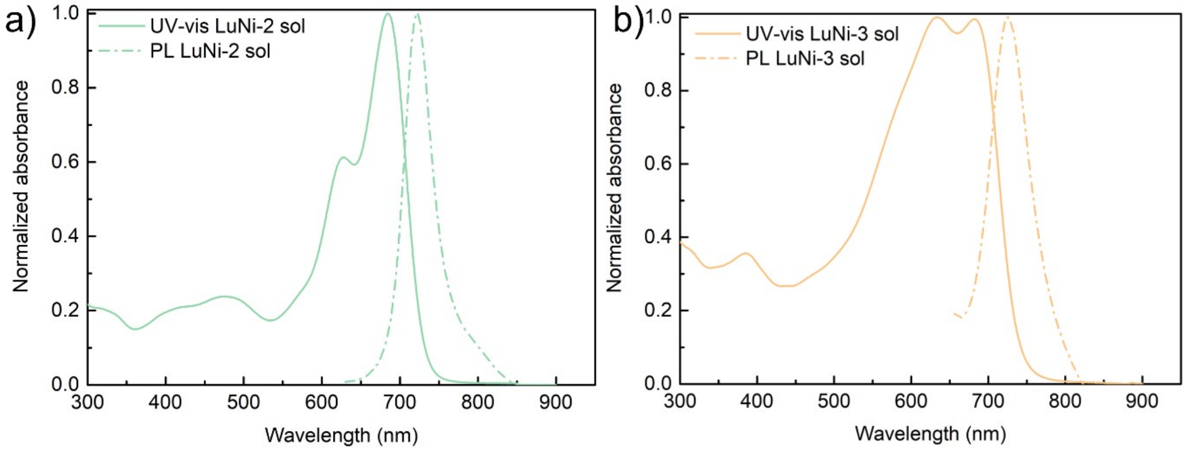

Figure S47. Comparison between UV-vis spectra (continuous line) and PL spectra (dash-dot line) for (a) LuNi-2 and (b) LuNi-3 in chloroform solution.

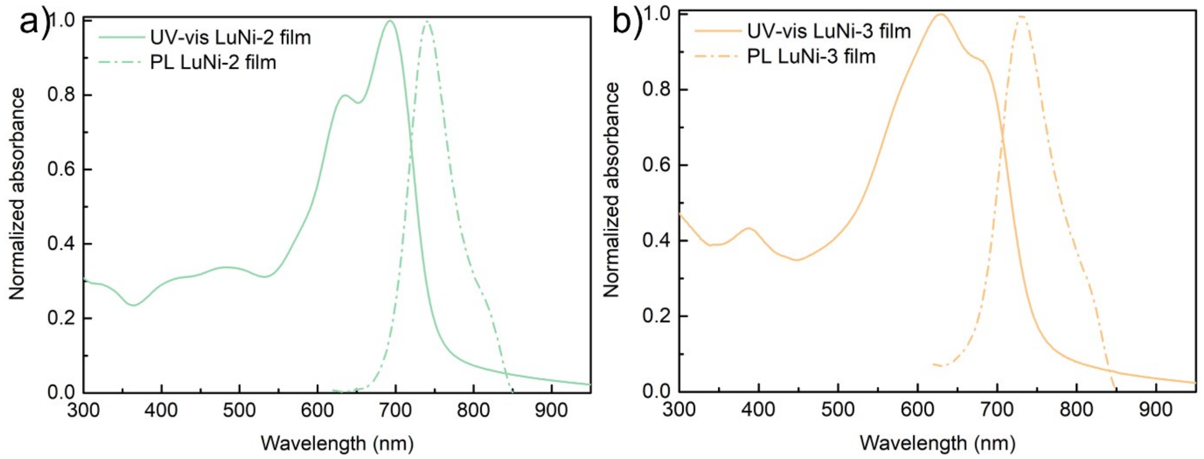

Figure S48. Comparison between UV-vis spectra (continuous line) and PL spectra (dash-dot line) for (a) LuNi-2 and (b) LuNi-3 in tin films.

THERMAL GRAVIMETRIC ANALYSIS

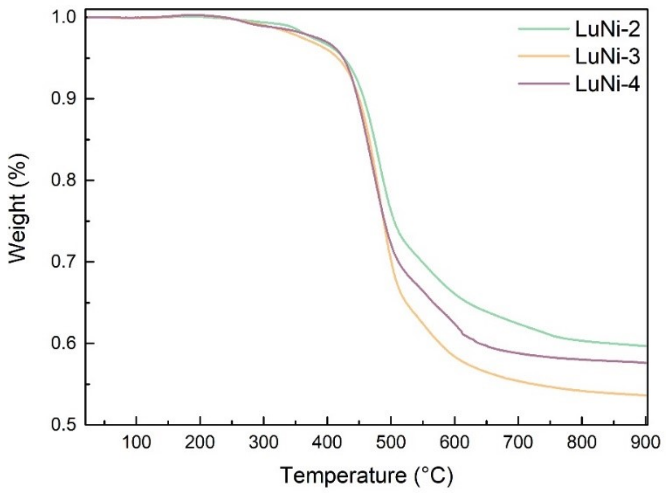

Figure S49. TGA plots of **LuNi-2**, **LuNi-3** and **LuNi-4** at a scan rate of 10 °C.min^{-1} under nitrogen atmosphere.

THERMAL STABILITY

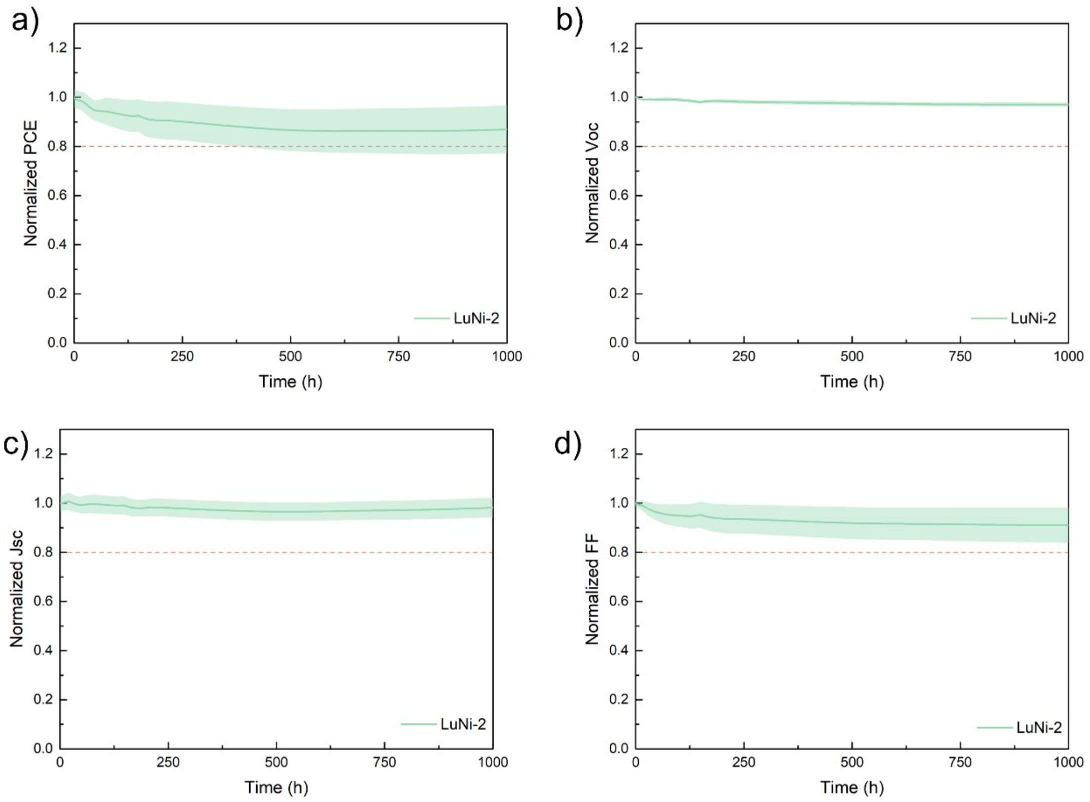

Figure S50. Thermal stability of the devices based on PM6:**LuNi-2** (T_{80} > 2000h). Variation of normalized a) PCE, b) V_{oc}, c) J_{sc} and d) FF parameters.

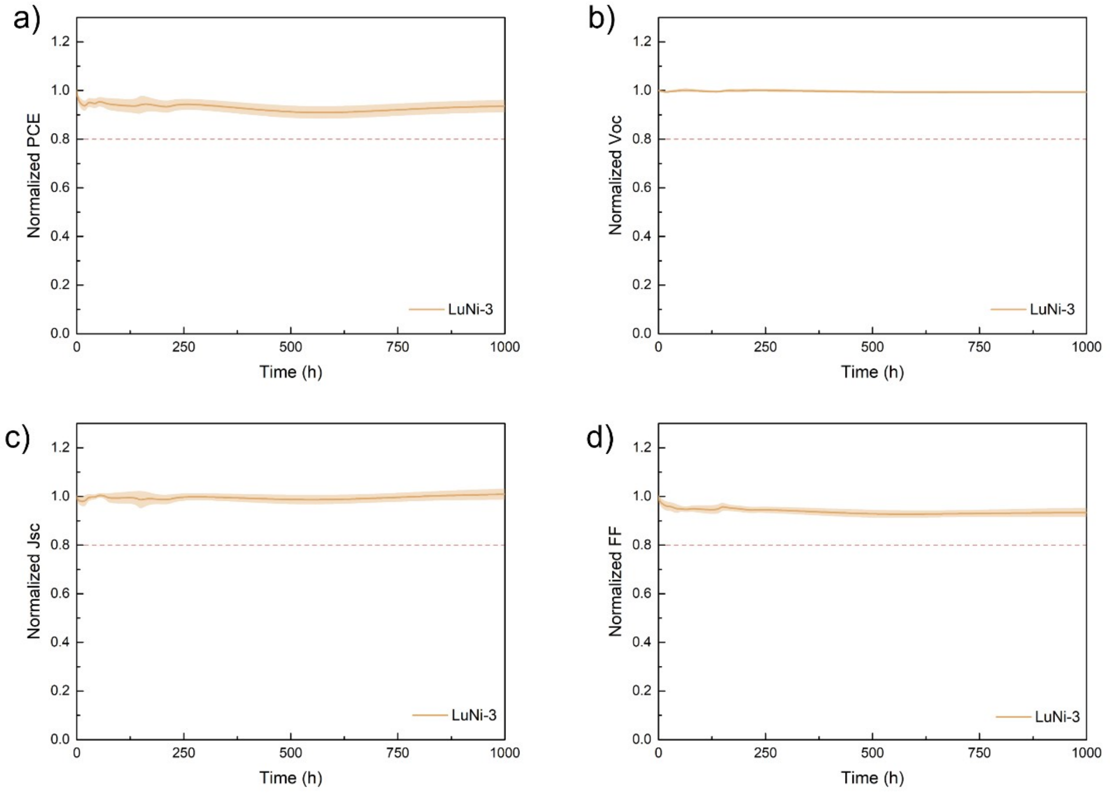

Figure S51. Thermal stability of the devices based on PM6:**LuNi-3**(T_{80} > 2000h). Variation of normalized a) PCE, b) V_{oc}, c) J_{sc} and d) FF parameters

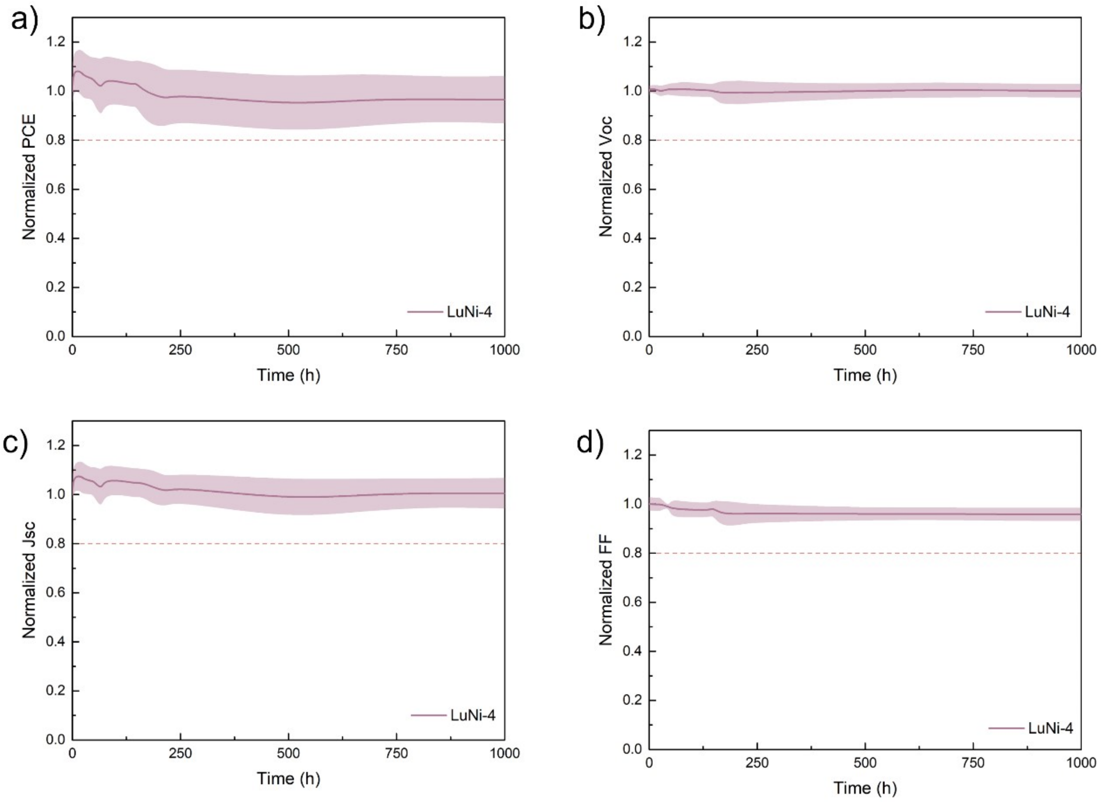

Figure S52. Thermal stability of the devices based on PM6:**LuNi-4**(T_{80} > 2000h). Variation of normalized a) PCE, b) V_{oc}, c) J_{sc} and d) FF parameters.

UNDER ILLUMINATION STABILITY

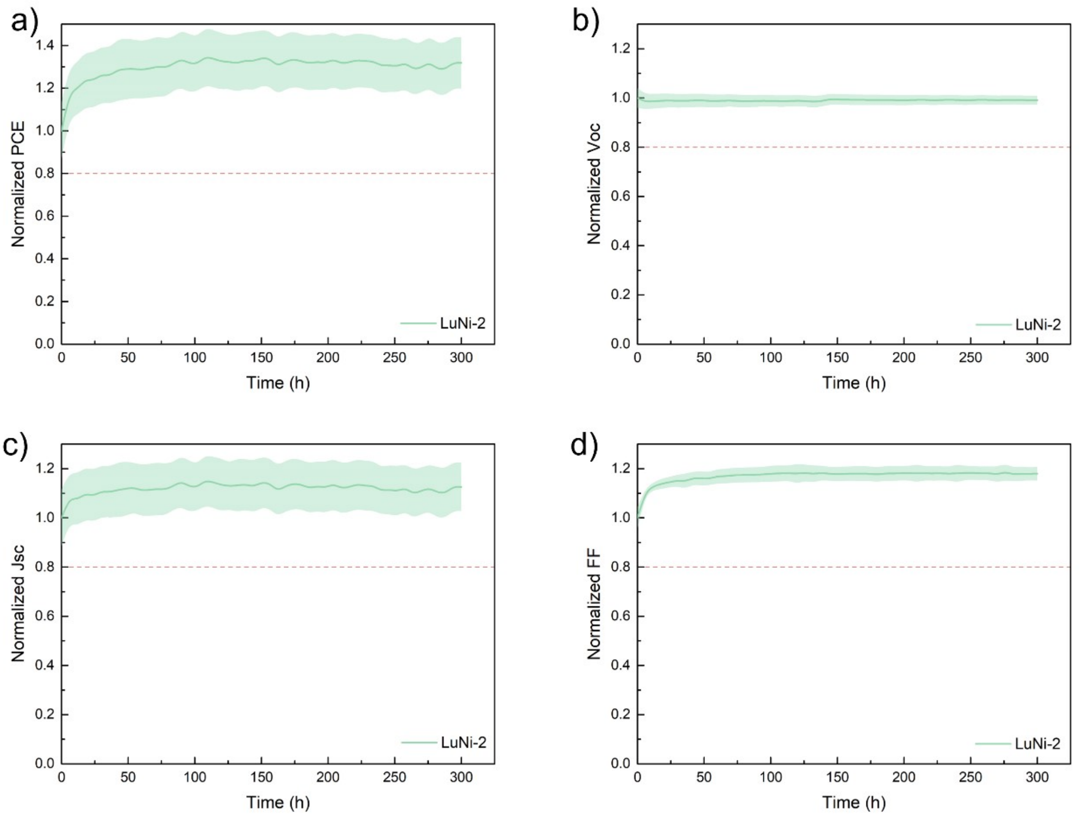

Figure S53. Under illumination stability of the devices based on PM6:**LuNi-2**(T_{80} > 300h). Variation of normalized a) PCE, b) V_{oc}, c) J_{sc} and d) FF parameters.

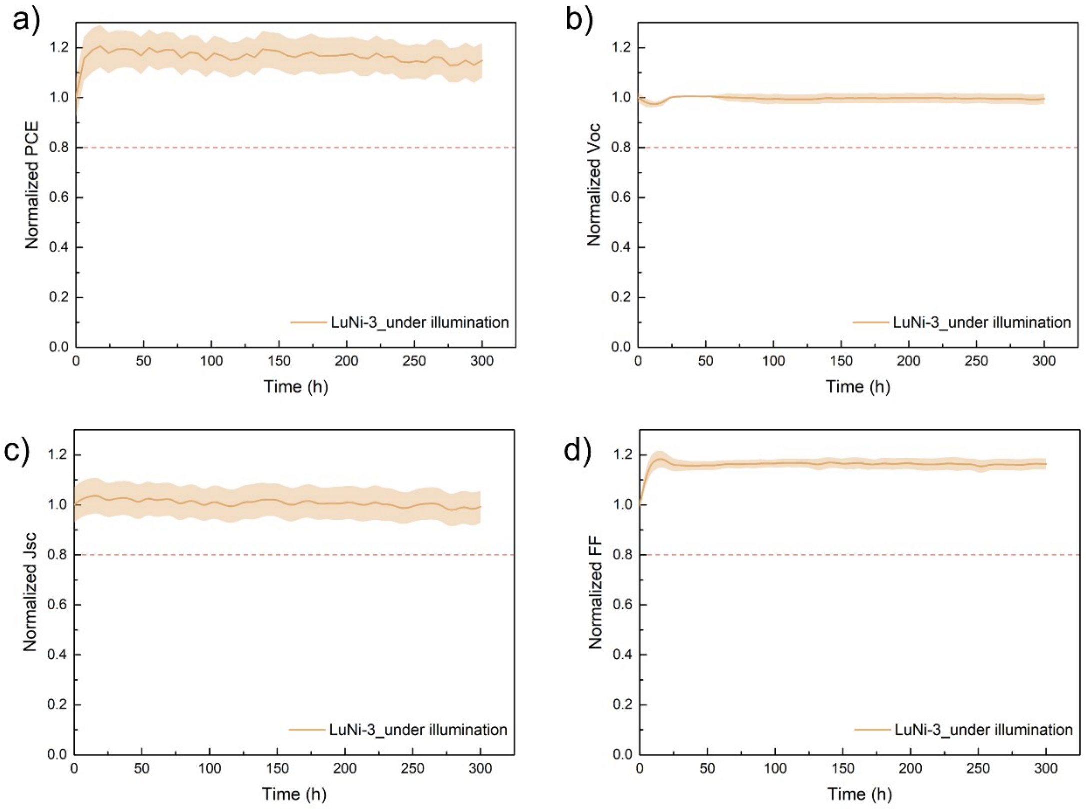

Figure S54. Under illumination stability of the devices based on PM6:**LuNi-3**(T_{80} > 300h). Variation of normalized a) PCE, b) V_{oc}, c) J_{sc} and d) FF parameters.

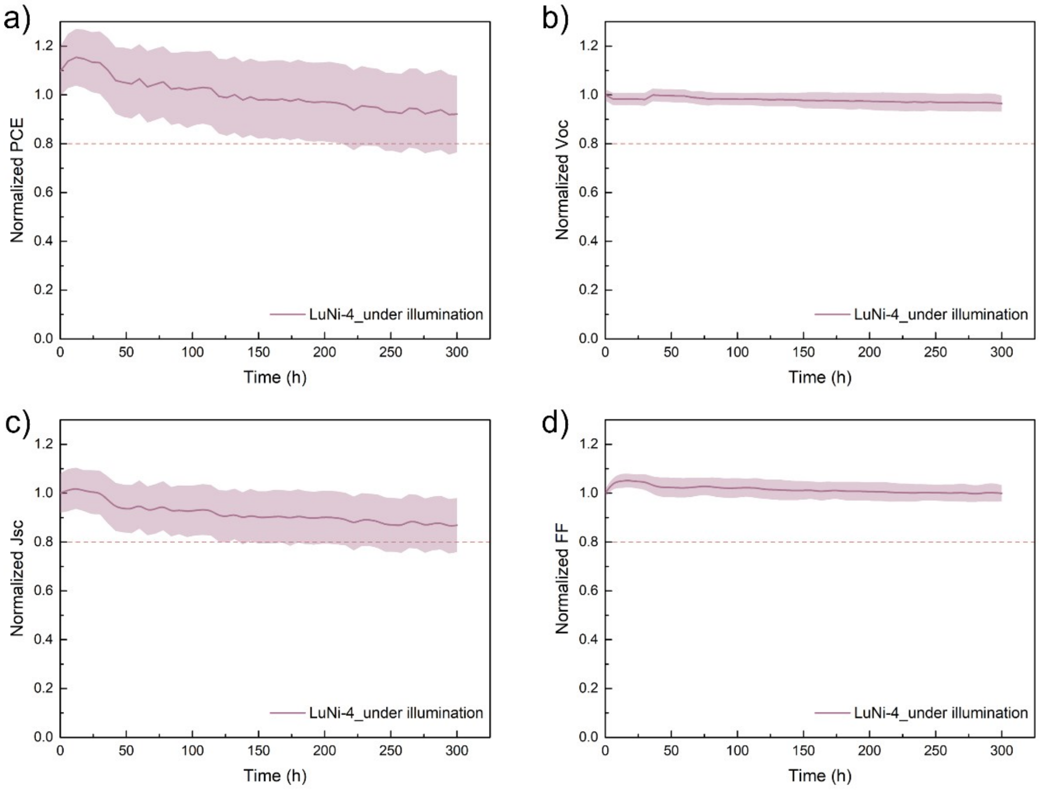

Figure S55. Under illumination stability of the devices based on PM6:**LuNi-4**(T_{80} > 300h). Variation of normalized a) PCE, b) V_{oc}, c) J_{sc} and d) FF parameters.

LITERATURE REVIEW

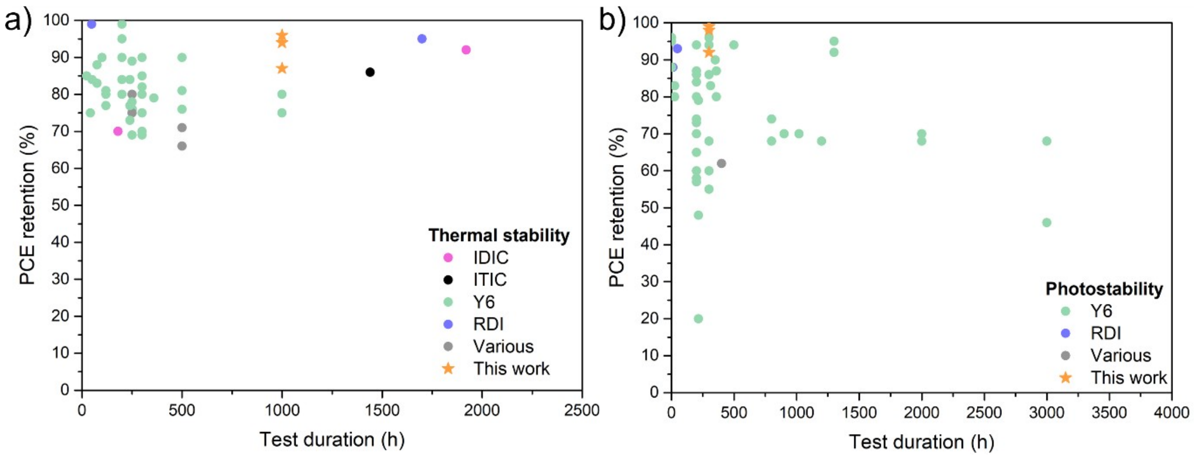

Figure S56. Review of the literature on a) thermal stability and b) photostability of all-polymer solar cells. All data extracted from the literature are detailed in the table S5.

Year	Active Layer	Family	Test duration (h)	PCE initial (%)	PCE retention (%)	Ref
2019	PM6:PZ1	IDIC	1920	11.2	92	[11]
2020	PM6:PF1-TS4	IDIC	180	8.63	70	[12]
2019	PM6:PN1 +3% CN	ITIC	1440	10.5	86	[13]
2020	PM6:PYT1	Y6	252	12.2	76	[14]
2019	PTzBI-Si:N2200	RDI	48	11	99	[15]
2018	PBDB-T:N2200	RDI	1700	6.33	95	[16]
2019	PBDB-T:N2200	RDI	300	6.11	90	[17]
2022	PTB7-Th:DCNBT-TPIC	Various	500	10.11	66	[18]
2022	Si-BDT:DCNBT-TPIC	Various	500	9.92	71	[18]
2021	PBDB-T:PYT	Y6	120	12.9	77	[19]
2021	PBDB-T:PZT	Y6	120	14.5	80	[19]
2021	PBDB-T:PZT-γ	Y6	120	15.8	81	[19]
2024	PM6:PY-V-γ	Y6	1000	17.1	75	[20]
2024	PM6:PffBQx-T	Y6	1000	16.8	80	[20]
2025	PM6:PY-IT	Y6	300	16.84	70	[21]
2025	PM6:PY-DTBT	Y6	300	17.58	75	[21]
2025	PM6:PY-DT-BT	Y6	300	13.24	69	[21]
2024	PM6:PY-DT-X	Y6	360	15.6	79	[22]
2024	PBDB-T:PY-IT	Y6	75	15.05	83	[23]
2024	QQ1:PY-IT	Y6	75	18.81	88	[23]
2024	PBDB-T:P1	Various	250	8.12	80	[24]
2024	PBDB-T:P2	Various	250	9.33	75	[24]
2024	D18:PY-IT	Y6	200	16.14	84	[25]
2021	PBDB-T:PYTT-1	Y6	200	13.54	90	[26]
2021	PBDB-T:PYTT-2	Y6	200	14.32	99	[26]
2021	PBDB-T:PYTT-3	Y6	200	12.41	80	[26]
2025	PM6:PY-V-γ	Y6	500	17.31	81	[27]
2025	PM6:PY-EY-5	Y6	500	18.45	90	[27]
2025	PM6:PY-EY-20	Y6	500	15.15	76	[27]
2020	PBDB-T:PF5-Y5	Y6	42	12.52	75	[28]
2021	PBDB-T:P(BDT2BOY5-Cl)	Y6	100	10.67	90	[29]
2022	PM6:PY-V-γ	Y6	240	17.1	84	[30]
2022	PM6:PY-T-γ	Y6	240	16.1	77	[30]
2022	PM6:PY-2T-γ	Y6	240	15.3	73	[30]
2021	PM6:PYF-T	Y6	200	13.77	95	[31]
2021	PM6:PY-T	Y6	200	10.74	90	[31]
2021	PM6:PYF-T	Y6	250	14	78	[32]
2021	PM6:PYF-T-o	Y6	250	15.2	89	[32]
2021	PM6:PYF-T-m	Y6	250	1.4	69	[32]
2022	PBDB-T:PYT	Y6	200	13.59	90	[33]
2022	PBDB-T:PYT-Tz	Y6	200	15.1	90	[33]
2022	PM6:PYT-1S1Se	Y6	50	16.3	84	[34]
2022	PBDB-T:PZT-C12	Y6	300	13.1	80	[35]
2022	PBDB-T:PZT-C8	Y6	300	13.8	82	[35]
2022	PBDB-T:PZT-C1	Y6	300	14.9	85	[35]
2021	PM6:PY-IT	Y6	24	15.53	85	[36]
2023	PM6:PY-IT	Y6	300	16.04	90	[37]
**2025**	**PM6:LuNi-2**	**IDIC**	**1000**	**2.62**	**87**	**This work**
**2025**	**PM6:LuNi-3**	**IDIC**	**1000**	**2.07**	**84**	**This work**
**2025**	**PM6:LuNi-4**	**IDIC**	**1000**	**0.34**	**96**	**This work**

Table S5. Literature summary of thermal stability in all-polymer solar cells.

Year	Active Layer	Family	Test duration (h)	PCE initial (%)	PCE retention (%)	Ref
2019	PTzBI-Si:N2200	RDI	48	11	93	[38]
2017	PTzBI:N2200	RDI	12	8.96	88	[39]
2021	PBDB-T:DCNBT-TPC	Various	400	10.5	62	[40]
2021	PTB7-Th:DCNBT-TPC	Various	400	9.4	62	[40]
2021	JD40:PJTET	Y6	1300	10.93	92	[41]
2021	JD40:PJTVT	Y6	1300	16.13	95	[41]
2025	PM6:PY-IT	Y6	300	16.84	60	[42]
2025	PM6:PY-DTBT	Y6	300	17.58	68	[42]
2025	PM6:PY-DT-BT	Y6	300	13.24	55	[42]
2025	PM6:PY-IT	Y6	800	17.14	68	[43]
2025	PBZ-10:PY-IT	Y6	800	19.06	74	[43]
2023	PM6:PY-IT	Y6	1200	15.17	68	[44]
2024	PBDB-T:PY-IT	Y6	25	15.05	80	[45]
2024	QQ1:PY-IT	Y6	25	18.81	83	[45]
2021	PM6:PYT	Y6	2000	14.5	68	[46]
2021	PM6:PY2F-T	Y6	2000	15	70	[46]
2024	PM6:PY-V-γ	Y6	200	17	58	[47]
2024	PM6:PYO-V	Y6	200	12.8	60	[47]
2021	PBDB-T:PYTT-1	Y6	200	13.54	70	[48]
2021	PBDB-T:PYTT-2	Y6	200	14.32	80	[48]
2021	PBDB-T:PYTT-3	Y6	200	12.41	70	[48]
2021	PBDB-T:PY-O	Y6	216	9.8	20	[49]
2021	PBDB-T:PY-S	Y6	216	14.16	48	[49]
2021	PBDB-T:PY-Se	Y6	216	15.48	79	[49]
2022	PM6:PY-V-γ	Y6	0.5	17.1	96	[50]
2022	PM6:PY-T-γ	Y6	0.5	16.1	96	[50]
2022	PM6:PY-2T-γ	Y6	0.5	15.3	95	[50]
2023	PM6:PY-1S1Se	Y6	3000	16.5	46	[51]
2023	PM6:PY-2Cl	Y6	3000	14.8	68	[51]
2022	PBQx-H-TF:PBTIC-γ-TSe	Y6	900	13.91	70	[52]
2022	PBDB-T:PYT	Y6	200	13.59	57	[53]
2022	PBDB-T:PYT-Tz	Y6	200	15.1	73	[53]
2023	PM6:PYSSe-V	Y6	200	17.03	65	[54]
2023	PM6:PY-Cl	Y6	200	16.37	74	[54]
2022	PM6:PG-IT2F	Y6	500	17.24	94	[55]
2020	PBDB-T:PTPBT-ET0.3	Y6	312	12.52	83	[56]
2022	PM6:pBTICgama-OD	Y6	1020	12.15	70	[57]
2022	PM6:OY1	Y6	300	13.83	94	[58]
2022	PM6:OY3	Y6	300	15.55	96	[58]
2022	PM6:POY	Y6	300	14.16	86	[58]
2022	PBDB-T:OY1	Y6	200	13.84	84	[58]
2022	PBDB-T:OY3	Y6	200	14.87	94	[58]
2022	PBDB-T:POY	Y6	200	13.79	87	[58]
2021	PM6:PY-IT	Y6	350	15.53	90	[59]
2022	PBQ5:PY-IT	Y6	360	15.56	80	[60]
2022	PBQ8:PY-IT	Y6	360	17.04	87	[60]
2023	PM6:PY-IT	Y6	2	16.04	88	[61]
2024	PM6:PY-IT	Y6	200	14.13	87	[62]
2024	PM6:PYF-T-o	Y6	200	13.48	87	[62]
2024	PM6:PY-V-γ	Y6	200	15.9	86	[62]
**2025**	**PM6:LuNi-2**	**IDIC**	**300**	**2.62**	**99**	**This work**
**2025**	**PM6:LuNi-3**	**IDIC**	**300**	**2.07**	**98**	**This work**
**2025**	**PM6:LuNi-4**	**IDIC**	**300**	**0.34**	**92**	**This work**

Table S6. Literature summary of photostability in all-polymer solar cells.

REFERENCES

1 E. Jayaraman, K. Wang, J. Lamminaho, C. Y. Ho, J. W. Andreasen and M. Madsen, *APL Energy*, 2025, **3**, 036101.

2 J. Wachsmuth, A. Distler, C. Liu, T. Heumüller, Y. Liu, C. M. Aitchison, A. Hauser, M. Rossier, A. Robitaille, M.-A. Llobel, P.-O. Morin, A. Thepaut, C. Arrive, I. McCulloch, Y. Zhou, C. J. Brabec and H.-J. Egelhaaf, *Solar RRL*, 2023, **7**, 2300602.

3 J. RAULT-BERTHELOT, L. ANGELY, J. DELAUNAY and J. SIMONET, *New j. chem. (1987)*, 1987, **11**, 487–494.

4 Q. Sun, H. Wang, C. Yang and Y. Li, *J. Mater. Chem.*, 2003, **13**, 800–806.

5 B. C. Thompson, Y.-G. Kim and J. R. Reynolds, *Macromolecules*, 2005, **38**, 5359–5362.

6 A. J. Bard, L. R. Faulkner and H. S. White, *Electrochemical Methods: Fundamentals and Applications*, John Wiley & Sons, 2022.

7 J. Pommerehne, H. Vestweber, W. Guss, R. F. Mahrt, H. Bässler, M. Porsch and J. Daub, *Advanced Materials*, 1995, **7**, 551–554.

8 C. A. Tolman, W. C. Seidel and L. W. Gosser, *J. Am. Chem. Soc.*, 1974, **96**, 53–60.

9 M. Zhang, X. Guo, W. Ma, H. Ade and J. Hou, *Advanced Materials*, 2015, **27**, 4655–4660.

10 E. Fišerová and M. Kubala, *Journal of Luminescence*, 2012, **132**, 2059–2064.

11 Y. Meng, J. Wu, X. Guo, W. Su, L. Zhu, J. Fang, Z.-G. Zhang, F. Liu, M. Zhang, T. P. Russell and Y. Li, *Sci. China Chem.*, 2019, **62**, 845–850.

12 Q. Fan, W. Su, S. Chen, T. Liu, W. Zhuang, R. Ma, X. Wen, Z. Yin, Z. Luo, X. Guo, L. Hou, K. Moth-Poulsen, Y. Li, Z. Zhang, C. Yang, D. Yu, H. Yan, M. Zhang and E. Wang, *Angewandte Chemie International Edition*, 2020, **59**, 19835–19840.

13 J. Wu, Y. Meng, X. Guo, L. Zhu, F. Liu and M. Zhang, *J. Mater. Chem. A*, 2019, **7**, 16190–16196.

14 Q. Wu, W. Wang, T. Wang, R. Sun, J. Guo, Y. Wu, X. Jiao, C. J. Brabec, Y. Li and J. Min, *Sci. China Chem.*, 2020, **63**, 1449–1460.

15 Z. Li, L. Ying, P. Zhu, W. Zhong, N. Li, F. Liu, F. Huang and Y. Cao, *Energy Environ. Sci.*, 2019, **12**, 157–163.

16 Y. Zhang, Y. Xu, M. J. Ford, F. Li, J. Sun, X. Ling, Y. Wang, J. Gu, J. Yuan and W. Ma, *Advanced Energy Materials*, 2018, **8**, 1800029.

17 Y. Xu, J. Yuan, S. Zhou, M. Seifrid, L. Ying, B. Li, F. Huang, G. C. Bazan and W. Ma, *Adv. Funct. Mater.*, 2019, **29**, 1806747.

18 T. Gokulnath, K. Feng, H.-Y. Park, Y. Do, H. Park, R. D. Gayathri, S. S. Reddy, J. Kim, X. Guo, J. Yoon and S.-H. Jin, *ACS Appl. Mater. Interfaces*, 2022, **14**, 11211–11221.

19 H. Fu, Y. Li, J. Yu, Z. Wu, Q. Fan, F. Lin, H. Y. Woo, F. Gao, Z. Zhu and A. K.-Y. Jen, *J. Am. Chem. Soc.*, 2021, **143**, 2665–2670.

20 H. Yu, Y. Wang, C. H. Kwok, R. Zhou, Z. Yao, S. Mukherjee, A. Sergeev, H. Hu, Y. Fu, H. M. Ng, L. Chen, D. Zhang, D. Zhao, Z. Zheng, X. Lu, H. Yin, K. S. Wong, H. Ade, C. Zhang, Z. Zhu and H. Yan, *Joule*, 2024, **8**, 2304–2324.

21 H.-R. Bai, H. Zhang, H. Meng, Y. Li, X. Xu, M.-Q. Liu, Y. Chen, Z.-F. Yao, H.-F. Zhi, A. Mahmood, Y. Wang, J.-H. Ye, M. Jiang, Q. An, H. Y. Woo, H. Wu, Q. Peng and J.-L. Wang, *Materials Science and Engineering: R: Reports*, 2025, **163**, 100916.

22 J. Song, C. Li, H. Ma, B. Han, Q. Wang, X. Wang, D. Wei, L. Bu, R. Yang, H. Yan and Y. Sun, *Advanced Materials*, 2024, **36**, 2406922.

23 Z. Wang, X. Wang, L. Tu, H. Wang, M. Du, T. Dai, Q. Guo, Y. Shi and E. Zhou, *Angewandte Chemie International Edition*, 2024, **63**, e202319755.

24 R. Li, Y. Xu, C. Wang, L. Miao, H. Fang, S. Liang, B. Liu and W. Li, *ACS Appl. Polym. Mater.*, 2024, **6**, 5021–5027.

25 Y. Meng, L. Tang, M. Xiao, W. Zhou, N. Li, J. Jia, T. Jia, W. Su, Z. Bi, W. Peng, B. Fan, A. K.-Y. Jen, W. Ma and Q. Fan, *Chinese Journal of Chemistry*, 2024, **42**, 3559–3566.

26 T. Wang, R. Sun, W. Wang, H. Li, Y. Wu and J. Min, *Chem. Mater.*, 2021, **33**, 761–773.
27 H.-Y. Shi, L. Xing, M.-X. Chen, Z.-Y. Chen, M.-W. Deng, L.-Y. Xu, X.-H. Wu, X.-R. Yang, Y.-M. Shao, E. D. Papkovskaya, Y. Luponosov, R. Sun and J. Min, *Chin J Polym Sci*, 2025, **43**, 1516–1526.
28 Q. Fan, Q. An, Y. Lin, Y. Xia, Q. Li, M. Zhang, W. Su, W. Peng, C. Zhang, F. Liu, L. Hou, W. Zhu, D. Yu, M. Xiao, E. Moons, F. Zhang, T. D. Anthopoulos, O. Inganäs and E. Wang, *Energy Environ. Sci.*, 2020, **13**, 5017–5027.
29 J.-W. Lee, C. Sun, B. S. Ma, H. J. Kim, C. Wang, J. M. Ryu, C. Lim, T.-S. Kim, Y.-H. Kim, S.-K. Kwon and B. J. Kim, *Advanced Energy Materials*, 2021, **11**, 2003367.
30 H. Yu, Y. Wang, H. K. Kim, X. Wu, Y. Li, Z. Yao, M. Pan, X. Zou, J. Zhang, S. Chen, D. Zhao, F. Huang, X. Lu, Z. Zhu and H. Yan, *Advanced Materials*, 2022, **34**, 2200361.
31 H. Yu, Z. Qi, J. Yu, Y. Xiao, R. Sun, Z. Luo, A. M. H. Cheung, J. Zhang, H. Sun, W. Zhou, S. Chen, X. Guo, X. Lu, F. Gao, J. Min and H. Yan, *Advanced Energy Materials*, 2021, **11**, 2003171.
32 H. Yu, M. Pan, R. Sun, I. Agunawela, J. Zhang, Y. Li, Z. Qi, H. Han, X. Zou, W. Zhou, S. Chen, J. Y. L. Lai, S. Luo, Z. Luo, D. Zhao, X. Lu, H. Ade, F. Huang, J. Min and H. Yan, *Angewandte Chemie International Edition*, 2021, **60**, 10137–10146.
33 T. Wang, R. Sun, X.-R. Yang, Y. Wu, W. Wang, Q. Li, C.-F. Zhang and J. Min, *Chin J Polym Sci*, 2022, **40**, 877–888.
34 H. Fu, Q. Fan, W. Gao, J. Oh, Y. Li, F. Lin, F. Qi, C. Yang, T. J. Marks and A. K.-Y. Jen, *Sci. China Chem.*, 2022, **65**, 309–317.
35 H. Fu, Y. Li, Z. Wu, F. R. Lin, H. Y. Woo and A. K.-Y. Jen, *Macromolecular Rapid Communications*, 2022, **43**, 2200062.
36 Y. Yue, B. Zheng, W. Yang, L. Huo, J. Wang and L. Jiang, *Advanced Materials*, 2022, **34**, 2108508.
37 W. Xu, M. Zhang, X. Ma, X. Zhu, S. Y. Jeong, H. Y. Woo, J. Zhang, W. Du, J. Wang, X. Liu and F. Zhang, *Advanced Functional Materials*, 2023, **33**, 2215204.
38 Z. Li, L. Ying, P. Zhu, W. Zhong, N. Li, F. Liu, F. Huang and Y. Cao, *Energy Environ. Sci.*, 2019, **12**, 157–163.
39 B. Fan, L. Ying, Z. Wang, B. He, X.-F. Jiang, F. Huang and Y. Cao, *Energy Environ. Sci.*, 2017, **10**, 1243–1251.
40 K. Feng, Z. Wu, M. Su, S. Ma, Y. Shi, K. Yang, Y. Wang, Y. Zhang, W. Sun, X. Cheng, L. Huang, J. Min, H. Y. Woo and X. Guo, *Advanced Functional Materials*, 2021, **31**, 2008494.
41 J. Zhang, C.-H. Tan, K. Zhang, T. Jia, Y. Cui, W. Deng, X. Liao, H. Wu, Q. Xu, F. Huang and Y. Cao, *Advanced Energy Materials*, 2021, **11**, 2102559.
42 H.-R. Bai, H. Zhang, H. Meng, Y. Li, X. Xu, M.-Q. Liu, Y. Chen, Z.-F. Yao, H.-F. Zhi, A. Mahmood, Y. Wang, J.-H. Ye, M. Jiang, Q. An, H. Y. Woo, H. Wu, Q. Peng and J.-L. Wang, *Materials Science and Engineering: R: Reports*, 2025, **163**, 100916.
43 W. Qiu, C. Liao, Y. Li, M. Deng, Y. Duan, X. Xu and Q. Peng, *Advanced Functional Materials*, 2025, **35**, 2503009.
44 R. Zeng, L. Zhu, M. Zhang, W. Zhong, G. Zhou, J. Zhuang, T. Hao, Z. Zhou, L. Zhou, N. Hartmann, X. Xue, H. Jing, F. Han, Y. Bai, H. Wu, Z. Tang, Y. Zou, H. Zhu, C.-C. Chen, Y. Zhang and F. Liu, *Nat Commun*, 2023, **14**, 4148.
45 Z. Wang, X. Wang, L. Tu, H. Wang, M. Du, T. Dai, Q. Guo, Y. Shi and E. Zhou, *Angewandte Chemie International Edition*, 2024, **63**, e202319755.
46 R. Sun, W. Wang, H. Yu, Z. Chen, X. Xia, H. Shen, J. Guo, M. Shi, Y. Zheng, Y. Wu, W. Yang, T. Wang, Q. Wu, Y. (Michael) Yang, X. Lu, J. Xia, C. J. Brabec, H. Yan, Y. Li and J. Min, *Joule*, 2021, **5**, 1548–1565.
47 H. Yu, C. Zhao, H. Hu, S. Zhu, B. Zou, T. A. D. Peña, H. M. Ng, C. H. Kwok, J. Yi, W. Liu, M. Li, J. Wu, G. Zhang, Y. Chen and H. Yan, *Energy Environ. Sci.*, 2024, **17**, 5191–5199.
48 T. Wang, R. Sun, W. Wang, H. Li, Y. Wu and J. Min, *Chem. Mater.*, 2021, **33**, 761–773.
49 Q. Wu, W. Wang, Y. Wu, R. Sun, J. Guo, M. Shi and J. Min, *Natl Sci Rev*, 2022, **9**, nwab151.
50 H. Yu, Y. Wang, H. K. Kim, X. Wu, Y. Li, Z. Yao, M. Pan, X. Zou, J. Zhang, S. Chen, D. Zhao, F. Huang, X. Lu, Z. Zhu and H. Yan, *Advanced Materials*, 2022, **34**, 2200361.

51 R. Sun, T. Wang, Q. Fan, M. Wu, X. Yang, X. Wu, Y. Yu, X. Xia, F. Cui, J. Wan, X. Lu, X. Hao, A. K.-Y. Jen, E. Spiecker and J. Min, *Joule*, 2023, **7**, 221–237.
52 C. Cao, H. Wang, D. Qiu, T. Zhao, Y. Zhu, X. Lai, M. Pu, Y. Li, H. Li, H. Chen and F. He, *Advanced Functional Materials*, 2022, **32**, 2201828.
53 T. Wang, R. Sun, X.-R. Yang, Y. Wu, W. Wang, Q. Li, C.-F. Zhang and J. Min, *Chin J Polym Sci*, 2022, **40**, 877–888.
54 X. Yang, R. Sun, Y. Wang, M. Chen, X. Xia, X. Lu, G. Lu and J. Min, *Advanced Materials*, 2023, **35**, 2209350.
55 G. Sun, X. Jiang, X. Li, L. Meng, J. Zhang, S. Qin, X. Kong, J. Li, J. Xin, W. Ma and Y. Li, *Nat Commun*, 2022, **13**, 5267.
56 J. Du, K. Hu, L. Meng, I. Angunawela, J. Zhang, S. Qin, A. Liebman-Pelaez, C. Zhu, Z. Zhang, H. Ade and Y. Li, *Angewandte Chemie International Edition*, 2020, **59**, 15181–15185.
57 H. Wang, C. Cao, H. Chen, H. Lai, C. Ke, Y. Zhu, H. Li and F. He, *Angewandte Chemie International Edition*, 2022, **61**, e202201844.
58 Y. Liang, D. Zhang, Z. Wu, T. Jia, L. Lüer, H. Tang, L. Hong, J. Zhang, K. Zhang, C. J. Brabec, N. Li and F. Huang, *Nat Energy*, 2022, **7**, 1180–1190.
59 Y. Yue, B. Zheng, W. Yang, L. Huo, J. Wang and L. Jiang, *Advanced Materials*, 2022, **34**, 2108508.
60 K. Hu, C. Zhu, S. Qin, W. Lai, J. Du, L. Meng, Z. Zhang and Y. Li, *Science Bulletin*, 2022, **67**, 2096–2102.
61 W. Xu, M. Zhang, X. Ma, X. Zhu, S. Y. Jeong, H. Y. Woo, J. Zhang, W. Du, J. Wang, X. Liu and F. Zhang, *Advanced Functional Materials*, 2023, **33**, 2215204.
62 Z. Wang, Y. Guo, X. Liu, W. Shu, G. Han, K. Ding, S. Mukherjee, N. Zhang, H.-L. Yip, Y. Yi, H. Ade and P. C. Y. Chow, *Nat Commun*, 2024, **15**, 1212.
